# The interaction between protein kinase A and progesterone on basal and inflammation-induced myometrial oxytocin receptor expression

**DOI:** 10.1371/journal.pone.0239937

**Published:** 2020-12-01

**Authors:** Angela Yulia, Alice J. Varley, Natasha Singh, Kaiyu Lei, Rachel M. Tribe, Mark R. Johnson

**Affiliations:** 1 Institute of Reproductive and Developmental Biology, Imperial College School of Medicine, Chelsea and Westminster Hospital, London, United Kingdom; 2 Department of Women and Children’s Health, School of Life Course Sciences, Kings College London, London, United Kingdom; John Hunter Hospital, AUSTRALIA

## Abstract

Our previous work has shown myometrial PKA activity declines in term and twin-preterm labour in association with an increase in the expression of the oxytocin receptor (OTR). Here we investigate the action of cAMP/PKA in basal conditions, with the addition of progesterone (P4) and/or IL-1β to understand how cAMP/PKA acts to maintain pregnancy and whether the combination of cAMP and P4 would be a viable therapeutic combination for the prevention of preterm labour (PTL). Further, given that we have previously found that cAMP enhances P4 action we wanted to test the hypothesis that changes in the cAMP effector system are responsible for the functional withdrawal of myometrial P4 action. Myometrial cells were grown from biopsies obtained from women at the time of elective Caesarean section before the onset of labour. The addition of forskolin, an adenylyl cyclase activator, repressed basal OTR mRNA levels at all doses and P4 only enhanced this effect at its highest dose. Forskolin repressed the IL-1β-induced increase in OTR mRNA and protein levels in a PKA-dependent fashion and repressed IL-1β-activation and nuclear transfer of NFκB and AP-1. P4 had similar effects and the combination P4 and forskolin had greater effects on OTR and NFκB than forskolin alone. While PKA knockdown had no effect on the ability of P4 to repress IL-1β-induced OTR expression it reversed the repressive effect of the combination of P4 and forskolin and resulted in a greater increase than observed with IL-1β alone. These studies suggest that cAMP acts via PKA to repress inflammation-driven OTR expression, but that when PKA activity is reduced, the combination of cAMP and P4 actually enhances the OTR response to inflammation, promoting the onset of labour and suggesting that changes in the cAMP effector system can induce a functional P4 withdrawal.

## Introduction

Globally, preterm labor (PTL) remains the most important cause of childhood mortality [1] and current approaches to its management are relatively ineffective [2]. Our ability to prevent preterm labour has been hindered by poor understanding of the factors that initiate human parturition. Our recent paper described that term early labour is associated with a decline in myometrial PKA activity and an increase in the key pro-labor factor, oxytocin receptor (OTR) [3]. We found that with the reduction in myometrial PKA activity, myometrial cAMP signals through exchange proteins directly activated by cAMP (Epac1) to increase both OTR gene expression and protein levels and confirmed that the increase in OTR increased the response to oxytocin [3]. These data are significant, as they present a plausible explanation for the increase in OTR expression and the onset of labour. They are all the more important as in the paper paired with this publication we demonstrate that similar changes in the cAMP effector system occur in twin early PTL (Yulia et al., unpublished observation). Together, our observations offer the prospect of therapeutic targets upstream of the OTR, the inhibition of which is only moderately effective in arresting PTL once it has started [4].

In our previous work, we observed that cAMP enhanced P4 repression of inflammation in primary myometrial cells [5]. Others have reported that cAMP enhances agonist bound PR activity [6–8] and converts the PR antagonist, RU486, into a partial agonist [6–8]. Further, physiologically, the combination of cAMP and P4 enhances endometrial stromal cell decidualisation to a greater extent than either agent alone [9]. These data suggest that myometrial cAMP may be able to modulate P4 action. If the myometrial cAMP system does enhance P4 action via PKA, then understanding the mechanisms involved might suggest a potential therapeutic approach to enhance P4 action. Equally, given the decline in myometrial PKA activity observed at term by several groups, ours included [3, 10, 11], understanding the interaction between cAMP/PKA and P4 may offer an explanation for the loss of P4 action with the onset of term labour and possibly PTL.

Inflammation has long been held to play an important role in the onset of term and PTL. However, our data at term suggest that myometrial inflammation is actually a consequence rather than a cause of labour [3]. Equally, we found that NFκB activity was increased in early labour and our work in PTL samples has identified a dominant role for inflammation in chorioamnionitis across all reproductive tissues and to a lesser extent in twin and idiopathic PTL [12]. The suggestion of a link between twin PTL and inflammation is consistent with our *in vivo* data using a stretch-induced labour model in the non-human primate that suggests that stretch induces a significant inflammatory pulse [13] and our *in vitro* data in which stretch of myometrial cells increases pro-labour gene expression, COX-2 and OTR in association with increased with activation of the inflammatory transcription factors NFκB and AP-1 [14–16].

In this paper, we set out to study the action of cAMP acting via PKA on basal and IL-1β-induced OTR expression and its interaction with P4. We tested the hypotheses that changes in the cAMP effector system, in particular, a decline in PKA action, modulates the ability of P4 to repress inflammation-induced gene expression.

## Materials and methods

Approval for the collection of myometrial biopsies was obtained from London-Chelsea Committee 10/H0801/45. Fully informed written consent was sought from all patients participating in the study.

### Myometrial cultures

Fully informed written consent was obtained from all patients participating in the study. Myometrial biopsies (0.5 x 0.5 x 0.5 cm^3^) were taken from the upper margin of the uterine lower segment incision at the time of uncomplicated Caesarean section from non-laboring women at term Chelsea and Westminster Hospital (London, United Kingdom). Women were recruited from the term not in labor (TNL) (>37 weeks, demographic data in S1 Table in S1 File) group. The indications for lower segment Caesarean section included previous Caesarean section, breech presentation and maternal request.

Myometrial biopsies were placed into Dulbecco’s modified Eagle’s Medium (DMEM; Invitrogen, Paisley, UK) supplemented with 1% L-glutamine and 1% penicillin-streptomycin and were stored at 4°C for no more than 3 hours prior to preparation for cell culture [17]. The biopsies were minced and digested for about 45 minutes at 37°C in a collagenase solution containing 0.5 mg/mL collagenase 1A and XI (Sigma, Dorset, UK) and 1 mg/mL bovine serum albumin in DMEM. The digestion process was stopped by the addition of DMEM supplemented with 7.5% fetal calf serum (FCS; Gibco, Paisley, UK). The myometrial tissue suspension was agitated to further disperse the cells. The resulting suspension was then passed through a 70μm cell strainer and individual cells were collected by centrifugation at 800×*g* for 5 minutes. The media was aspirated and the cell pellet was resuspended and grown in DMEM with supplementation of 7.5% FCS, 1% L-glutamine and 1% penicillin-streptomycin at 37°C in an atmosphere consisting of 5% CO_2_. The medium was changed at least three times per week. The myometrial cells used were either in their third or fourth passages [17, 18]. The cells were serum starved in the starvation medium (DMEM supplemented with 1% charcoal-stripped FCS 1% L-glutamine and 1% penicillin-streptomycin), approximately 16–24 hours prior to initiation of experiments. In all cases, at the end of the specified time, the medium was aspirated and the cells were frozen and stored at -80°C for mRNA and whole-cell protein extraction. Each *n* represents the use of cells isolated from an individual woman.

### Cells RNA extraction, cDNA synthesis and qPCR

Total RNA was extracted and purified from myometrial cells grown in 6-well plates using an RNAeasy kit (Qiagen Ltd, UK). The samples were nano-dropped to determine the RNA concentration. Following quantification, 1.5μg of RNA was reverse transcribed with oligo DT random primers and MuLV reverse transcriptase using the QuantiTect Reverse Transcription kit (Qiagen, Manchester, UK). Primer sets [S2 Table in S1 File] were designed and purchased from Invitrogen (Paisley, UK). Quantitative PCR was performed in the presence of SYBR Green (Roche, West Sussex, UK) using RotorGene Q thermocycler (Qiagen, Manchester, UK). The pre-PCR cycle occurred for 10 minutes at 95°C followed by up to 45 cycles at 95°C for 20 seconds, 58–60°C for 20 seconds and 72°C for 20 seconds, with an extension at 72°C for 15 seconds. The final process involves a melt over a temperature range of 72–99°C rising by 1°C steps, followed by a wait of 15 seconds on the first step and a wait of 5 seconds for each subsequent step. The cycle threshold (Ct) was used for quantitative analysis, which denotes the cycle at which fluorescence reaches a preset threshold. The cycle threshold is set at a level whereby the exponential increase in amplicon yield is approximately parallel between the samples. All mRNA abundance data were expressed relative to the amount of the constitutively expressed glyceraldehyde-3-phosphate dehydrogenase (GAPDH). We use GAPDH as a housekeeping gene in our PCR as shown in the manuscript. We have trialled multiple housekeeping genes in previous our studies [19]. GAPDH mRNA expression in human tissues has been extensively studied by others [20–22], and has been shown to be reliable and widely expressed in human tissues. GAPDH has demonstrated great stability and is one of the best housekeeping gene for normalisation in the use of reproductive tissues, mainly in the placenta and myometrium. In addition, the GAPDH level was stable in its amplification at different kinds of stresses, thus making it a reliable housekeeping gene due to its stability at these different conditions.

### Transient siRNA transfection

Cells grown to about 80% confluence were trypsinised and transfected using primary smooth muscle cells nucleofector kit transfection reagent (Lonza, Slough, UK) according to the manufacturer’s protocol and the transfected cells were seeded onto 6-well plates. Based on preliminary transfection experiments, after transfection of the relevant siRNAs (S3 Table in S1 File) for 24 hours, the medium was changed to one containing 7.5% FCS, then after a further 48 hrs, the cells were serum starved. The next day, the cells were treated under different conditions for 6 hours. At the end of the specified time, the medium was removed and the cells were frozen at -80°C for extraction of mRNA and whole-cell protein.

### Cell protein extraction

Frozen cells from -80°C were taken and lysed in cell lysis buffer (New England BioLabs, Hitchin, UK). Complete protease inhibitor tablets (Roche) were added to the buffer. The cells were scraped and collected in Eppendorf tubes. The samples were centrifuged at 13,000×*g* for 15 minutes at 4°C. Supernatant were then transferred and stored at -80°C for western blotting. The primary antibodies are listed on the S4 Table in S1 File.

### Cytosolic/nuclear protein extraction

At the end of the specified treatment time points, medium was aspirated. The cells were then washed with sterile PBS once. The cells were trypsinised and centrifuged at 800g for 5 minutes 4°C. The cell pellet was resuspended with buffer A supplemented with protease and phosphatase inhibitors and incubated on ice for 20 minutes. Cytosolic buffer A consists of 10mM HEPES, 10 mM KCl, 0.1 mM EDTA, 0.1 mM EGTA, 2 mM DTT and 1% (v/v) NP-40. 20% of NP-40 alternative was added to the lysates and were vortexed for 10 seconds at a high speed followed by centrifugation at 16,000×*g* for 30 seconds at 4°C. The supernatants were reserved as the cytosolic protein extracts. The pellets were re-suspended in buffer B supplemented with protease and phosphatase inhibitors and incubated on ice for 15 minutes with shaking (~200 rpm). Nuclear buffer B consists of 10 mM HEPES, 10 mM KCl, 0.1 mM EDTA, 0.1 mM EGTA, 2 mM DTT, 400 mM NaCl, and 1% NP-40 (v/v). Subsequently, the suspension was centrifuged for 5 minutes at 16,000×*g* at 4°C. After the last centrifugation, the supernatant (nuclear extract) was transferred to a separate tube. These were stored at -80°C for western blotting, which was performed as described above.

### Statistical analysis

For all of our data (RT-PCR and western protein data), they were initially tested for normality using a Kolmogorov–Smirnoff test or a D’Agostino & Pearson normality test depending on the group size. The normally distributed data were analysed using a Student’s t-test for two groups or an ANOVA followed by a Dunnett’s or Bonferroni’s post-hoc test for three groups or more. Data which were not normally distributed were analysed using a Wilcoxon matched pair or a Mann-Whitney test for unpaired data for 2 groups or a Friedman’s test with a Dunn’s multiple comparisons post-hoc test to compare three groups or more. P <0.05 was considered statistically significant. We used GraphPad Prism 7.0 software to generate graphical representations of the data.

## Results

### The basal effects of the combination of forskolin and progesterone

Forskolin at all doses (1, 10 and 100μM) repressed OTR mRNA levels equally (all p<0.05; S1A Fig). P4 had no effect (S1B Fig). Only at 24 hours did the combination of P4 10μM and forskolin 100μM repress basal *OTR* mRNA more than forskolin alone (p<0.05; (all p<0.05; S1D Fig)). P4 did not enhance forskolin reduction in the mRNA levels of cAMP-repressed genes (S2A–S2E Fig) but for the cAMP-increased genes, P4 enhanced the forskolin-induced increase in *11βHSD* type 1 at 6, 24 and 48 hours, *PTGES* at 24 hours and *GPR125* at 48 hours (p<0.05–0.01; S2G, S2J&S2K Fig).

### The anti-inflammatory and progestational effects of forskolin

**Fig 1:**
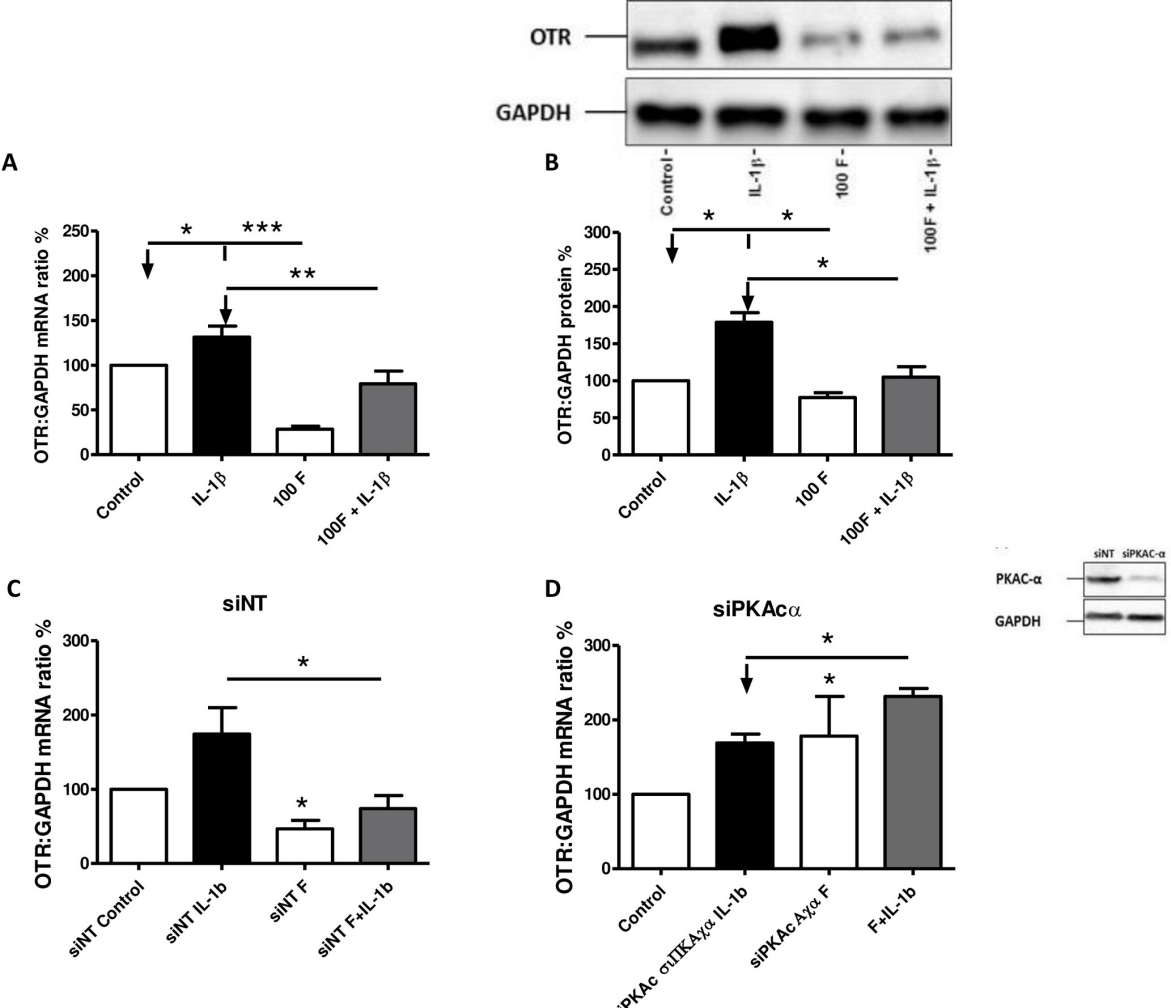
The effect of forskolin on the IL-1β-induced increase in OTR mRNA and protein levels and the impact of PKA knockdown. Myometrial cells were isolated as described above in *Materials and Methods*, and treated with IL-1β (1ng/mL) and/or forskolin (100μM) either alone or in combination for 6 hours. mRNA and protein were extracted, and the levels of OTR (A) mRNA were measured using quantitative rt-PCR. The levels of OTR (B) protein were measured using Western blotting. A representative western blot is shown next to the graph displaying the densitometry of the protein levels. PKAC-α was knocked down using siRNA (siPKAC-α) controlled with non-targeted siRNA [siNT]. Representative western blots to demonstrate transfection is shown above. After transfection, cells were incubated for 96 hours before being treated with progesterone (10μM), IL-1β (1ng/mL) and forskolin (100μM) in combination for 6 hours. The mRNA was extracted, and the levels of OTR (C, D), mRNA were measured using rt-PCR. Data are shown as the mean and SEM. A and B were compared using Wilcoxon matched pairs test for data that were not normally distributed and paired *t* test for data that were normally distributed. C and D were compared using a non-parametric paired test on the basis that we wanted to understand the impact of blocking PKA. *P<0.05, **P<0.01, ***P<0.001 (n = 8–9 myometrial samples from 8–9 different women in each experiment).

In primary myometrial cell cultures, IL-1β (1ng/ml) increased both the mRNA and protein levels of OTR (both p<0.05; Fig 1A & 1B). Forskolin (100μM) treatment alone and in combination with IL-1β repressed OTR mRNA and protein levels (basal both p<0.05 and in combination with IL-1β (p<0.01 and 0.05 Fig 1A & 1B). With PKA knockdown, the inhibitory effect of forskolin on both basal and IL-1β-stimulated OTR mRNA levels was reversed, such that OTR mRNA levels were increased in both circumstances (p<0.01 and 0.05 Fig 1C & 1D). The knockdown of Epac1 had no effect on forskolin repression of basal or stimulated OTR mRNA levels (S3C Fig) and of AMPK only blunted the basal repression (S3D Fig).

**Fig 2:**
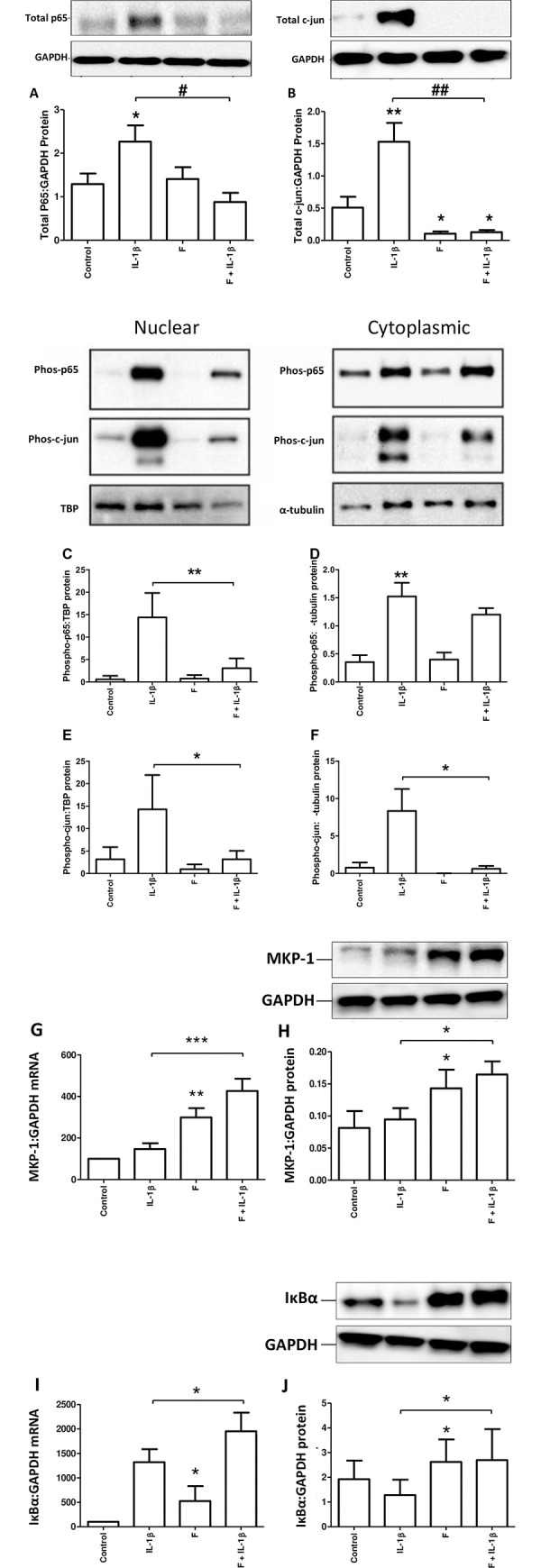
Forskolin represses IL-1β-induced total and nuclear and cytosolic p65 protein and c-jun levels. Myometrial cells were isolated from myometrial biopsies obtained from women at the time of pre-labor term Caesarean section as described above in *Materials and Methods*, and treated with IL-1β (1ng/mL) and/or forskolin (100μM) either alone or in combination for 6 hours. Protein was extracted, and the levels of total p65 (A), and total c-jun (B) protein expression were measured using western blotting. Myometrial cells were isolated as described above in *Materials and Methods*, and treated with forskolin (100μM) or IL-1β (1ng/mL) either alone or in combination for 1 hour. Cells were lysed and samples purified for cytoplasmic or nuclear protein. Western blotting was performed using antibodies directed against phospho-p65 (Ser536) (C, D), and phospho c-jun (E, F). TATA-binding protein (TBP) and α-tubulin were used as the internal controls for nuclear and cytosolic fraction, respectively. MKP-1 (G, H) and IKBα (I, J) mRNA and protein levels were measured using rt-PCR and western blotting respectively. Data are shown as the mean and SEM, and were compared using (control vs. forskolin and IL-1β alone vs. IL-1β + forskolin) Wilcoxon matched pairs test for data that were not normally distributed and paired *t* test for data that were normally distributed. *P<0.05, **P<0.01, ***P<0.001 when compared to control. (n = 6–7 myometrial samples from 6–7 different women in each experiment).

As both NFKB and AP-1 transcription factor systems have been implicated in OTR expression, we assessed the ability of forskolin to modulate the basal and IL-1β-induced activation of both systems. Forskolin had no effect on basal p65 but repressed basal c-jun (p<0.05; Fig 2A&2B) and the IL-1β-induced increase in both p65 (p<0.05; Fig 2A) and c-jun (p<0.01; Fig 2B). Forskolin also reduced the IL-1β increased phosphorylation and nuclear transfer of both p65 and c-jun (p<0.01 and 0.05 respectively; Fig 2C&2E). Forskolin alone and in combination with IL-1β increased the mRNA and protein levels of MKP-1 and IκB (p<0.05–0.001; Fig 2G–2J).

### The anti-inflammatory and progestational effects of the combination of forskolin and progesterone

The IL-1β-induced increase in *OTR* mRNA levels were repressed to a similar degree by all doses of P4 in combination with forskolin 100μM, consequently, further studies were undertaken using the combination of forskolin 100μM and P4 10μM (S4 Fig).

#### OTR

**Fig 3:**
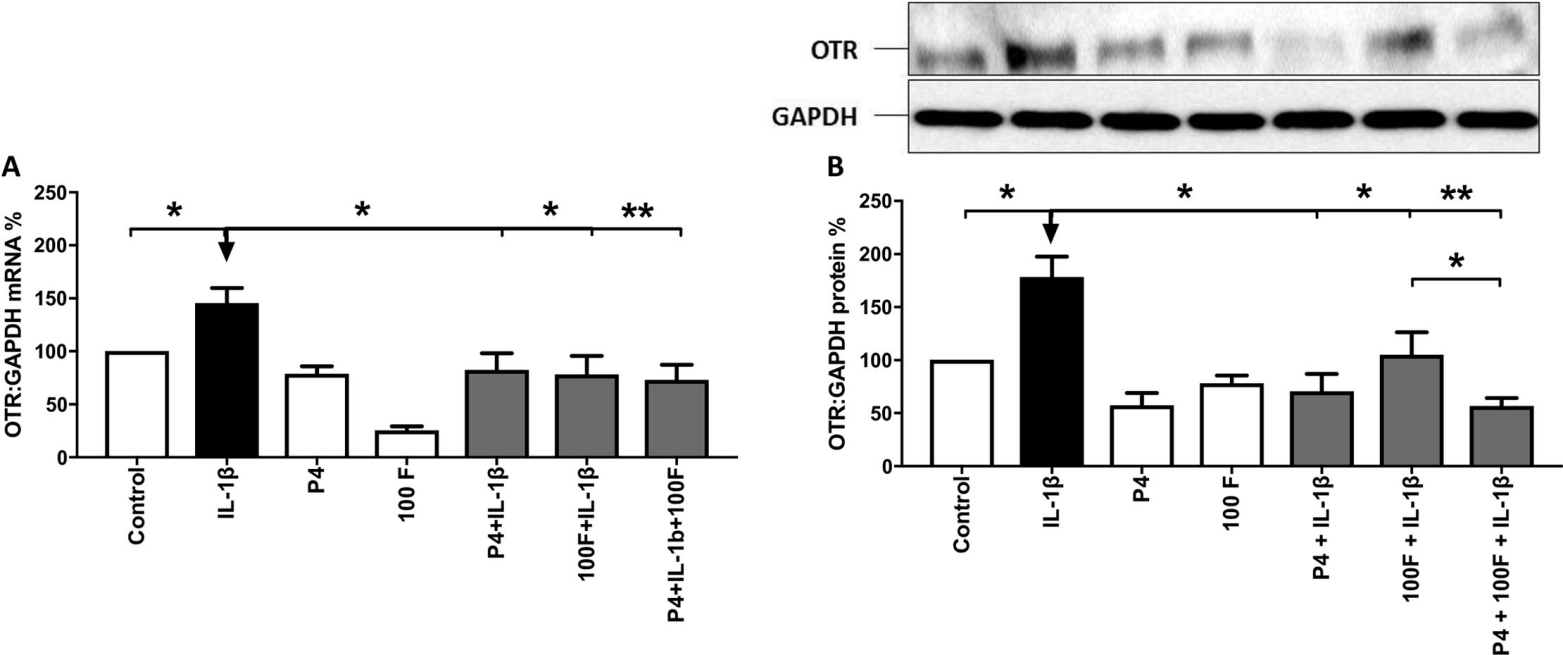
The effect of the combination of forskolin and progesterone on the IL-1β-induced increase in OTR mRNA and protein levels. Myometrial cells were isolated as described in *Materials and Methods*, and treated with progesterone (10μM), forskolin (100μM) or IL-1β (1ng/mL) either alone or in combination for 6 hours. mRNA and protein were extracted, and the levels of OTR (A) mRNA were measured using quantitative rt-PCR and the levels of OTR (B) protein were measured using Western blotting. A representative western blot is shown next to the graph displaying the densitometry of the protein levels. Data are expressed as mean SEM and were compared using Friedman’s Test, with a Dunn's Multiple Comparisons post hoc test for data that were not normally distributed, and using ANOVA, with Dunnett and Bonferroni’s post-test for data that were normally distributed, *P<0.05, **P<0.01 (n = 8–9 myometrial cells from 8–9 different women).

The combination of forskolin and P4 repressed the IL-1β-induced increase in *OTR* mRNA levels, however, for *OTR* mRNA levels, the effect of the combination was no greater than either agent alone, while for OTR protein levels, the combination had a greater effect than forskolin alone (p<0.05; Fig 3A & 3B). A PKA-specific agonist, sp-6-phe-cAMP, in combination with P4 had similar effects to forskolin on the IL-1β-induced increases in *OTR* mRNA (S5 Fig).

#### AP-1 and NFκB

**Fig 4:**
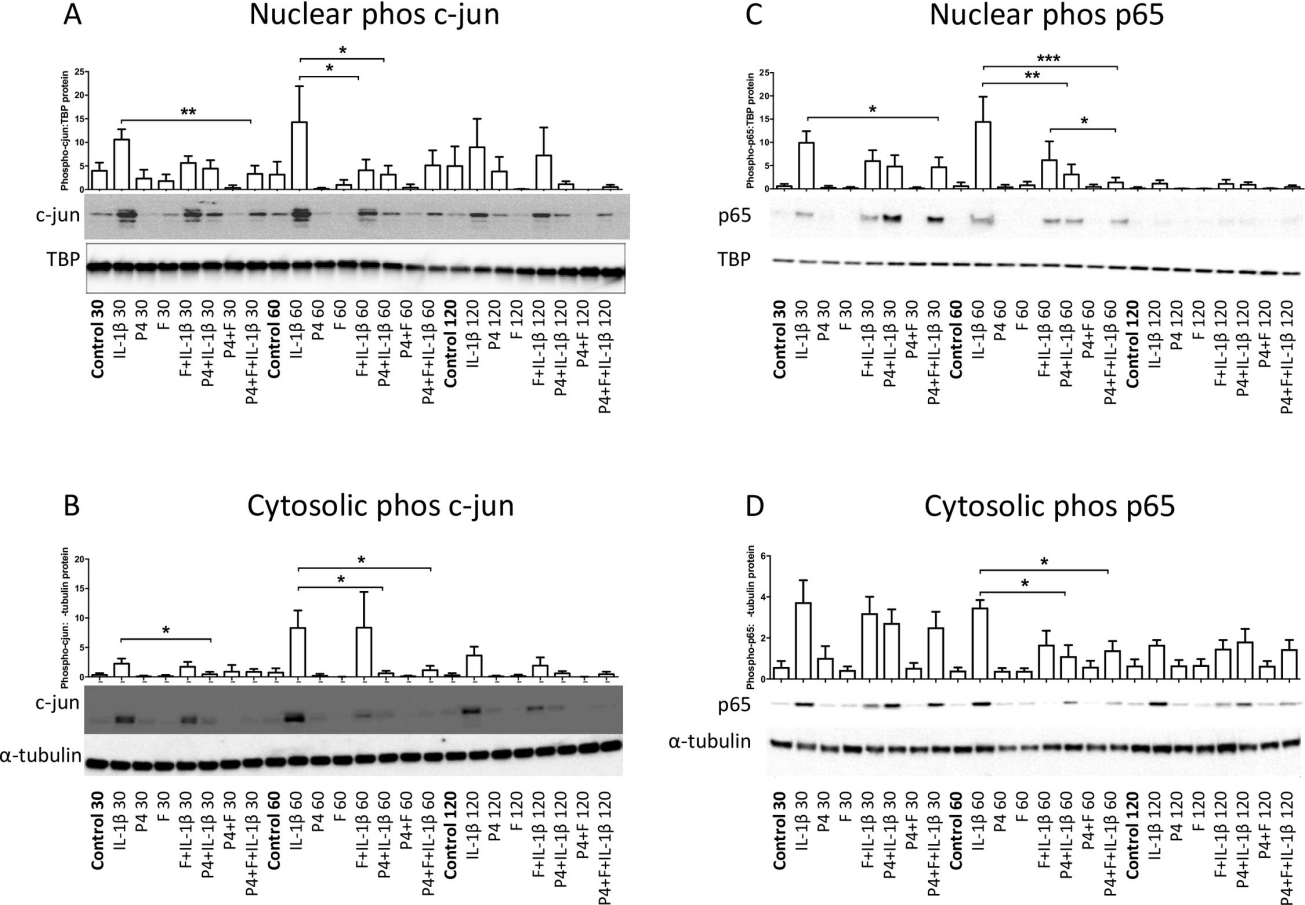
The combination of cAMP with progesterone represses IL-1β-induced nuclear transfer of phospho c-jun and phospho p65. Myometrial cells were isolated from myometrial biopsies obtained from women at the time of pre-labor term Caesarean section as described above in *Materials and Methods*, and treated with progesterone (10μM), forskolin (100μM) or IL-1β (1ng/mL) either alone or in combination for for 0 minute, 30 minutes, 1 hour, 2 hours and 6 hours. Cells were lysed and samples purified for cytoplasmic or nuclear protein. Western blotting was performed using antibodies directed against phospho c-jun (A, B), and phospho-p65 (Ser536) (C, D). α-tubulin and TATA-bind protein (TBP) were used as the internal controls for cytosolic and nuclear fraction, respectively. Data were compared using Friedman’s Test, with a Dunn's Multiple Comparisons post hoc test for data that were not normally distributed, and using ANOVA, with Dunnett and Bonferroni’s post-test for data that were normally distributed, *P<0.05, **P<0.01, ***P<0.001. (n = 6 myometrial cells from 6 different women and only 30 minutes, 1 hour, and 2 hours time points were shown in the figure above).

*In* terms of the effects on c-Jun and p65 phosphorylation, the combination of forskolin and P4 repressed the IL-1β-induced increase in c-Jun and p65 phosphorylation in both cytosolic and nuclear compartments at 1 hour. However, the combination only had a greater effect than P4 alone for nuclear p65 phosphorylation levels (p<0.05; Fig 4C). For IL-1β-induced JNK and p38 activation, P4 had no effect, forskolin alone reduced both JNK and p38 activation and the addition of P4 had no greater effect (both p<0.05, S6A&S6B Fig).

#### MKP-1 and IκB

As we observed earlier at 6 hours, forskolin increased MKP-1 levels peaking at 60 minutes, this was not further increased by the addition of either IL-1β or P4 (S7A Fig). In terms of IκB, the repressive effect of IL-1β (maximal at 30 minutes) was unaffected by forskolin, P4 or their combination (S7B Fig); however, the re-accumulation was faster in the presence of the combination of P4 and forskolin at 1 and 2 hours (p<0.05 and 0.01 respectively, S7B Fig).

### The impact of cAMP effector knockdown on the anti-inflammatory and progestational effects

**Fig 5:**
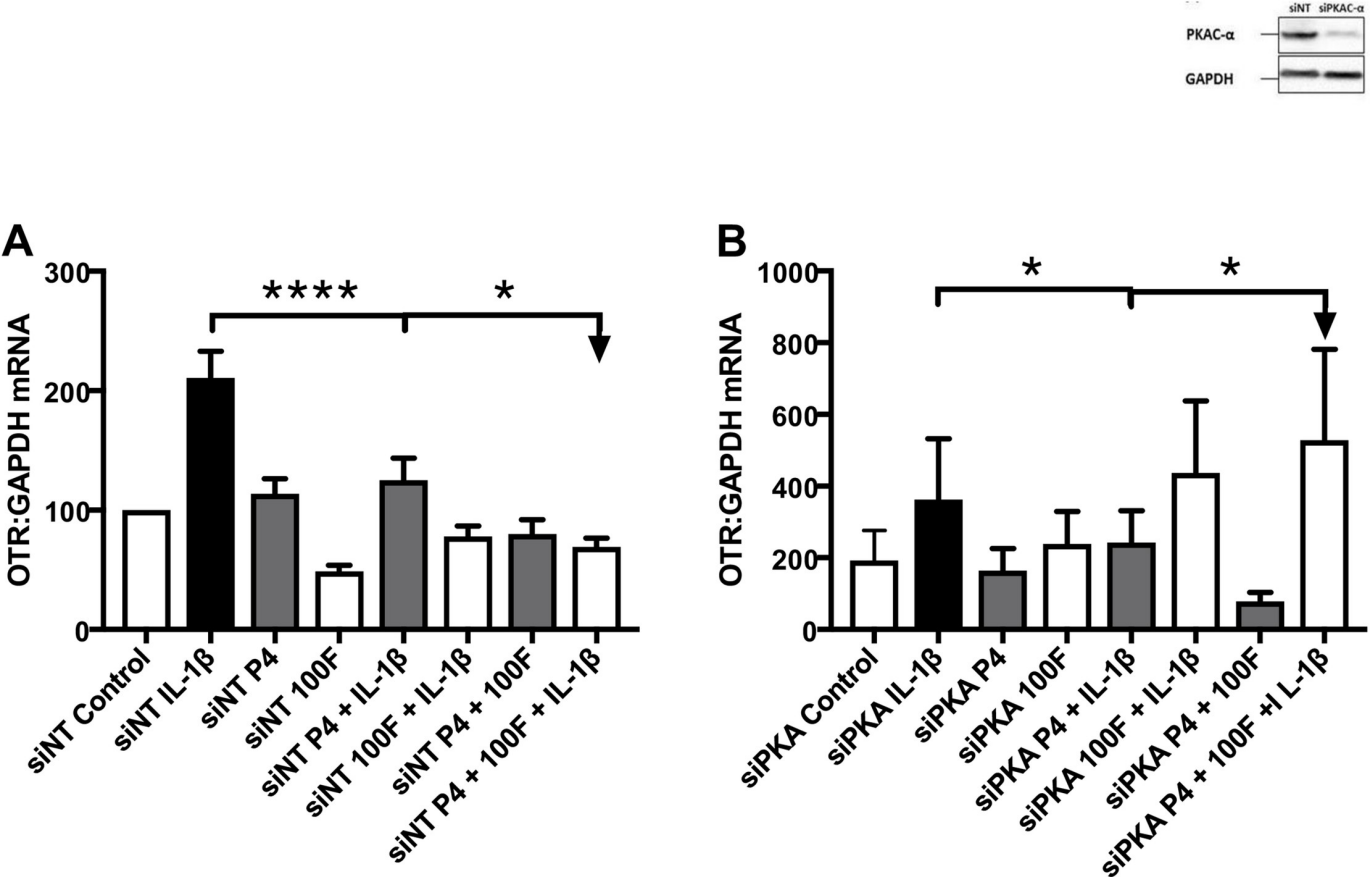
The effect of PKA knockdown on the repression of the IL-1β-induced increase in OTR mRNA and protein levels by forskolin and progesterone. Myometrial cells were isolated from myometrial biopsies obtained from women at the time of pre-labor term Caesarean section as described above in *Materials and Methods*. After the cells were about 80% confluent, PKAC-α were knocked down using siRNA (siPKAC-α, efficacy shown in inset figure) controlled with non-targeted siRNA [siNT]. Cells were knocked down using siNT. Representative western blots to demonstrate transfection is shown above. After transfection, cells were incubated for 96 hours before being treated with progesterone (10μM), IL-1β (1ng/mL) and forskolin (100μM) in combination for 6 hours. The mRNA was extracted, and the levels of OTR (A, B) mRNA were measured using rt-PCR. Data were compared using Friedman’s Test, with a Dunn's Multiple Comparisons post hoc test for data that were not normally distributed, and using ANOVA, with Dunnett and Bonferroni’s post-test for data that were normally distributed, *P<0.05, **P<0.01, ***P<0.001, ****P<0.0001 (n = 6–7 myometrial cells from 6–7 different women).

PKA knockdown reversed the repressive effect of forskolin and the combination of forskolin and P4 on the IL-1β-induced increase in *OTR* mRNA levels (p<0.05; Fig 5). Knockdown of other cAMP-effectors did not influence the ability of forskolin and the combination of forskolin and P4 to repress the IL-1β-induced increase in *OTR* mRNA levels (S8A–S8D Fig). A PKA antagonist reproduced the effects of siPKA (S9 Fig).

## Discussion

Here we examined the *in vitro* effect of the cAMP/PKA effector system, finding that cAMP represses inflammation and OTR gene expression and that this effect is enhanced by the addition of P4. Strikingly, PKA knockdown reversed the repressive effects of the combination on OTR gene expression, with a several fold increase in *OTR* mRNA levels. These data identify novel therapeutic targets for the prevention of PTL and suggest a potential explanation for the observed functional withdrawal of P4 action with the onset of human labor.

### The pro-pregnancy and anti-inflammatory effects of forskolin alone and with progesterone

Our observed anti-inflammatory effects of cAMP (forskolin) in human myometrial cells is consistent with the findings in other cell types [23–26]. Acutely, forskolin inhibited the IL-1β-induced phosphorylation and nuclear transfer of both c-Jun levels and p65. The acute effects on phosphorylation of c-Jun may be due to the higher levels of MKP-1, but this would not influence total c-Jun levels, which are probably directly repressed. For the NFκB system, cAMP has been reported to inhibit IL-1β-stimulated IκBα degradation [24, 27] and this is probably PKA mediated [28], which could explain our observed decline in p65 phosphorylation and nuclear transfer. Forskolin alone and in combination with IL-1β increased IκBα levels at 6 hours, consistent with the observed greater increase in IκBα mRNA levels but did not reduce IκBα degradation at shorter time points. P4 alone and in combination with forskolin enhanced IκBα re-accumulation but also did not reduce IκBα degradation at shorter time points. Other mechanisms may also be involved, possibly that cAMP, acting via PKA, reduces NFκB DNA binding [29] or modulates the activity of the p65 transactivation domain **[30]**. Indeed, others have described a range of mechanisms through which cAMP inhibits p65/NFκB activity **[23, 24, 26, 31]**.

### The effect of P4/forskolin

Previously, we found that P4 repressed inflammation-induced COX-2 gene expression, but had no effect on IL-8 [32]; here we found that P4 repressed IL-1β-induced increases in OTR mRNA and protein levels. The combination of P4 and forskolin had a greater effect than forskolin alone in terms of protein levels. These data suggest that the combination of a cAMP-agonist and P4 could be effective in delaying the onset of labour. Indeed, in the mouse model of LPS-induced PTL, we found that the combination of aminophylline and P4 delayed the onset of parturition, but did not improve pup survival [33].

P4/PR-B has been shown to repress NFκB in different cell models and via a variety of mechanisms including increasing the levels of the endogenous inhibitor of NFκB, IκBα, and a direct repressive interaction between PR-B and p65 [34–38]. Here we observed a more rapid re-accumulation of IκBα levels by P4 alone and the combination of P4 and forskolin, consistent with the observed reduction in nuclear phospho-p65 by P4 alone and the combination of P4 and forskolin. The effect of the combination tended to be greater than P4 alone, but was not significant.

As previously described, mitogen-activated protein kinase (MAPK) activation has been implicated in the onset of parturition [32]. MKP-1 catalyses the dephosphorylation of active MAPK, and reduces c-jun binding to the COX-2 promoter. Our group demonstrated that progesterone acted via GR to drive mitogen-activated protein kinase phosphatase-1 (MKP-1), which played a significant role in mediating progesterone’s inhibitory effect on IL-1β’s acute actions (1 h) and contributed to progesterone’s inhibitory effect on IL-1β’s intermediate effect (6 h) [32]. The data demonstrated from our group suggests that the anti-inflammatory effects of progesterone are mediated via MKP-1 [32]. Furthermore, MKP-1 is one of the six cAMP up-regulated genes gene, and MKP-1 (mRNA) showed a trend to decline in myometrial samples with advancing gestation and the onset of labour [3]. Thus, the reason why we selected MKP-1 as it may well be possible that the anti-inflammatory effect of cAMP is mediated via MKP-1. Fig 2G&2H showed that treatment with forskolin increases the basal level of MKP-1 mRNA and protein expression compared to control. The combination of forskolin and IL-1β compared to IL-1β alone is associated with a significant increase in MKP-1 mRNA and protein expression. As we observed earlier at 6 hours in Fig 2G&2H, forskolin increased MKP-1 levels peaking at 60 minutes, this was not further increased by the addition of either IL-1β or P4 (S7A Fig).

Our previous data in human myometrial cells and explants, suggested that P4 repressed AP-1 activity by increasing MKP-1 expression [32, 34] and our mouse data show that RU486 administration on day 16 of mouse pregnancy leads to preterm birth approximately 18 hours later with AP-1 activation [39, 40]. Taken with the current results, which showed that P4 inhibited c-Jun phosphorylation and nuclear transfer, the data suggest that P4 is a major repressor of AP-1 activity in myometrial cells. At the transcription factor level, IL-1β-stimulation of total c-Fos, c-Jun and p65 was repressed by P4 and cAMP, but their combination had no greater effect. In terms of phosphorylation and nuclear transfer, P4, cAMP and their combination repressed c-Jun phosphorylation and nuclear transfer, but the combination had no greater effect than either P4 or cAMP alone. Although we did not show a greater effect for the combination in terms of a repressive effect on inflammatory transcription activation, in other circumstances, such as decidualization [9] or in the prevention of LPS-induced PTL [33], the combination of a cAMP agonist and P4 is more effective than either alone, suggesting that cAMP and P4 can act synergistically physiologically and pharmacologically.

### PKA-knockdown produces a functional progesterone withdrawal

The investigation into which cAMP effector mediated the repressive effects of cAMP on IL-1β driven gene expression gave surprising results. We demonstrated that PKA knockdown not only inhibited the repressive effect of the combination of forskolin and P4, but actually reversed it, such that the combination of forskolin and P4 enhanced the actions of IL-1β on OTR. In previous work, we and other authors have demonstrated a decline in PKA levels with advancing gestation [3, 10, 11]. We showed that the decline in PKA levels was associated with a functional impact with reduced CREB phosphorylation and a loss of cAMP/PKA mediated repression of 4 cAMP responsive genes, including OTR [3]. These data are critically important given the effect of PKA knockdown shown here.

Earlier, we found that myometrial cAMP signals through Epac1 to increase both OTR gene expression and protein levels in the presence of low level of myometrial PKA activity [3]. We found that knockdown of Epac1 has minimal effect in the ability of forskolin and the combination of forskolin and P4 to repress the IL-1β-induced increase in *OTR* mRNA levels. AMPK is one of the cAMP effectors and it is one of several potential pathways where cAMP may act to regulate myometrial contractility in human myometrium. Previous study showed that activation of AMPK markedly suppressed both hypertonicity-induced NFκB nuclear translocation and COX-2 activity [41]. In a similar context, Nagata *et al*. [42] recently suggested that AMPK could inhibit the growth of vascular smooth muscle through MEK–ERK pathway inhibition, implying that AMPK could be another cAMP effector in myometrial cells. The role of AMPK in this aspect is not well understood, and thus we investigated the impact of *cAMP effector AMPK on the anti-inflammatory and progestational effects*. Similar to the Epac1 knockdown, we found that knockdown of other cAMP-effector AMPK did not influence the ability of forskolin and the combination of forskolin and P4 to repress the IL-1β-induced increase in *OTR* mRNA levels (S8A–S8D Fig). Together, these findings suggest that the decline in PKA function may contribute to functional withdrawal of P4 action, providing a possible explanation for the onset of human labor despite the maintenance of systemic P4 levels.

These data show that the cAMP system is a key regulator of myometrial OTR levels and, consequently, the onset of labor. Further, that knockdown of PKA reverses the pro-gestational effect of P4 and cAMP in myometrial cells provides compelling evidence of a plausible mechanism for the functional withdrawal of P4.

## Supporting information

S1 Fig Dose response studies for progesterone (0.1, 1.0 or 10μM) and forskolin (100μM) on IL-1β-stimulated OTR expression. Myometrial cells were isolated as described above in *Materials and Methods*, and treated with IL-1β (1ng/ml) alone or in combination with forskolin (100μM) and/or progesterone (0.1, 1.0 or 10μM) for 6 hours. mRNA was extracted, and the levels of OTR mRNA measured using rt-PCR. Data are shown as the mean and SEM, and were compared using Wilcoxon matched pairs test for data that were not normally distributed and paired *t* test for data that were normally distributed. *P<0.05, **P<0.01 (n = 6–9 myometrial samples from 6–9 different women in each experiment).
(PPTX)

S2 Fig The effect of forskolin and progesterone on the mRNA levels of cAMP down- and up-regulated genes. Myometrial cells were isolated from myometrial biopsies obtained from women at the time of pre-labor term Caesarean section as described above in *Materials and Methods*. After the cells were about 80% confluent cells were treated with progesterone (10μM) and/or forskolin (100μM) either alone or in combination for 6, 24 or 48 hours. The mRNA was extracted, and the mRNA levels of down-regulated genes: GUCY1A3, GPR124, CREB3L1, OTR and PRKG1 (A-E) and up-regulated genes: CCL8, 11βHSD1, MKP-1, PDE4B, PTGES and GPR125 (F-K) measured using rt-PCR. Data were compared using Friedman’s Test, with a Dunn's Multiple Comparisons post hoc test for data that were not normally distributed, and using ANOVA, with Dunnett and Bonferroni’s post-test for data that were normally distributed, *P<0.05, **P<0.01 (n = 6–7 myometrial cells from 6–7 different women).
(PPTX)

S3 Fig The effects of cAMP effectors knockdown on the ability of forskolin to repress the IL-1β-induced increase in OTR mRNA. (Fig 3 data are included for comparison) Myometrial cells were isolated from myometrial biopsies obtained from women at the time of pre-labor term Caesarean section as described above in *Materials and Methods*. After the cells were about 80% confluent, cAMP effectors such as PKA, Epac1 and AMPK were knocked down using siRNA (siPKAC-α, siEpac1, siAMPK controlled with non-targeted siRNA [siNT]). Representative western blots to demonstrate the efficacy of knockdown are shown. After transfection, cells were incubated for 96 hours before being treated with IL-1β (1ng/mL) and/or forskolin (100μM) either alone or in combination for 6 hours. The mRNA was extracted, and the levels of OTR mRNA were measured using rt-PCR. The data are expressed as mean SEM and compared using Wilcoxon matched pairs test for data that were not normally distributed and paired *t* test for data that were normally distributed to compare control vs. forskolin alone and IL-1β alone vs. IL-1β and forskolin. *P<0.05, **P<0.01, ***P<0.001 (n = 6–7 myometrial cells from 6–7 different women).
(PPTX)

S4 Fig The effect of the combination of forskolin and different doses of progesterone on the IL-1β-induced increase in OTR mRNA levels. Myometrial cells were isolated as described in *Materials and Methods*, and treated with different doses of progesterone (0.1 μM, 1 μM, 10μM), forskolin (100μM) and IL-1β (1ng/mL) either alone or in combination for 6 hours. mRNA was extracted, and the levels of OTR mRNA were measured using quantitative rt-PCR. Data are expressed as mean SEM and were compared using Friedman’s Test, with a Dunn's Multiple Comparisons post hoc test for data that were not normally distributed, and using ANOVA, with Dunnett and Bonferroni’s post-test for data that were normally distributed, *P<0.05, **P<0.01 (n = 8–9 myometrial cells from 8–9 different women).
(PPTX)

S5 Fig The ability of forskolin (100μM) and a PKA agonist (sp-6-phe-cAMP 100 nM) alone and in combination with progesterone (10μM) to repress IL-1β-stimulated OTR expression. Myometrial cells were isolated as described above in *Materials and Methods*, and treated with (i) IL-1β (1ng/ml) alone or in combination with forskolin (100μM) and/or progesterone (10μM) or with (ii) IL-1β (1ng/ml) alone or in combination with sp-6-phe-cAMP (100nM) and/or progesterone (10μM) for 6 hours. mRNA was extracted, and the levels of OTR mRNA measured using rt-PCR. Data are shown as the mean and SEM, and were compared (IL-1β vs. IL-1β and other treatment combinations) using Friedman’s Test, with a Dunn's Multiple Comparisons post hoc test for data that were not normally distributed, and using ANOVA, with Dunnett and Bonferroni’s post-test for data that were normally distributed. *P<0.05, **P<0.01 (n = 6–9 myometrial samples from 6–9 different women in each experiment).
(PPTX)

S6 Fig The ability of forskolin and progesterone to repress IL-1β activation of JNK and p38. Myometrial cells were isolated from myometrial biopsies obtained from women at the time of pre-labor term Caesarean section as described above in *Materials and Methods*, and treated with progesterone (10μM), forskolin (100μM) or IL-1β (1ng/mL) either alone or in combination for for 30 min. Cells were lysed and protein extracted. Western blotting was performed using antibodies directed against phospho-JNK (A), and phospho p38 (B). α-tubulin was used as the internal control. Data were compared (IL-1β vs. IL-1β and other treatment combinations) using Friedman’s Test, with a Dunn's Multiple Comparisons post hoc test for data that were not normally distributed, and using ANOVA, with Dunnett and Bonferroni’s post-test for data that were normally distributed, P<0.05, n = 6 myometrial cells from 6 different women. *P<0.05 vs. IL-1β.
(PPTX)

S7 Fig The effect of IL-1β, forskolin and progesterone alone and in combination on MKP-1 and IKBα cytoplasmic protein levels. Myometrial cells were isolated from myometrial biopsies obtained from women at the time of pre-labor term Caesarean section as described above in *Materials and Methods*, and treated with progesterone (10μM), forskolin (100μM) or IL-1β (1ng/mL) either alone or in combination for for 0 min, 30 min, 1 h, 2 h and 6 h. Cells were lysed and samples purified for cytoplasmic protein. Western blotting was performed using antibodies directed against MKP-1 (A) and IKBα (B). α-tubulin was used as the internal controls for the cytosolic fraction. MKP-1 data were compared across all groups and for the IKB data, all groups treated with IL-1β were compared using Friedman’s Test, with a Dunn's Multiple Comparisons post hoc test for data that were not normally distributed, and using ANOVA, with Dunnett and Bonferroni’s post-test for data that were normally distributed, P<0.05, **P<0.01, ***P<0.001; n = 6 myometrial cells from 6 different women and only 30 minutes, 1 hour and 2 hours time points are shown).
(PPTX)

S8 Fig The effects of cAMP effector knockdown on the ability of the combination of progesterone and forskolin to reduce the IL-1β-increased OTR mRNA expression. Myometrial cells were isolated from myometrial biopsies obtained from women at the time of pre-labor term Caesarean section as described above in *Materials and Methods*. After the cells were about 80% confluent, cAMP effectors including PKA, Epac1 and AMPK were knocked down using siRNA (siPKA, siEpac1, siAMPK controlled with non-targeted siRNA [siNT]). Control cells were exposed non-targetting (siNT). Representative western blots to demonstrate the efficiency of the knockdown are shown. After transfection, cells were incubated for 96 hours before being treated with IL-1β (1ng/mL) progesterone (10μM) and forskolin (100μM) either alone or in combination for 6 hours. The mRNA was extracted, and the levels of OTR mRNA were measured using rt-PCR. Data are shown as the mean and SEM, and were compared (IL-1β vs. IL-1β and other treatment combinations) using Friedman’s Test, with a Dunn's Multiple Comparisons post hoc test for data that were not normally distributed, and using ANOVA, with Dunnett and Bonferroni’s post-test for data that were normally distributed. *P<0.05, **P<0.01, ***P<0.001 (n = 6–7 myometrial cells from 6–7 different women).
(PPTX)

S9 Fig The effects of a PKA antagonist on the ability of the combination of progesterone and forskolin to reduce the IL-1β-induced increase in COX-2, OTR and IL-8 mRNA expression. Myometrial cells were isolated from myometrial biopsies obtained from women at the time of pre-labor term Caesarean section as described above in *Materials and Methods*. After the cells were about 80% confluent, the cells were treated with PKA antagonist (KT5720 10μM, PKA inhibitor) for 6 hours, followed by progesterone (10μM), IL-1β (1ng/mL) and/or forskolin (100μM) either alone or in combination for 6 hours. The mRNA was extracted, and the levels of OTR mRNA were measured using rt-PCR. Data are shown as the mean and SEM, and were compared using (IL-1β vs. IL-1β and other treatment combinations) using Friedman’s Test, with a Dunn's Multiple Comparisons post hoc test for data that were not normally distributed, and using ANOVA, with Dunnett and Bonferroni’s post-test for data that were normally distributed.*P<0.05, **P<0.01, ***P<0.001 (n = 6–7 myometrial cells from 6–7 different women).
(PPTX)

S1 File (DOCX)

S1 Raw images (PDF)

S1 Data (XLSX)
