# Supplementary material for: The interaction between protein kinase A and progesterone on basal and inflammation-induced myometrial oxytocin receptor expression
Source: PLoS One. 2020 Dec 1;15(12):e0239937. doi: 10.1371/journal.pone.0239937 (PMC7707466; doi:10.1371/journal.pone.0239937)
Supplement: S1 Fig — Myometrial cells were isolated as described above in Materials and Methods, and treated with IL-1β (1ng/ml) alone or in combination with forskolin (100μM) and/or progesterone (0.1, 1.0 or 10μM) for 6 hours. mRNA was extracted, and the levels of OTR mRNA measured using rt-PCR. Data are shown as the mean and SEM, and were compared using Wilcoxon matched pairs test for data that were not normally distributed and paired t test for data that were normally distributed. *P<0.05, **P<0.01 (n = 6–9 myometrial samples from 6–9 different women in each experiment). (PPTX) [file pone.0239937.s001.pptx]

## Slide 1
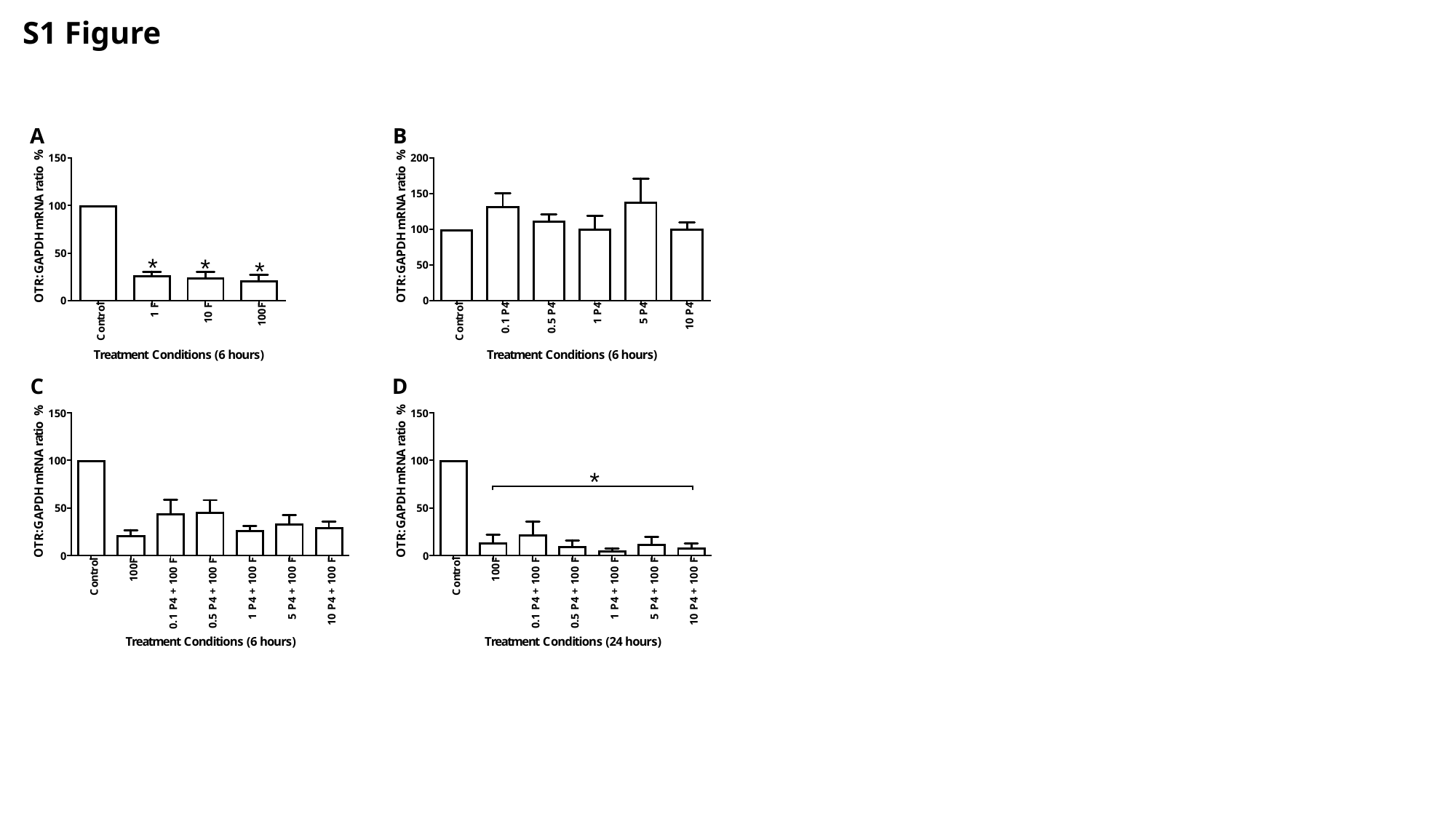

S1 Figure
A
B
%
%
150
100
50
*
0
l
F
F
F
o
0
r
1
0
0
t
1
n
1
o
C
Tr
e
a
t
m
e
n
t
C
o
n
d
i
t
i
o
n
s
%
150
o
i
t
a
r
A
N
100
R
*
m
H
D
P
50
A
G
:
R
T
O
0
l
F
F
F
F
F
F
0
0
0
0
0
0
0
0
0
0
0
0
1
1
1
1
1
1
+
+
+
+
+
4
4
4
4
4
P
P
P
P
P
1
5
1
5
0
.
.
1
0
0
Tr
e
a
t
m
e
n
t
C
o
n
d
i
t
i
o
n
s
(
2
4
h
o
u
r
s
)
200
o
o
i
i
t
t
a
a
r
r
150
A
A
N
N
R
R
m
m
100
H
H
D
D
P
P
*
A
A
*
50
G
G
:
:
R
R
T
T
O
O
0
l
4
4
4
4
4
o
P
P
P
P
P
r
t
1
5
1
5
0
n
.
.
1
o
0
0
C
(
6
h
o
u
r
s
)
Tr
e
a
t
m
e
n
t
C
o
n
d
i
t
i
o
n
s
(
6
h
o
u
r
s
)
C
D
%
150
o
i
t
a
r
A
N
100
R
m
H
D
P
50
A
G
:
R
T
O
0
l
F
F
F
F
F
F
o
o
0
r
r
0
0
0
0
0
0
t
t
0
0
0
0
0
n
n
1
1
1
1
1
1
o
o
+
+
+
+
+
C
C
4
4
4
4
4
P
P
P
P
P
5
1
5
0
1
.
.
1
0
0
Tr
e
a
t
m
e
n
t
C
o
n
d
i
t
i
o
n
s
(
6
h
o
u
r
s
)
