# Supplementary material for: The interaction between protein kinase A and progesterone on basal and inflammation-induced myometrial oxytocin receptor expression
Source: PLoS One. 2020 Dec 1;15(12):e0239937. doi: 10.1371/journal.pone.0239937 (PMC7707466; doi:10.1371/journal.pone.0239937)
Supplement: S2 Fig — Myometrial cells were isolated from myometrial biopsies obtained from women at the time of pre-labor term Caesarean section as described above in Materials and Methods. After the cells were about 80% confluent cells were treated with progesterone (10μM) and/or forskolin (100μM) either alone or in combination for 6, 24 or 48 hours. The mRNA was extracted, and the mRNA levels of down-regulated genes: GUCY1A3, GPR124, CREB3L1, OTR and PRKG1 (A-E) and up-regulated genes: CCL8, 11βHSD1, MKP-1, PDE4B, PTGES and GPR125 (F-K) measured using rt-PCR. Data were compared using Friedman’s Test, with a Dunn's Multiple Comparisons post hoc test for data that were not normally distributed, and using ANOVA, with Dunnett and Bonferroni’s post-test for data that were normally distributed, *P<0.05, **P<0.01 (n = 6–7 myometrial cells from 6–7 different women). (PPTX) [file pone.0239937.s002.pptx]

## Slide 1
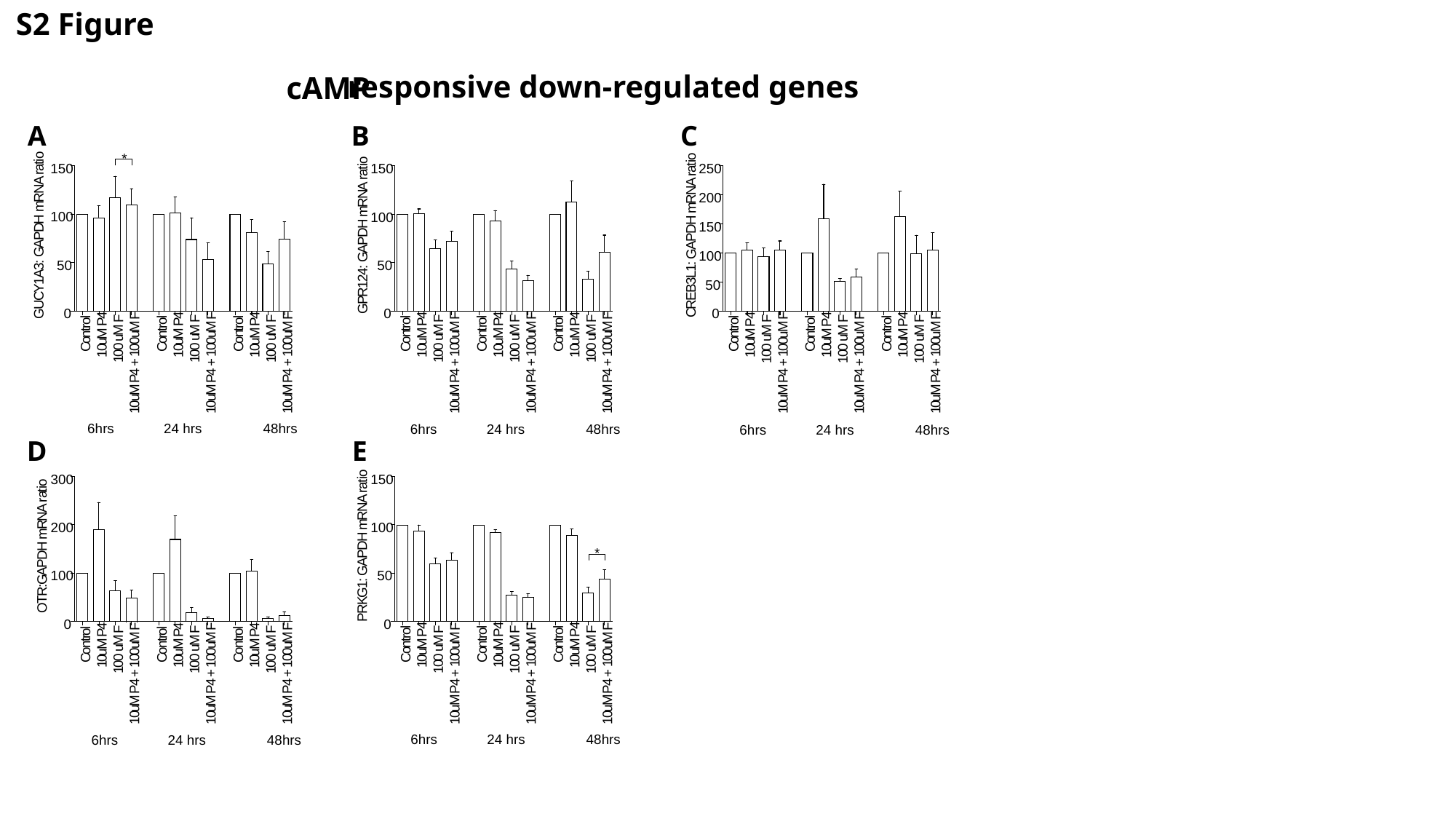

S2 Figure
 responsive down-regulated genes
cAMP
A
B
C
o
*
i
t
150
a
100
H
D
P
A
G
50
:
4
2
1
R
P
G
0
4
4
4
F
F
F
l
l
l
F
F
F
o
o
o
P
P
P
r
r
r
M
M
M
t
t
t
M
M
M
M
M
M
n
n
n
u
u
u
u
u
u
o
o
o
0
0
0
u
u
u
0
0
0
0
0
0
C
C
C
0
0
0
0
1
0
1
0
1
1
1
1
1
1
1
+
+
+
4
4
4
P
P
P
M
M
M
u
u
u
0
0
0
1
1
1
6hrs 24 hrs 48hrs
o
i
t
a
r
A
N
R
m
H
D
P
A
G
:
6hrs 24 hrs 48hrs
300
200
100
R
T
O
0
4
4
4
F
F
F
l
l
l
F
F
F
o
o
o
P
P
P
r
r
r
M
M
M
t
t
t
M
M
M
M
M
M
n
n
n
u
u
u
u
u
u
o
o
o
0
0
0
u
u
u
0
0
0
0
0
0
C
C
C
0
0
0
0
1
0
1
0
1
1
1
1
1
1
1
+
+
+
4
4
4
P
P
P
M
M
M
u
u
u
0
0
0
1
1
1
o
i
250
t
a
r
A
N
200
R
m
150
100
1
L
3
50
B
E
R
0
C
4
4
4
F
F
F
l
l
l
F
F
F
o
o
o
P
P
P
r
r
r
M
M
M
t
t
t
M
M
M
M
M
M
n
n
n
u
u
u
u
u
u
o
o
o
0
0
0
u
u
u
0
0
0
0
0
0
C
C
C
0
0
0
0
1
0
1
0
1
1
1
1
1
1
1
+
+
+
4
4
4
P
P
P
M
M
M
u
u
u
0
0
0
1
1
1
6hrs 24 hrs 48hrs
150
r
A
N
R
m
100
H
D
P
A
G
:
50
3
A
1
Y
C
U
0
G
4
4
4
F
F
F
l
l
l
F
F
F
o
o
o
P
P
P
r
r
r
M
M
M
t
t
t
M
M
M
M
M
M
n
n
n
u
u
u
u
u
u
o
o
o
0
0
0
u
u
u
0
0
0
0
0
0
C
C
C
0
0
0
0
1
0
1
0
1
1
1
1
1
1
1
+
+
+
4
4
4
P
P
P
M
M
M
u
u
u
0
0
0
1
1
1
D
E
o
150
o
i
t
a
r
A
N
R
100
m
H
D
P
A
50
G
:
K
R
P
0
4
4
4
F
F
F
l
l
l
F
F
F
o
o
o
P
P
P
r
r
r
M
M
M
t
t
t
M
M
M
M
M
M
n
n
n
u
u
u
u
u
u
o
o
o
0
0
0
u
u
u
0
0
0
0
0
0
C
C
C
0
0
0
0
1
0
1
0
1
1
1
1
1
1
1
+
+
+
4
4
4
P
P
P
M
M
M
u
u
u
0
0
0
1
1
1
6hrs 24 hrs 48hrs
i
t
a
r
A
N
R
m
H
D
*
P
A
G
:
1
G
6hrs 24 hrs 48hrs

## Slide 2
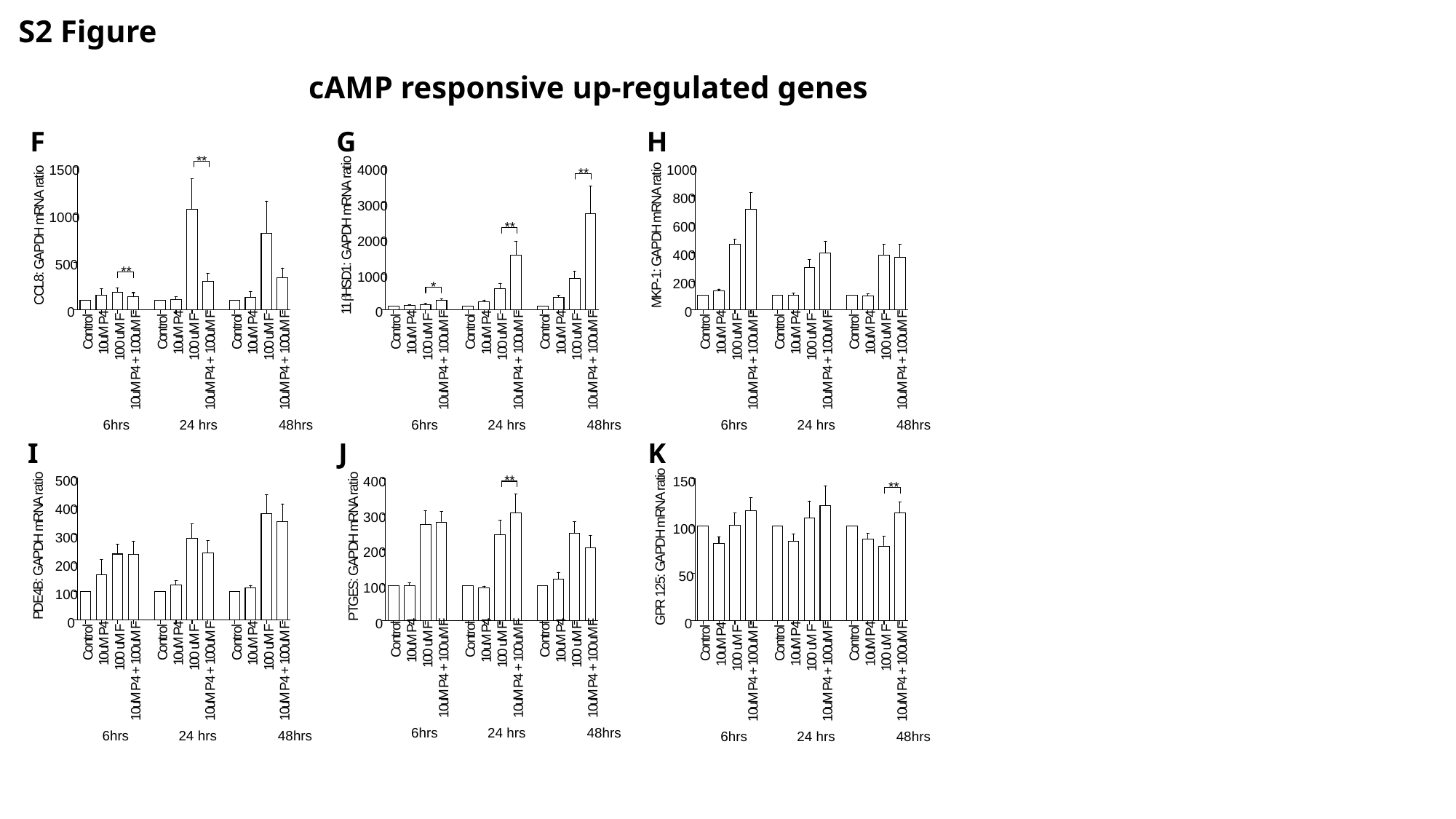

S2 Figure
cAMP responsive up-regulated genes
F
G
H
**
o
4000
3000
P
2000
A
G
:
1
**
D
1000
S
H
b
1
1
0
4
4
4
F
F
F
l
l
l
F
F
F
o
o
o
P
P
P
r
r
r
M
M
M
t
t
t
M
M
M
M
M
M
n
n
n
u
u
u
u
u
u
o
o
o
0
0
0
u
u
u
0
0
0
0
0
0
C
C
C
0
0
0
0
1
0
1
0
1
1
1
1
1
1
1
+
+
+
4
4
4
P
P
P
M
M
M
u
u
u
0
0
0
1
1
1
6hrs 24 hrs 48hrs
o
i
t
a
**
r
A
N
R
m
H
**
D
*
0
4
4
4
F
F
F
l
l
l
F
F
F
o
o
o
P
P
P
r
r
r
M
M
M
t
t
t
M
M
M
M
M
M
n
n
n
u
u
u
u
u
u
o
o
o
0
0
0
u
u
u
0
0
0
0
0
0
C
C
C
0
0
0
0
1
0
1
0
1
1
1
1
1
1
1
+
+
+
4
4
4
P
P
P
M
M
M
u
u
u
0
0
0
1
1
1
6hrs 24 hrs 48hrs
1500
i
t
a
r
A
N
R
1000
m
H
D
P
A
G
500
:
8
L
C
C
0
4
4
4
F
F
F
l
l
l
F
F
F
o
o
o
P
P
P
r
r
r
M
M
M
t
t
t
M
M
M
M
M
M
n
n
n
u
u
u
u
u
u
o
o
o
0
0
0
u
u
u
0
0
0
0
0
0
C
C
C
0
0
0
0
1
0
1
0
1
1
1
1
1
1
1
+
+
+
4
4
4
P
P
P
M
M
M
u
u
u
0
0
0
1
1
1
o
1000
i
t
a
r
A
800
N
R
m
600
H
D
P
A
400
G
:
1
-
200
P
K
M
6hrs 24 hrs 48hrs
4
4
4
F
F
F
l
l
l
F
F
F
o
o
o
P
P
P
r
r
r
M
M
M
t
t
t
M
M
M
M
M
M
n
n
n
u
u
u
u
u
u
o
o
o
0
0
0
u
u
u
0
0
0
0
0
0
C
C
C
0
0
0
0
1
0
1
0
1
1
1
1
1
1
1
+
+
+
4
4
4
P
P
P
M
M
M
u
u
u
0
0
0
1
1
1
I
J
K
o
i
t
o
**
i
400
t
a
r
A
N
300
R
m
H
D
200
P
A
G
:
S
100
E
G
T
P
4
4
4
F
F
F
l
l
l
F
F
F
o
o
o
P
P
P
r
r
r
M
M
M
t
t
t
M
M
M
M
M
M
n
n
n
u
u
u
u
u
u
o
o
o
0
0
0
u
u
u
0
0
0
0
0
0
C
C
C
0
0
0
0
1
0
1
0
1
1
1
1
1
1
1
+
+
+
4
4
4
P
P
P
M
M
M
u
u
u
0
0
0
1
1
1
6hrs 24 hrs 48hrs
o
i
500
t
a
r
A
N
400
R
m
300
H
D
P
A
200
G
:
B
4
100
E
D
P
4
4
4
F
F
F
0
l
l
l
0
F
F
F
o
o
o
P
P
P
r
r
r
M
M
M
t
t
t
M
M
M
M
M
M
n
n
n
u
u
u
u
u
u
o
o
o
0
0
0
u
u
u
0
0
0
0
0
0
C
C
C
0
0
0
0
1
0
1
0
1
1
1
1
1
1
1
+
+
+
4
4
4
P
P
P
M
M
M
u
u
u
0
0
0
1
1
1
6hrs 24 hrs 48hrs
150
a
**
r
A
N
R
m
100
H
D
P
A
G
50
:
5
2
1
R
P
G
0
6hrs 24 hrs 48hrs
