# Supplementary material for: The interaction between protein kinase A and progesterone on basal and inflammation-induced myometrial oxytocin receptor expression
Source: PLoS One. 2020 Dec 1;15(12):e0239937. doi: 10.1371/journal.pone.0239937 (PMC7707466; doi:10.1371/journal.pone.0239937)
Supplement: S3 Fig — (Fig 3 data are included for comparison) Myometrial cells were isolated from myometrial biopsies obtained from women at the time of pre-labor term Caesarean section as described above in Materials and Methods. After the cells were about 80% confluent, cAMP effectors such as PKA, Epac1 and AMPK were knocked down using siRNA (siPKAC-α, siEpac1, siAMPK controlled with non-targeted siRNA [siNT]). Representative western blots to demonstrate the efficacy of knockdown are shown. After transfection, cells were incubated for 96 hours before being treated with IL-1β (1ng/mL) and/or forskolin (100μM) either alone or in combination for 6 hours. The mRNA was extracted, and the levels of OTR mRNA were measured using rt-PCR. The data are expressed as mean SEM and compared using Wilcoxon matched pairs test for data that were not normally distributed and paired t test for data that were normally distributed to compare control vs. forskolin alone and IL-1β alone vs. IL-1β and forskolin. *P<0.05, **P<0.01, ***P<0.001 (n = 6–7 myometrial cells from 6–7 different women). (PPTX) [file pone.0239937.s003.pptx]

## Slide 1
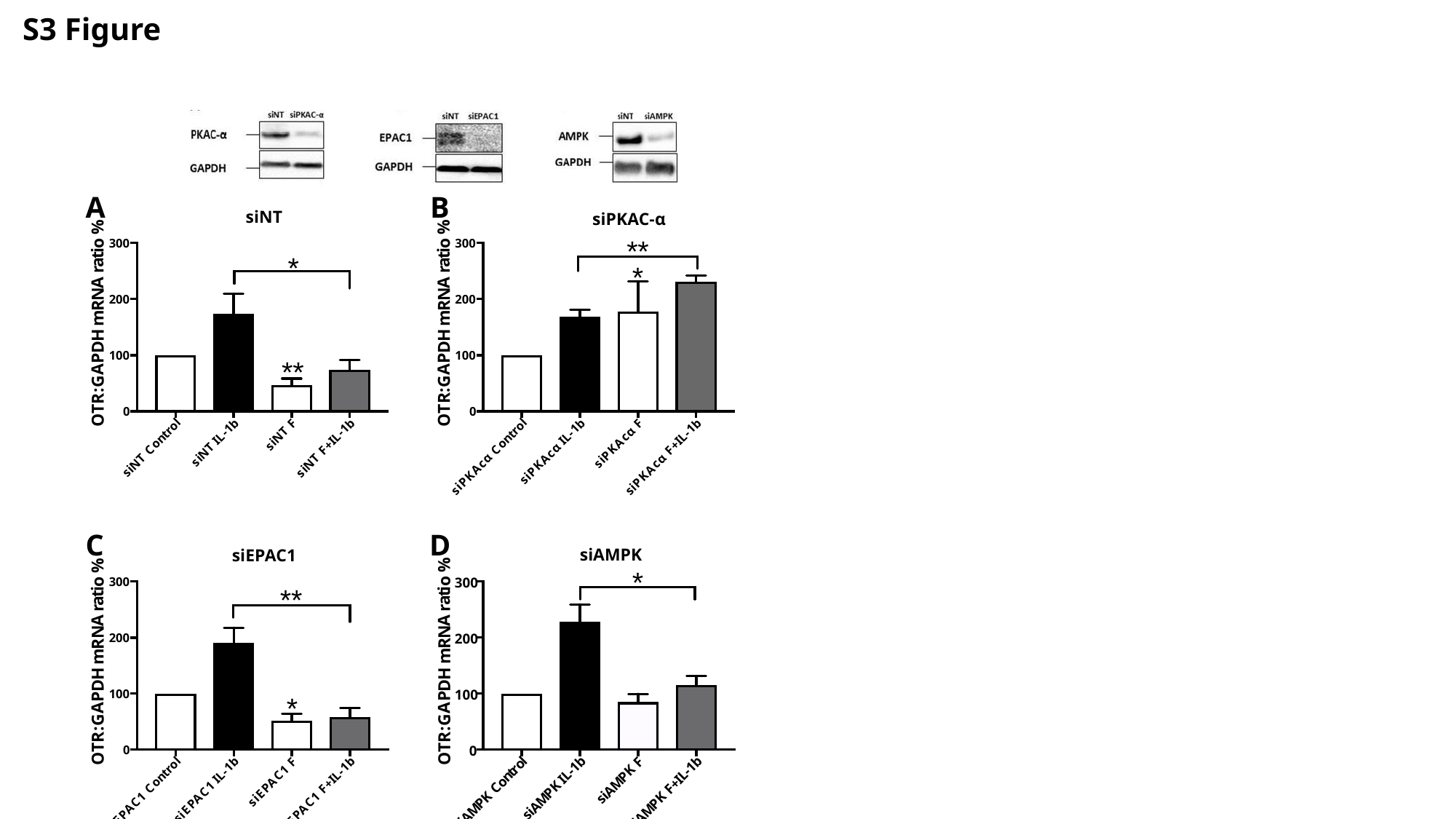

S3 Figure
A
B
siNT
%
o
300
i
t
*
a
r
A
N
200
R
m
H
D
P
100
**
A
G
:
R
T
0
O
l
b
F
b
o
1
1
r
T
-
-
t
L
L
N
n
I
I
i
o
+
s
T
C
F
N
T
i
T
s
N
N
i
i
s
s
siEP
AC1
%
o
300
i
**
t
a
r
A
N
200
R
m
H
D
100
P
*
A
G
:
R
T
0
O
l
b
F
b
o
1
1
r
1
-
-
t
C
L
L
n
I
I
o
A
+
1
P
C
F
C
E
1
1
i
A
s
C
C
P
A
A
E
i
P
P
s
E
E
i
i
s
s
%
**
o
300
i
t
a
*
r
A
N
200
R
m
H
D
P
100
A
G
:
R
T
0
O
l
b
F
b
o
1
1
r
α
-
-
t
c
L
L
n
I
I
A
o
+
α
K
C
F
c
P
α
α
A
i
c
s
c
K
A
A
P
K
i
K
s
P
P
i
i
s
s
siAMPK
%
*
o
300
i
t
a
r
A
N
200
R
m
H
D
P
100
A
G
:
R
T
0
O
l
b
F
b
o
1
1
r
K
-
-
t
L
L
n
P
I
I
o
M
+
K
C
F
A
P
i
K
K
s
M
P
P
A
M
M
i
s
A
A
i
i
s
s
C
D
siPKAC-α
