# Supplementary figures and images for: The interaction between protein kinase A and progesterone on basal and inflammation-induced myometrial oxytocin receptor expression
Source: PLoS One. 2020 Dec 1;15(12):e0239937. doi: 10.1371/journal.pone.0239937 (PMC7707466; doi:10.1371/journal.pone.0239937)

## Slide 1
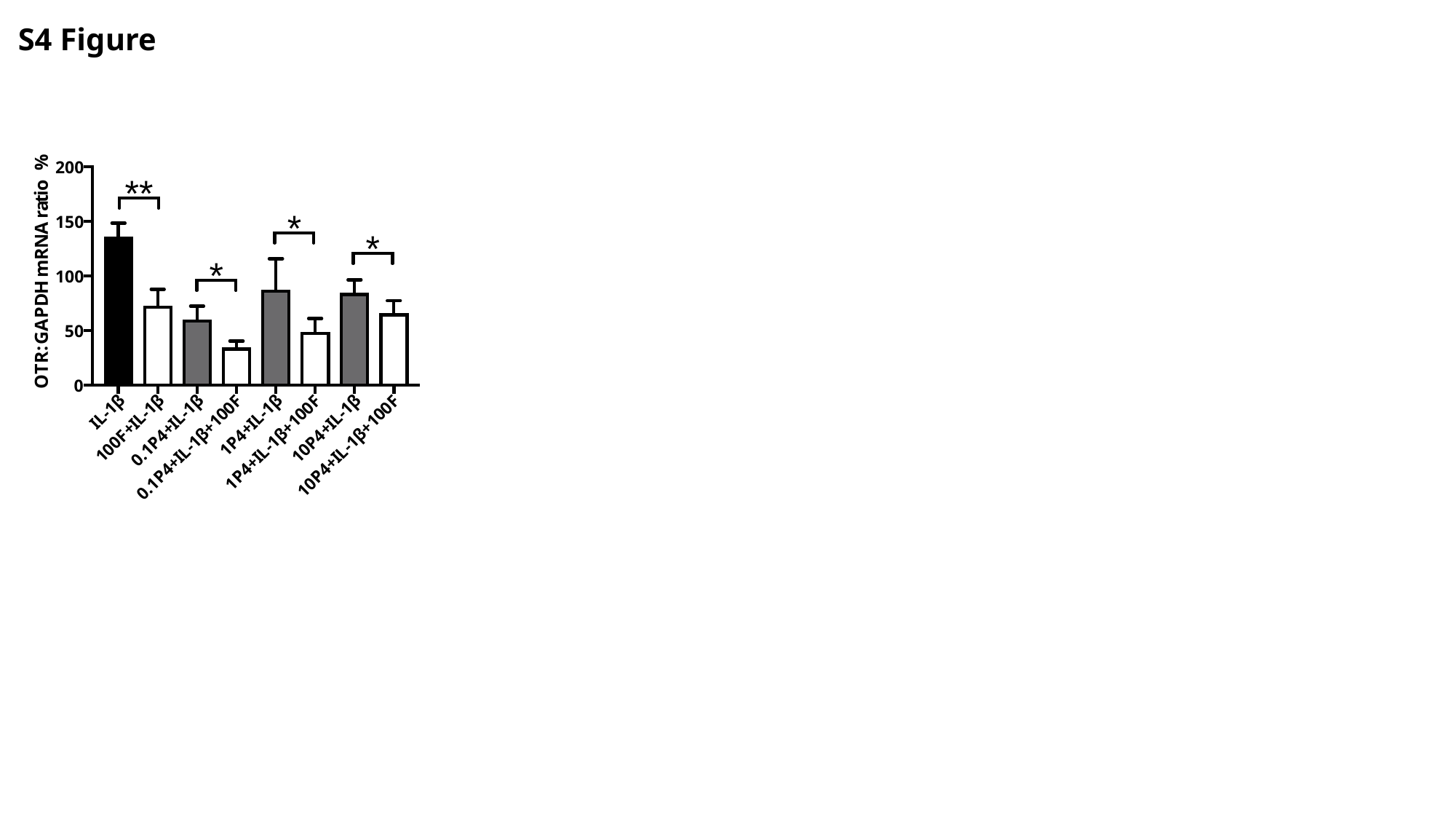

S4 Figure
%
200
**
o
i
t
a
r
*
150
A
*
N
R
*
m
100
H
D
P
A
50
G
:
R
T
O
0
β
β
β
F
β
F
β
F
1
1
1
0
1
0
1
0
-
-
-
-
-
0
0
0
L
L
L
L
L
1
1
1
I
I
I
I
I
+
+
+
+
+
+
+
β
β
β
4
4
4
F
1
1
1
0
P
P
P
-
-
-
0
1
1
0
L
L
L
.
1
1
I
I
I
0
+
+
+
4
4
4
P
P
P
1
1
0
.
1
0

Supplement: S4 Fig — Myometrial cells were isolated as described in Materials and Methods, and treated with different doses of progesterone (0.1 μM, 1 μM, 10μM), forskolin (100μM) and IL-1β (1ng/mL) either alone or in combination for 6 hours. mRNA was extracted, and the levels of OTR mRNA were measured using quantitative rt-PCR. Data are expressed as mean SEM and were compared using Friedman’s Test, with a Dunn's Multiple Comparisons post hoc test for data that were not normally distributed, and using ANOVA, with Dunnett and Bonferroni’s post-test for data that were normally distributed, *P<0.05, **P<0.01 (n = 8–9 myometrial cells from 8–9 different women). (PPTX) [file pone.0239937.s004.pptx]
