# Supplementary material for: The interaction between protein kinase A and progesterone on basal and inflammation-induced myometrial oxytocin receptor expression
Source: PLoS One. 2020 Dec 1;15(12):e0239937. doi: 10.1371/journal.pone.0239937 (PMC7707466; doi:10.1371/journal.pone.0239937)
Supplement: S5 Fig — Myometrial cells were isolated as described above in Materials and Methods, and treated with (i) IL-1β (1ng/ml) alone or in combination with forskolin (100μM) and/or progesterone (10μM) or with (ii) IL-1β (1ng/ml) alone or in combination with sp-6-phe-cAMP (100nM) and/or progesterone (10μM) for 6 hours. mRNA was extracted, and the levels of OTR mRNA measured using rt-PCR. Data are shown as the mean and SEM, and were compared (IL-1β vs. IL-1β and other treatment combinations) using Friedman’s Test, with a Dunn's Multiple Comparisons post hoc test for data that were not normally distributed, and using ANOVA, with Dunnett and Bonferroni’s post-test for data that were normally distributed. *P<0.05, **P<0.01 (n = 6–9 myometrial samples from 6–9 different women in each experiment). (PPTX) [file pone.0239937.s005.pptx]

## Slide 1
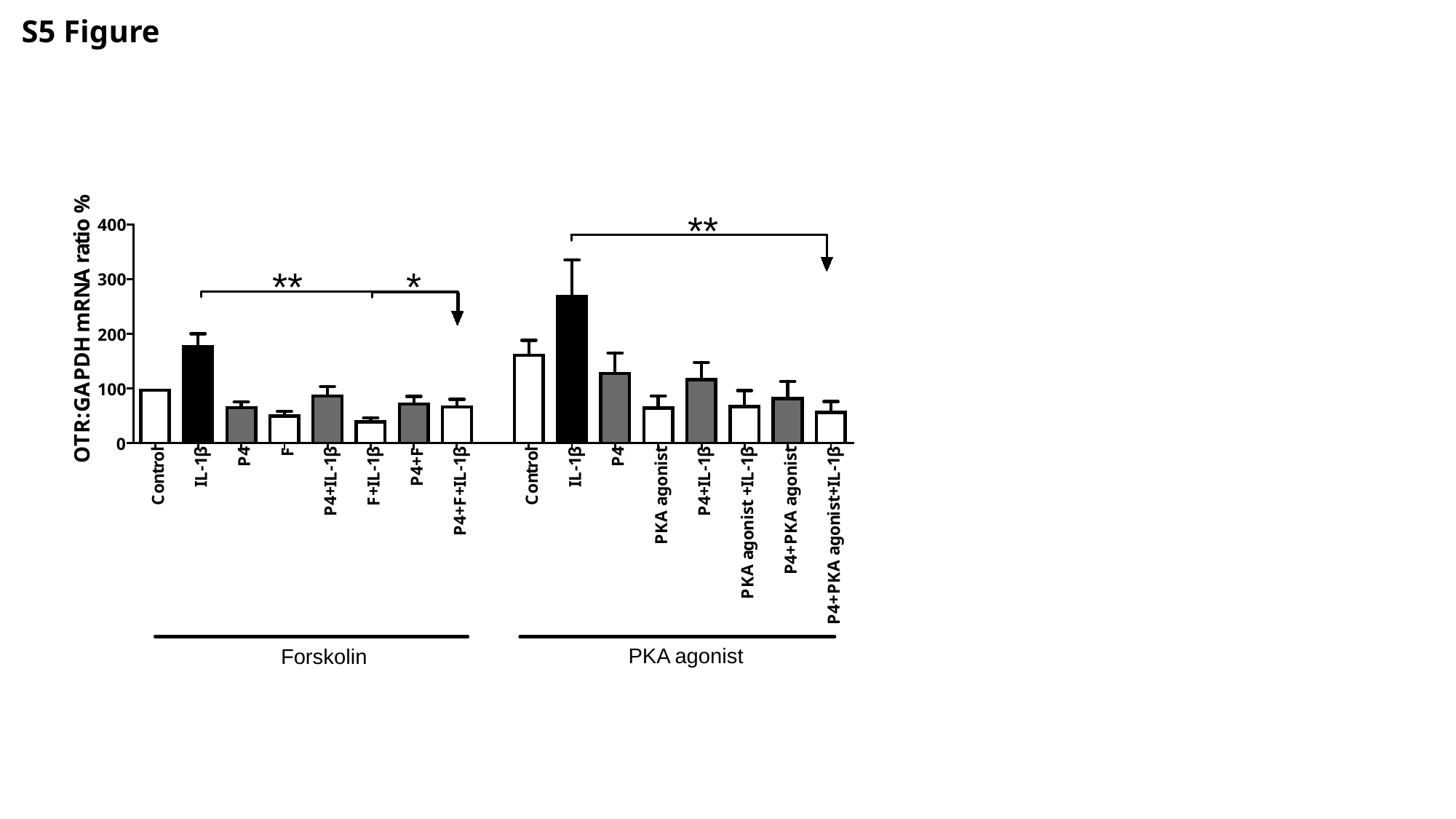

S5 Figure
%
**
o
400
i
l
l
t
t
4
4
F
β
β
β
β
β
F
β
β
β
o
o
s
s
1
1
P
1
1
1
P
1
1
+
1
i
i
r
r
-
-
-
-
-
-
-
-
t
t
4
n
n
L
L
L
L
L
L
L
L
n
n
P
o
o
I
I
I
I
I
I
I
I
o
o
+
+
+
+
+
g
+
g
t
C
C
4
4
a
F
F
a
t
s
P
s
P
+
i
A
A
i
4
n
n
K
K
P
o
o
P
P
g
g
+
a
a
4
A
P
A
K
K
P
P
+
4
P
t
a
r
**
*
A
300
N
R
m
200
H
D
P
A
100
G
:
R
T
0
O
PKA
 agonist
Forskolin
