# Supplementary material for: The interaction between protein kinase A and progesterone on basal and inflammation-induced myometrial oxytocin receptor expression
Source: PLoS One. 2020 Dec 1;15(12):e0239937. doi: 10.1371/journal.pone.0239937 (PMC7707466; doi:10.1371/journal.pone.0239937)
Supplement: S6 Fig — Myometrial cells were isolated from myometrial biopsies obtained from women at the time of pre-labor term Caesarean section as described above in Materials and Methods, and treated with progesterone (10μM), forskolin (100μM) or IL-1β (1ng/mL) either alone or in combination for for 30 min. Cells were lysed and protein extracted. Western blotting was performed using antibodies directed against phospho-JNK (A), and phospho p38 (B). α-tubulin was used as the internal control. Data were compared (IL-1β vs. IL-1β and other treatment combinations) using Friedman’s Test, with a Dunn's Multiple Comparisons post hoc test for data that were not normally distributed, and using ANOVA, with Dunnett and Bonferroni’s post-test for data that were normally distributed, P<0.05, n = 6 myometrial cells from 6 different women. *P<0.05 vs. IL-1β. (PPTX) [file pone.0239937.s006.pptx]

## Slide 1
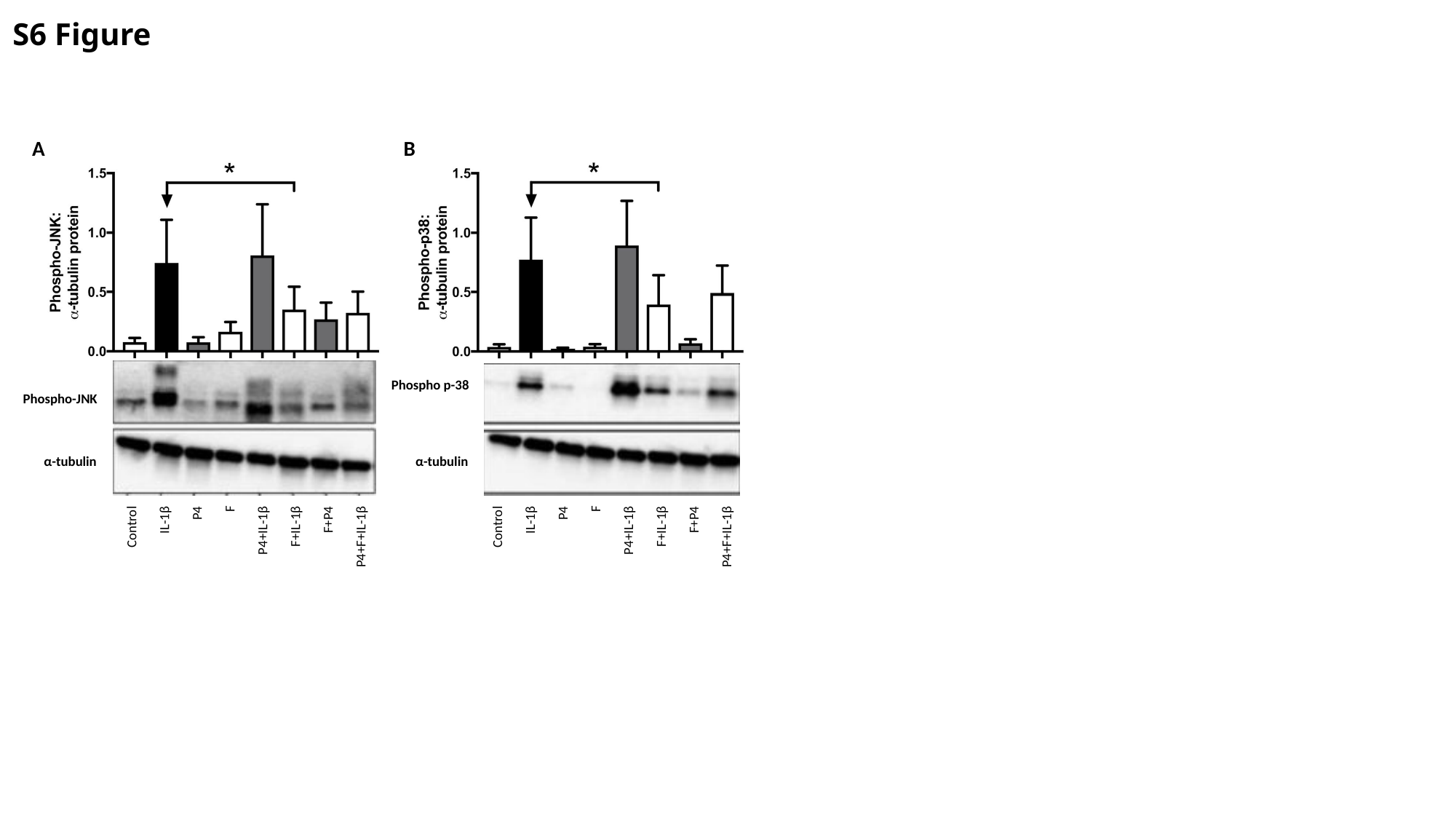

S6 Figure
A
B
Phospho p-38
Phospho-JNK
Control
IL-1β
P4
F
P4+IL-1β
F+IL-1β
F+P4
P4+F+IL-1β
Control
IL-1β
P4
F
P4+IL-1β
F+IL-1β
F+P4
P4+F+IL-1β
α-tubulin
α-tubulin
