# Supplementary material for: The interaction between protein kinase A and progesterone on basal and inflammation-induced myometrial oxytocin receptor expression
Source: PLoS One. 2020 Dec 1;15(12):e0239937. doi: 10.1371/journal.pone.0239937 (PMC7707466; doi:10.1371/journal.pone.0239937)
Supplement: S7 Fig — Myometrial cells were isolated from myometrial biopsies obtained from women at the time of pre-labor term Caesarean section as described above in Materials and Methods, and treated with progesterone (10μM), forskolin (100μM) or IL-1β (1ng/mL) either alone or in combination for for 0 min, 30 min, 1 h, 2 h and 6 h. Cells were lysed and samples purified for cytoplasmic protein. Western blotting was performed using antibodies directed against MKP-1 (A) and IKBα (B). α-tubulin was used as the internal controls for the cytosolic fraction. MKP-1 data were compared across all groups and for the IKB data, all groups treated with IL-1β were compared using Friedman’s Test, with a Dunn's Multiple Comparisons post hoc test for data that were not normally distributed, and using ANOVA, with Dunnett and Bonferroni’s post-test for data that were normally distributed, P<0.05, **P<0.01, ***P<0.001; n = 6 myometrial cells from 6 different women and only 30 minutes, 1 hour and 2 hours time points are shown). (PPTX) [file pone.0239937.s007.pptx]

## Slide 1
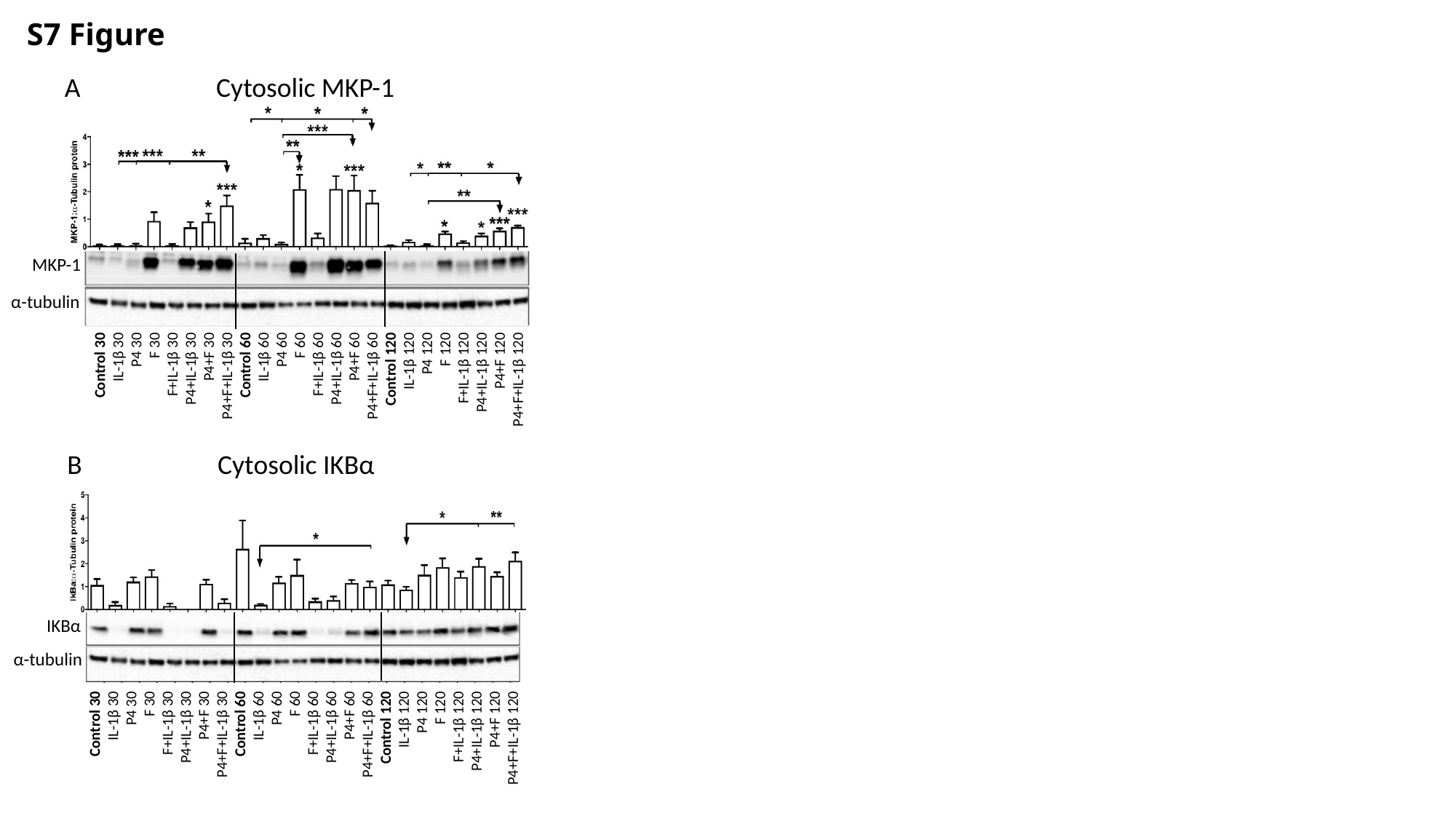

S7 Figure
A Cytosolic MKP-1
Control 30
IL-1β 30
P4 30
F 30
F+IL-1β 30
P4+IL-1β 30
P4+F 30
P4+F+IL-1β 30
Control 60
IL-1β 60
P4 60
F 60
F+IL-1β 60
P4+IL-1β 60
P4+F 60
P4+F+IL-1β 60
Control 120
IL-1β 120
P4 120
F 120
F+IL-1β 120
P4+IL-1β 120
P4+F 120
P4+F+IL-1β 120
MKP-1
α-tubulin
B Cytosolic IKBα
Control 30
IL-1β 30
P4 30
F 30
F+IL-1β 30
P4+IL-1β 30
P4+F 30
P4+F+IL-1β 30
Control 60
IL-1β 60
P4 60
F 60
F+IL-1β 60
P4+IL-1β 60
P4+F 60
P4+F+IL-1β 60
Control 120
IL-1β 120
P4 120
F 120
F+IL-1β 120
P4+IL-1β 120
P4+F 120
P4+F+IL-1β 120
IKBα
α-tubulin
