# Supplementary material for: The interaction between protein kinase A and progesterone on basal and inflammation-induced myometrial oxytocin receptor expression
Source: PLoS One. 2020 Dec 1;15(12):e0239937. doi: 10.1371/journal.pone.0239937 (PMC7707466; doi:10.1371/journal.pone.0239937)
Supplement: S8 Fig — Myometrial cells were isolated from myometrial biopsies obtained from women at the time of pre-labor term Caesarean section as described above in Materials and Methods. After the cells were about 80% confluent, cAMP effectors including PKA, Epac1 and AMPK were knocked down using siRNA (siPKA, siEpac1, siAMPK controlled with non-targeted siRNA [siNT]). Control cells were exposed non-targetting (siNT). Representative western blots to demonstrate the efficiency of the knockdown are shown. After transfection, cells were incubated for 96 hours before being treated with IL-1β (1ng/mL) progesterone (10μM) and forskolin (100μM) either alone or in combination for 6 hours. The mRNA was extracted, and the levels of OTR mRNA were measured using rt-PCR. Data are shown as the mean and SEM, and were compared (IL-1β vs. IL-1β and other treatment combinations) using Friedman’s Test, with a Dunn's Multiple Comparisons post hoc test for data that were not normally distributed, and using ANOVA, with Dunnett and Bonferroni’s post-test for data that were normally distributed. *P<0.05, **P<0.01, ***P<0.001 (n = 6–7 myometrial cells from 6–7 different women). (PPTX) [file pone.0239937.s008.pptx]

## Slide 1
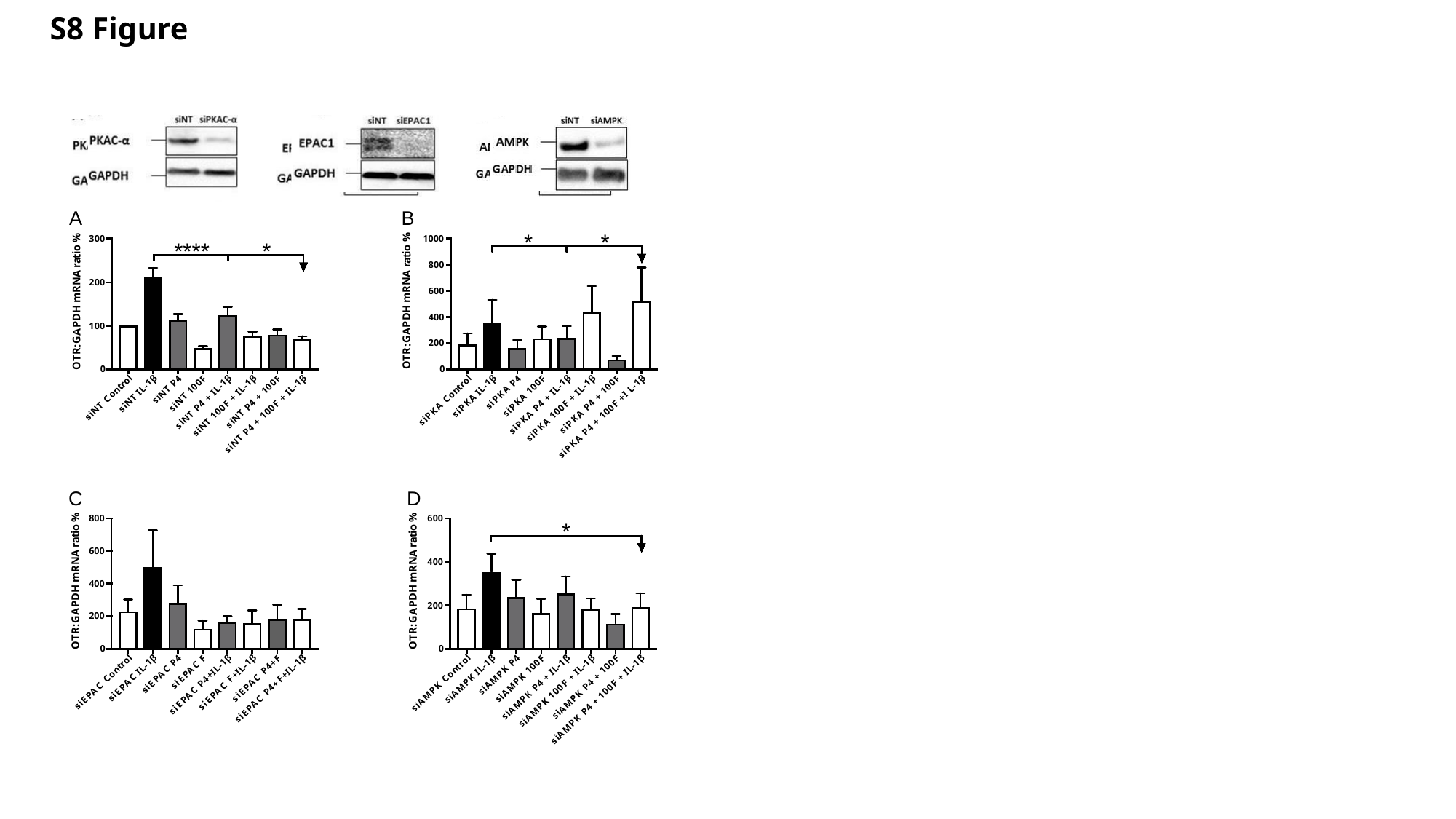

S8 Figure
A
%
300
****
*
o
i
t
a
r
A
N
200
R
m
H
D
P
100
A
G
:
R
T
O
0
l
4
β
F
β
β
F
β
o
1
P
0
1
1
0
1
r
-
-
-
-
0
0
t
L
T
L
L
L
n
1
1
I
I
I
I
N
o
T
+
T
i
+
+
+
C
s
N
N
4
4
F
F
i
T
i
P
0
0
P
s
s
N
0
0
T
i
T
1
1
s
N
N
T
i
+
i
s
s
N
4
i
P
s
T
N
i
s
B
*
*
%
1000
o
i
t
a
r
800
A
N
R
600
m
H
D
400
P
A
G
200
:
R
T
O
0
4
F
β
β
F
β
0
1
1
0
1
-
-
-
0
0
L
L
L
1
1
I
I
I
+
A
+
+
+
K
4
4
F
F
P
P
P
0
0
i
0
0
s
A
A
1
1
K
K
+
A
P
P
K
i
i
4
s
s
P
P
i
s
A
K
P
i
s
l
4
β
o
1
P
r
-
t
L
K
n
I
o
P
K
K
C
M
P
P
A
K
M
M
i
P
s
A
A
M
i
i
s
s
A
i
s
l
β
o
1
P
r
-
t
L
A
n
I
o
K
A
C
P
K
i
s
A
P
K
i
s
P
i
s
C
%
800
o
i
t
a
r
600
A
N
R
m
400
H
D
P
A
200
G
:
R
T
O
0
l
4
β
F
β
β
F
β
o
1
P
1
1
1
+
r
C
-
-
-
-
t
4
L
L
L
L
C
n
A
P
I
I
I
I
o
P
A
+
+
+
C
C
C
P
E
4
F
F
i
A
A
P
E
+
s
C
C
i
P
P
4
s
C
A
A
E
E
P
i
P
i
A
P
s
s
C
P
E
E
i
i
A
E
s
s
i
P
s
E
i
s
D
%
600
*
o
i
t
a
r
A
N
400
R
m
H
D
P
200
A
G
:
R
T
O
0
F
β
β
F
β
0
1
1
0
1
-
-
-
0
0
L
L
L
1
1
I
I
I
+
+
+
+
4
4
F
F
P
P
0
0
0
0
K
K
1
1
P
P
+
K
M
M
P
4
A
A
P
M
i
i
s
s
A
K
i
P
s
M
A
i
s
