# Supplementary material for: The interaction between protein kinase A and progesterone on basal and inflammation-induced myometrial oxytocin receptor expression
Source: PLoS One. 2020 Dec 1;15(12):e0239937. doi: 10.1371/journal.pone.0239937 (PMC7707466; doi:10.1371/journal.pone.0239937)
Supplement: S9 Fig — Myometrial cells were isolated from myometrial biopsies obtained from women at the time of pre-labor term Caesarean section as described above in Materials and Methods. After the cells were about 80% confluent, the cells were treated with PKA antagonist (KT5720 10μM, PKA inhibitor) for 6 hours, followed by progesterone (10μM), IL-1β (1ng/mL) and/or forskolin (100μM) either alone or in combination for 6 hours. The mRNA was extracted, and the levels of OTR mRNA were measured using rt-PCR. Data are shown as the mean and SEM, and were compared using (IL-1β vs. IL-1β and other treatment combinations) using Friedman’s Test, with a Dunn's Multiple Comparisons post hoc test for data that were not normally distributed, and using ANOVA, with Dunnett and Bonferroni’s post-test for data that were normally distributed.*P<0.05, **P<0.01, ***P<0.001 (n = 6–7 myometrial cells from 6–7 different women). (PPTX) [file pone.0239937.s009.pptx]

## Slide 1
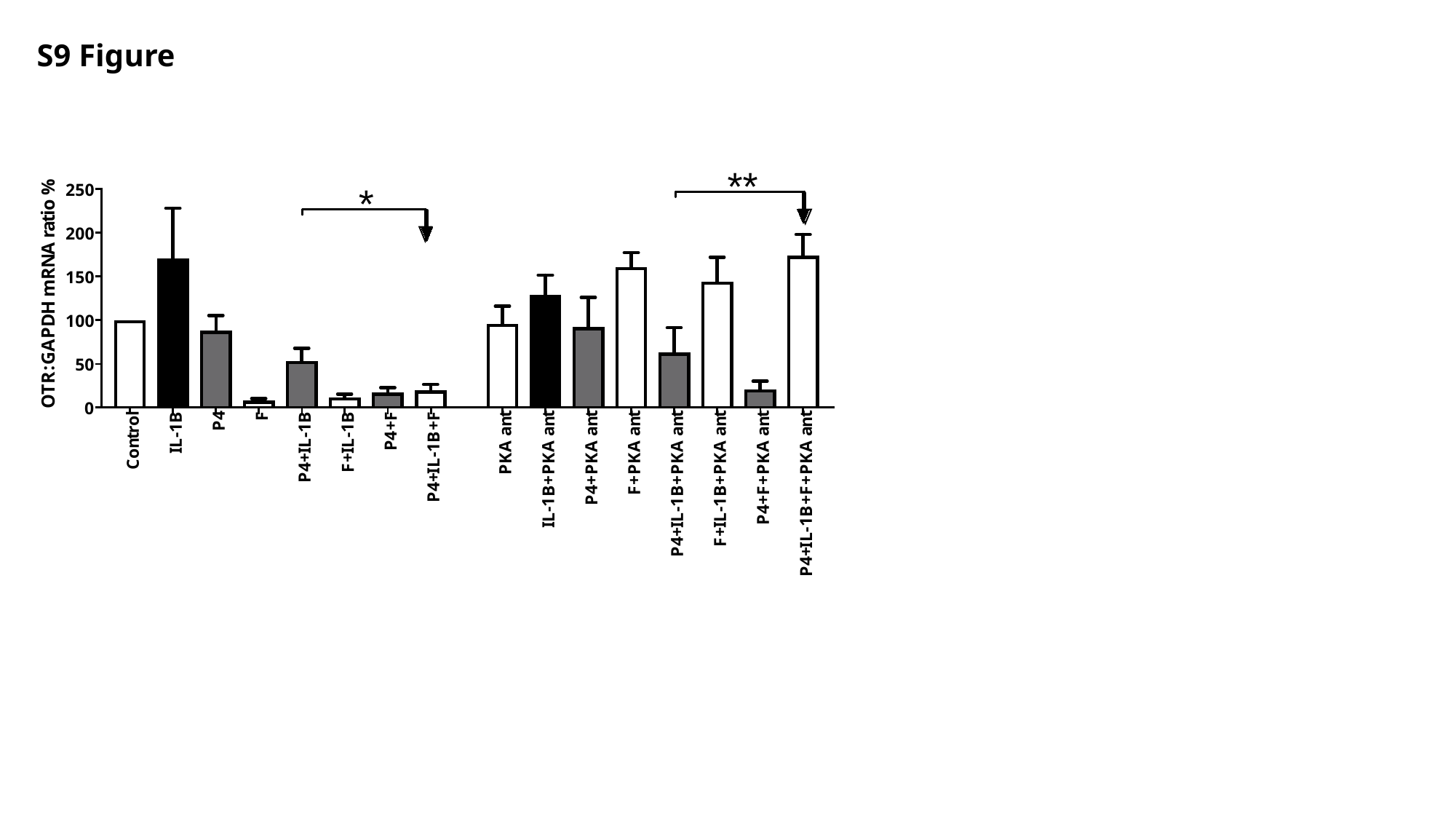

S9 Figure
**
%
*
250
o
i
t
l
t
t
t
t
t
t
t
4
F
F
F
B
B
B
o
n
n
n
n
n
n
n
P
+
+
1
1
1
r
a
a
a
a
a
a
a
t
-
-
-
4
B
L
n
L
L
P
A
A
A
A
A
A
A
1
I
I
I
o
-
+
+
K
K
K
K
K
K
K
L
C
4
F
P
P
P
P
P
P
P
I
P
+
+
+
+
+
+
+
4
4
F
F
B
B
B
P
P
+
+
1
1
1
-
-
-
4
B
L
L
L
P
1
I
I
I
-
+
+
L
4
F
I
P
+
4
P
a
200
r
A
N
R
150
m
H
D
100
P
A
G
50
:
R
T
O
0
t
n
a
A
K
P
+
F
