# Supplementary material for: The interaction between protein kinase A and progesterone on basal and inflammation-induced myometrial oxytocin receptor expression
Source: PLoS One. 2020 Dec 1;15(12):e0239937. doi: 10.1371/journal.pone.0239937 (PMC7707466; doi:10.1371/journal.pone.0239937)
Supplement: S1 File — (DOCX) [file pone.0239937.s010.docx]

S1 Table. Demographic data for term myometrial tissue samples collected.

|  | Term non labour |
| --- | --- |
| No of samples | 20 |
| Mean gestational age (GA±SD) | 39.2±1.3 |
| Maternal characteristics |  |
| Age | 34.1±5.6 |
| BMI | 26.0±6.4 |

**S2 Table. Primer pair sequences with gene accession numbers.**

| **Name** | **Primer sequence (5**’**–3**’**)** | **GenBank/**  **EMBL accession no.** |
| --- | --- | --- |
| GAPDH | F: tgatgacatcagaaggtggtgaag  R: tccttggaggccatgtaggccat | BC014085 |
| OTR | F: agaagcactcgcgcctctt  R: aggtgatgtcccacagcaact | NM000916 |
| MKP-1 | F: cagctgctgcagtttgagtc  R: aggtagctcagcgcactgtt | NM­_004417 |
| IκBα | F: ccagggctattctccctacc  R: gctcgtcctctgtgaactcc | [NM_020529](http://www.ncbi.nlm.nih.gov/entrez/viewer.fcgi?val=NM_020529) |
| 11βHSD1 | F: accttcgcagagcaatttgt  R: gccagagaggagacgacaac | NM_005525 |
| PTGES | F:catgtgagtccctgtgatgg  R:ctgcagcaaagacatccaaa | NM_004878 |
| GPR125 | F:ttggcgcagatgtgatagag  R:aagttggctgcttccacagt | NM_145290 |
| CCL8 | F:tcacctgctgctttaacgtg  R:atccctgacccatctctcct | NM_005623 |
| PDE4B | F:ccctgttgtccagtccaact  R:tgcagactctcacggtgaac | NM_001037339 |
| GPR124 | F:ctggaagagcgaaactaccg  F:gcgtgtttctgggattgtct | NM_032777 |
| CREB3L1 | F:cagatggctgggaaatcaac  R:tccttggagtgggagaagtc | NM_052854.3 |
| GUCY1A3 | F:caagttgtgcaagccaagaa  R: atccagctctccacactgct | NM_000856 |
| PRKG1 | F:caggcccagatcctatgaaa  R:ccctcaaaccatttgtgctt | NM_001098512 |

**S3 Table. ON-TARGET plus SMART pool siRNA sequences.**

| **Name** | **Source** |
| --- | --- |
| Non-Targeting | ON-TARGET plus Non-Targeting siRNA: Dharmacon, D-001810-01-05 |
| PKAC-α | ON-TARGET plus Human PRKACA (5566) siRNA: Dharmacon, L-004649-00-0005 |
| EPAC1 | ON-TARGET plus Human RAPGEF3 (10411) siRNA: Dharmacon, L-007676-00-0005 |
| AMPK | OriGene Technologies, Rockville, MD, USA |

**S4 Table. Primary Antibodies**

**Primary antibodies**

AMPK_α_ (62 kDa): New England BioLabs Ltd., 2532, Herts, UK

α-tubulin (55 kDa): Santa Cruz Biochemicals, SC-8035, Texas, USA

EPAC1 (100 kDa): New England BioLabs Ltd., 4155, Herts, UK

IκBα (C-21) (39 KDa): Santa Cruz Biochemicals, SC-371, Texas, USA

GAPDH (38kDa): Millipore MAB374, Watford, UK

OTR-R (C-20) (66 kDa): Santa Cruz Biochemicals, SC-8013, Texas, USA

phospho-p38 (43 kDa): New England BioLabs Ltd., 9211, Herts, UK

phospho-JNK (46, 54 KDa): New England BioLabs Ltd., 9251, Herts, UK

PKA C-α (42 KDa): New England BioLabs Ltd., 4782, Herts, UK

phospho-NF-κB p65 (65 kDa): New England BioLabs Ltd., 3031, Herts, UK

TATA binding protein(38 kDa): Abcam Ltd., ab818, Cambridge, UK

c-jun (43-48 kDa): New England BioLabs Ltd., 9165, Herts, UK

c-fos (62 kDa): New England BioLabs Ltd., 4384, Herts, UK

phospho-c-jun: New England BioLabs Ltd.,54B3, Herts, UK

MKP1 (c-19) (40kDa): Santa Cruz Biochemicals, SC-370, Texas, USA
