# Supplementary material for: The interaction between protein kinase A and progesterone on basal and inflammation-induced myometrial oxytocin receptor expression
Source: PLoS One. 2020 Dec 1;15(12):e0239937. doi: 10.1371/journal.pone.0239937 (PMC7707466; doi:10.1371/journal.pone.0239937)

Figure 1B OTR (66 kDa)

LANE A

1: Positive control
2: Sample 1 control
3: IL1B
4: Forskolin
5: P4
6: IL1B + forskolin
7: Sample 2 control
8: IL1B
9: Forskolin
10: IL1B + forskolin
11-15: empty wells
16: Positive control
17: Sample 3 control
18: IL1B
19: Forskolin
20: P4
21: IL1B + forskolin
22: Sample 4 control
23: IL1B
24: Forskolin
25: IL1B + forskolin

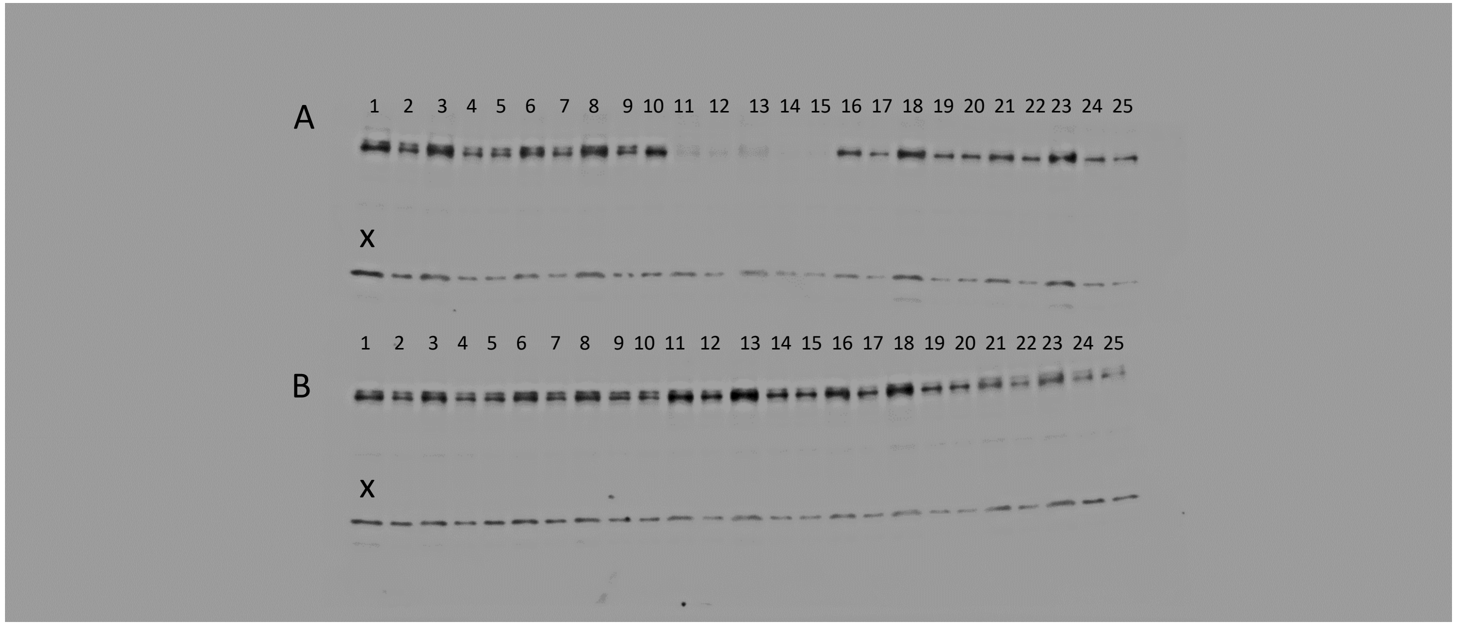

LANE B

1: Positive control
2: Sample 5 control
3: IL1B
4: Forskolin
5: P4
6: IL1B + forskolin
7: Sample 6 control
8: IL1B
9: Forskolin
10: IL1B + forskolin
11: Positive control
12: Sample 7 control
13: IL1B
14: Forskolin
15: P4
16: IL1B + forskolin
17: Sample 8 control
18: IL1B
19: Forskolin
20: IL1B + forskolin
21: Positive control
22: Sample 9 control
23: IL1B
24: Forskolin
25: IL1B + forskolin

Figure 1B OTR (66 kDa) – additional samples

LANE A

1: Positive control
2: Sample 1 control
3: IL1B
4: Forskolin
5: P4
6: IL1B + forskolin
7: Sample 2 control
8: IL1B
9: Forskolin
10: IL1B + forskolin
11-15: empty wells
16: Positive control
17: Sample 3 control
18: IL1B
19: Forskolin
20: P4
21: IL1B + forskolin
22: Sample 4 control
23: IL1B
24: Forskolin
25: IL1B + forskolin

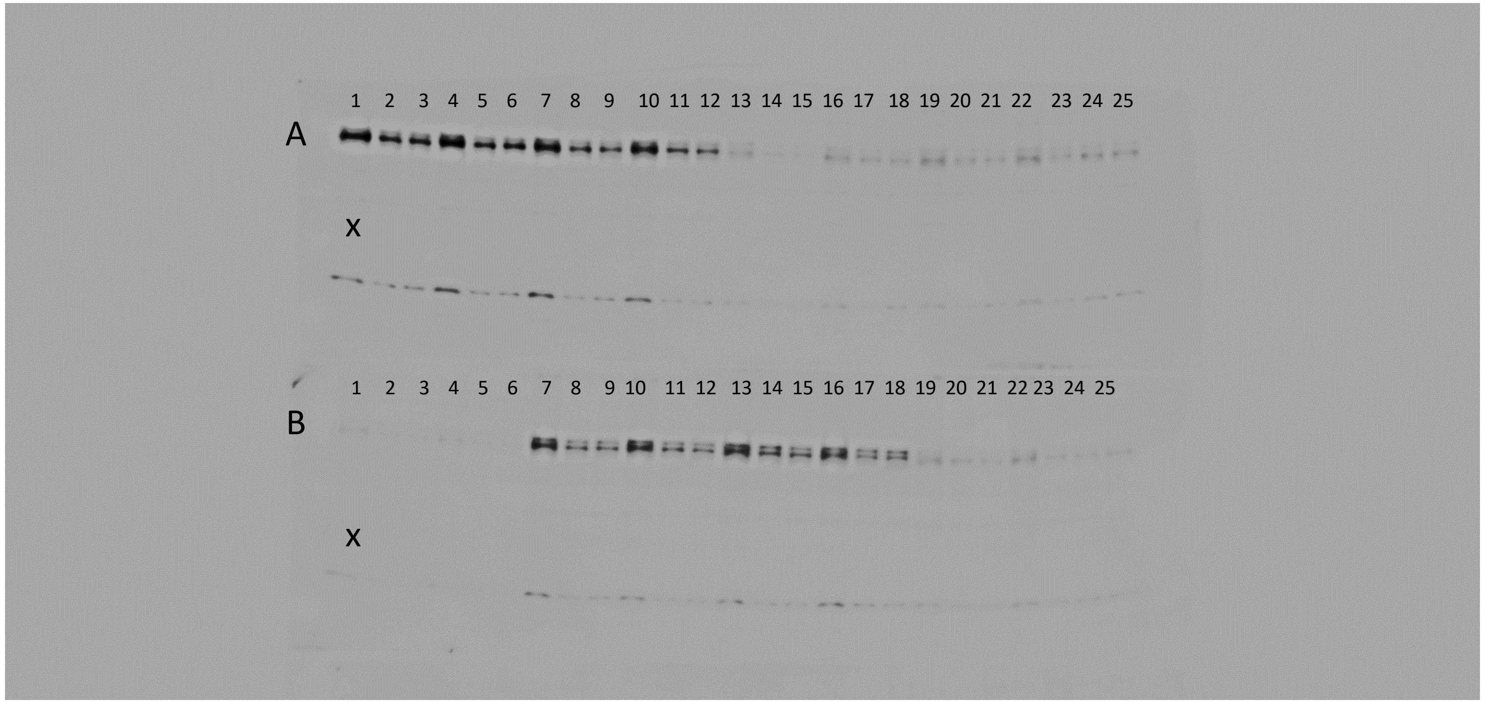

LANE B

1: Positive control
2: Sample 5 control
3: IL1B
4: Forskolin
5: P4
6: IL1B + forskolin
7: Sample 6 control
8: IL1B
9: Forskolin
10: IL1B + forskolin
11: Positive control
12: Sample 7 control
13: IL1B
14: Forskolin
15: P4
16: IL1B + forskolin
17: Sample 8 control
18: IL1B
19: Forskolin
20: IL1B + forskolin
21: Positive control
22: Sample 9 control
23: IL1B
24: Forskolin
25: IL1B + forskolin

Figure 1B GAPDH (38 kDa) (for OTR - equivalent labelling as per slide 1 & 2)

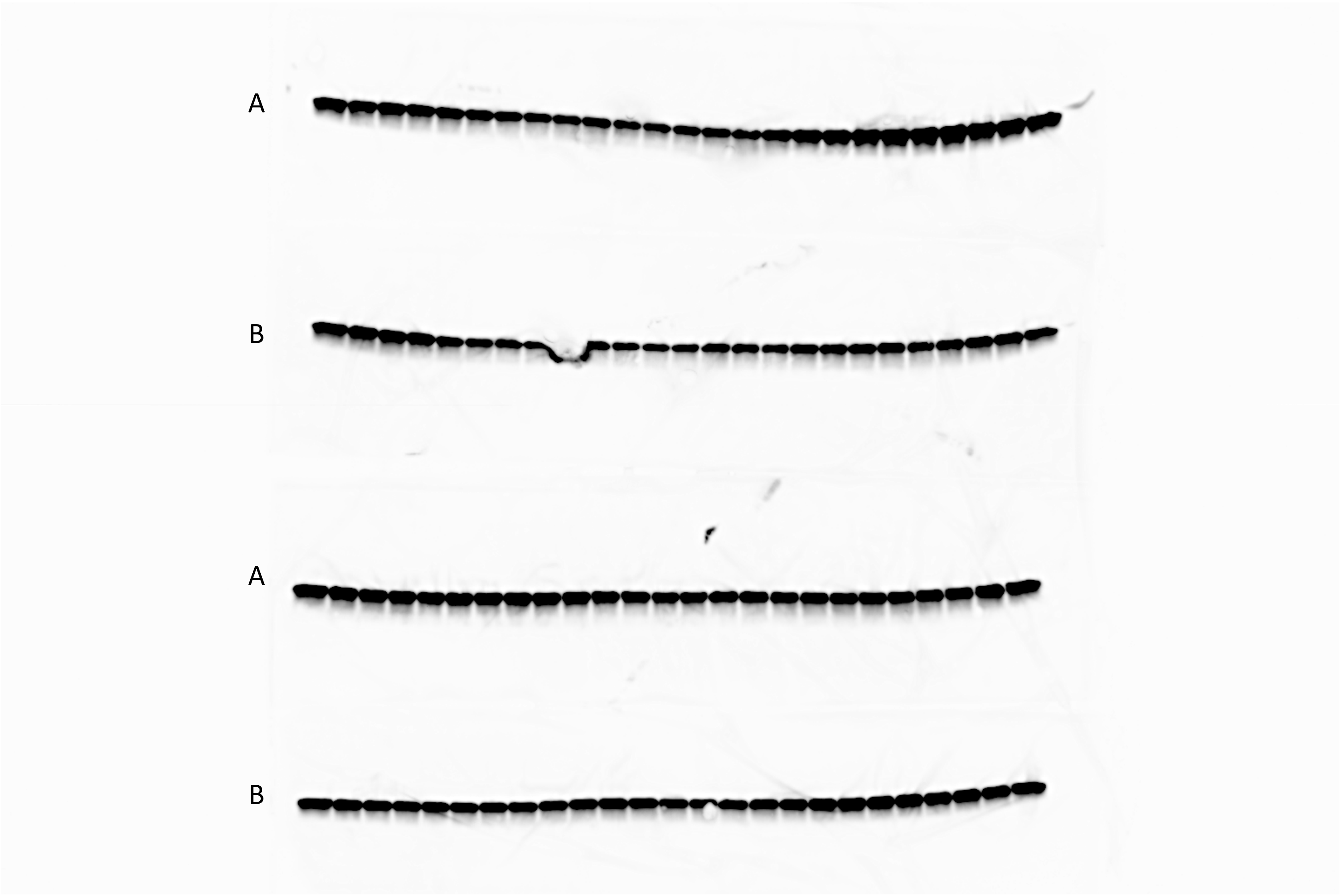

Figure 1B - Digital photo

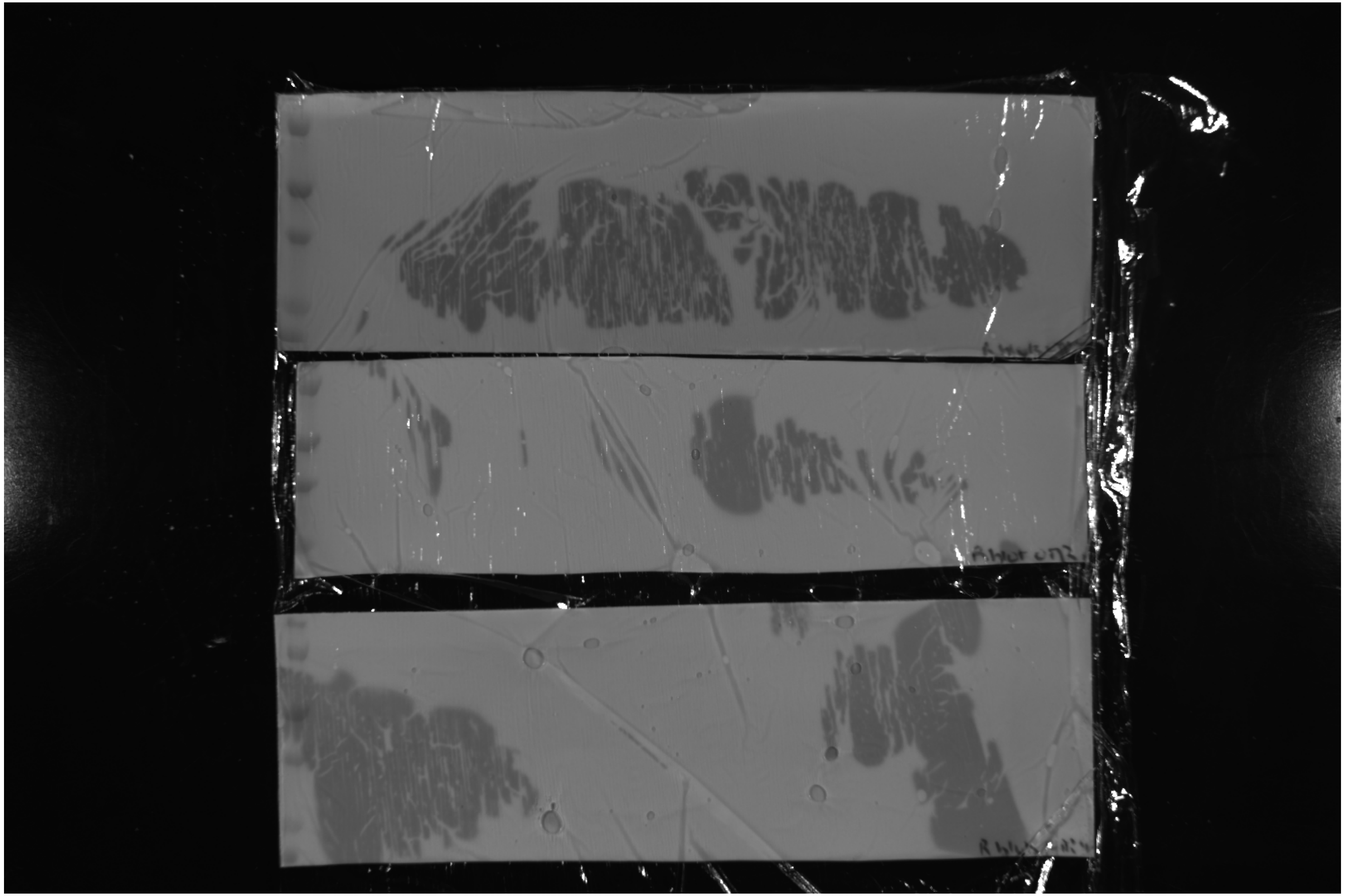

Figure 2A Total P65 (65 kDa) – Sample A

LANE A – top lane

1: Control
2: IL1B
3: 0.1 µM P4
4: 1 µM P4
5: 10 µM P4
6: Forskolin
7: IL1B + forskolin
8: 0.1 µM P4 + IL1B
9: 1 µM P4 + IL1B
10: 10 µM P4 + IL1B
11: 0.1 µM P4 + IL1B + forskolin
12: 1 µM P4 + IL1B + forskolin
13: 10 µM P4 + IL1B + forksolin
14: Positive control

6 hours treatment

Multiple experimental conditions not included in the manuscript.

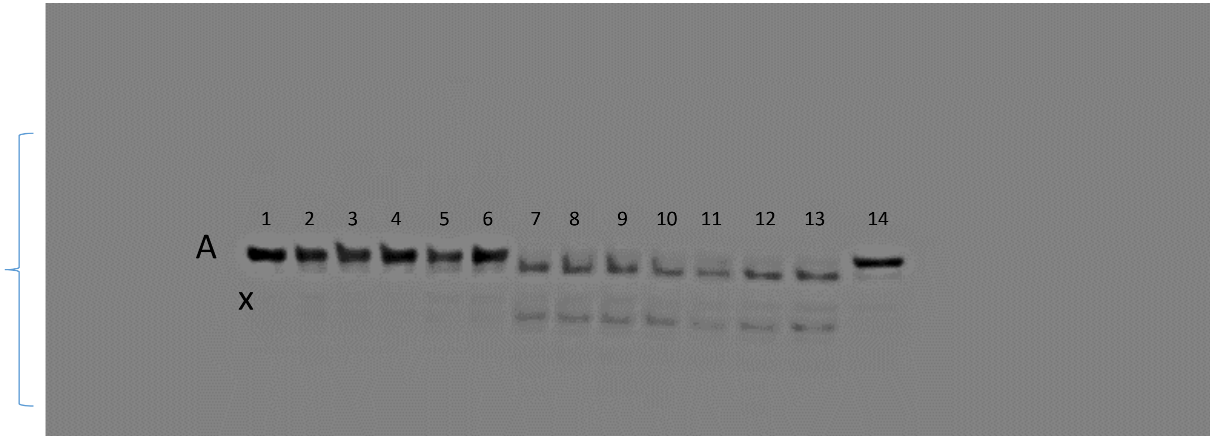

Figure 2A – Sample A digital photo

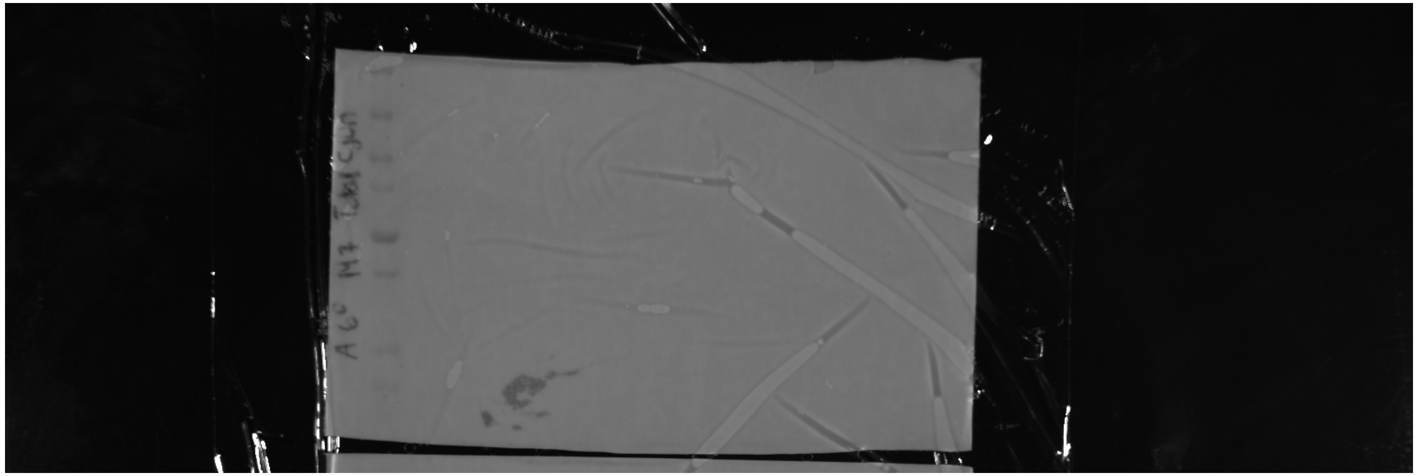

LANE A – second lane

1: Control
2: IL1B
3: 0.1 µM P4
4: 1 µM P4
5: 10 µM P4
6: Forskolin
7: IL1B + forskolin
8: 0.1 µM P4 + IL1B
9: 1 µM P4 + IL1B
10: 10 µM P4 + IL1B
11: 0.1 µM P4 + IL1B + forskolin
12: 1 µM P4 + IL1B + forskolin
13: 10 µM P4 + IL1B + forksolin
14: Positive control

6 hours treatment

Multiple experimental conditions not included in the manuscript.

Figure 2A Total P65 (65 kDa) – Sample B

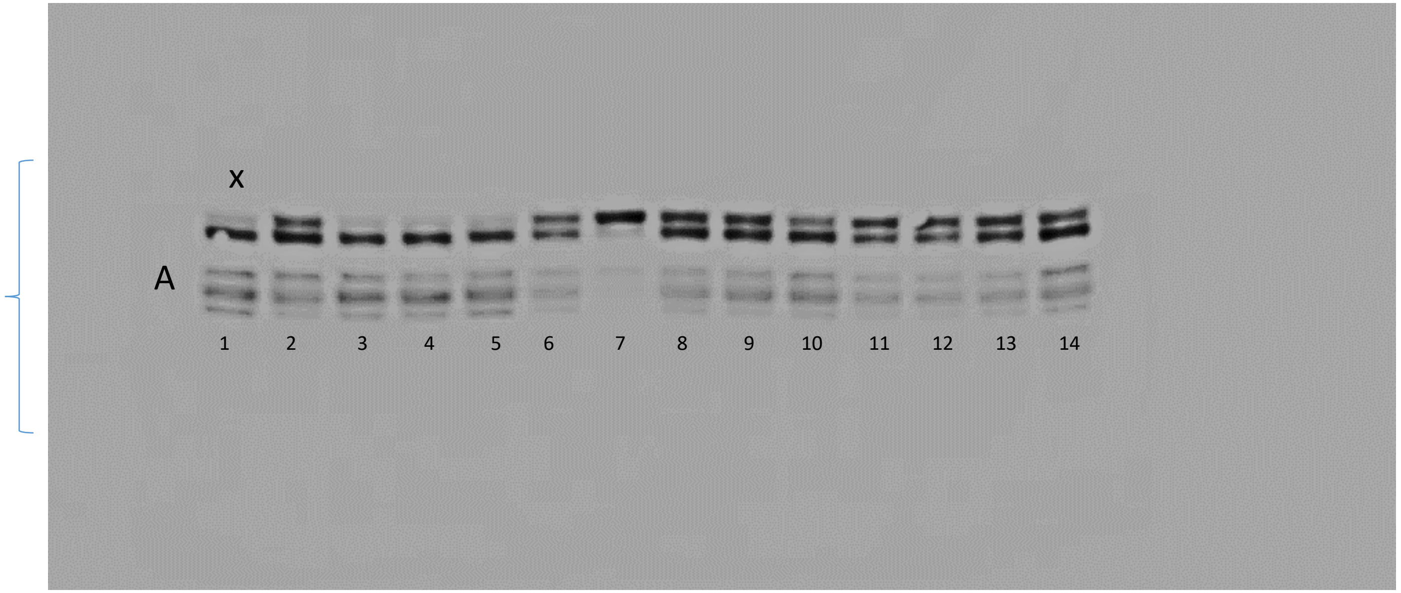

Digital photo

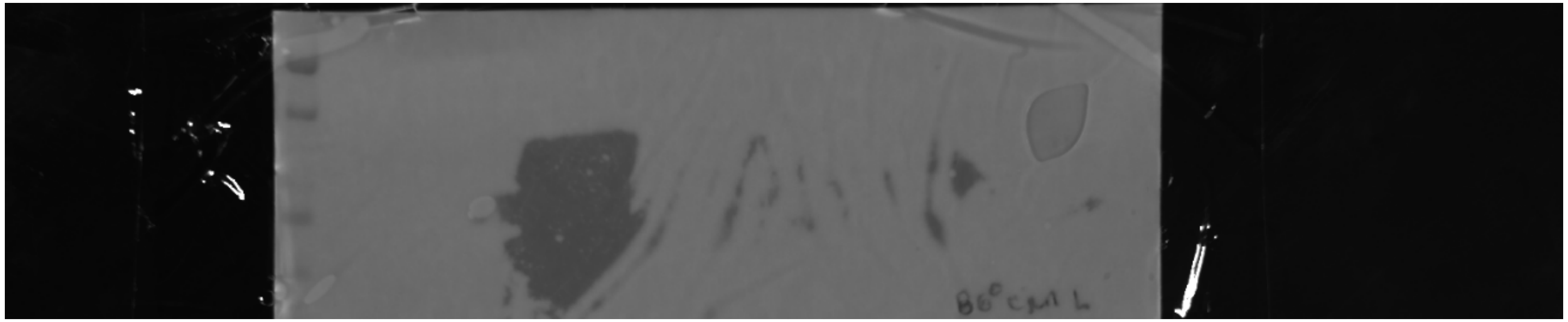

Figure 2A Total P65 (65 kDa) – Sample C

LANE A – top lane

1: Control
2: IL1B
3: 0.1 µM P4
4: 1 µM P4
5: 10 µM P4
6: Forskolin
7: IL1B + forskolin
8: 0.1 µM P4 + IL1B
9: 1 µM P4 + IL1B
10: 10 µM P4 + IL1B
11: 0.1 µM P4 + IL1B + forskolin
12: 1 µM P4 + IL1B + forskolin
13: 10 µM P4 + IL1B + forksolin
14: Positive control

6 hours treatment

Multiple experimental conditions not included in the manuscript.

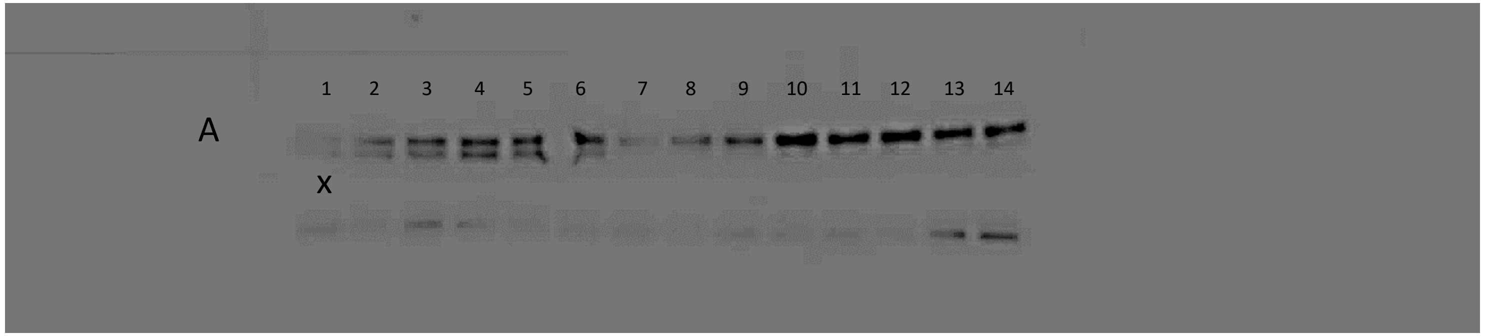

Digital photo

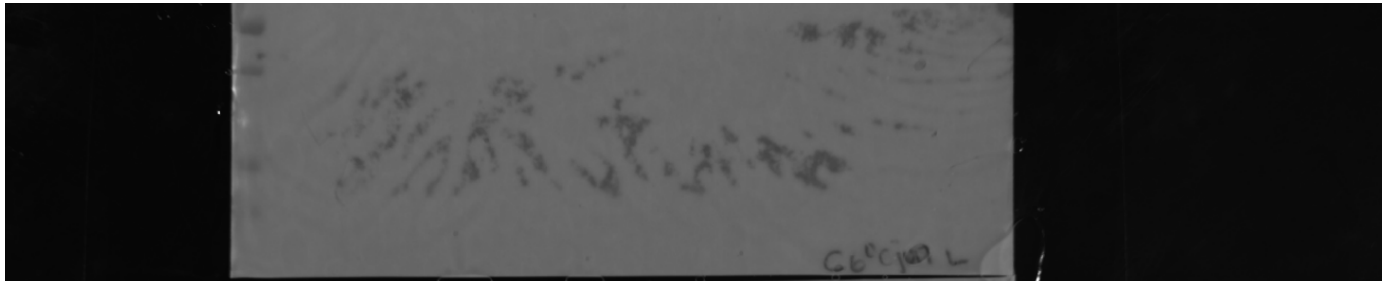

Figure 2A Total P65 (65 kDa) – Sample E

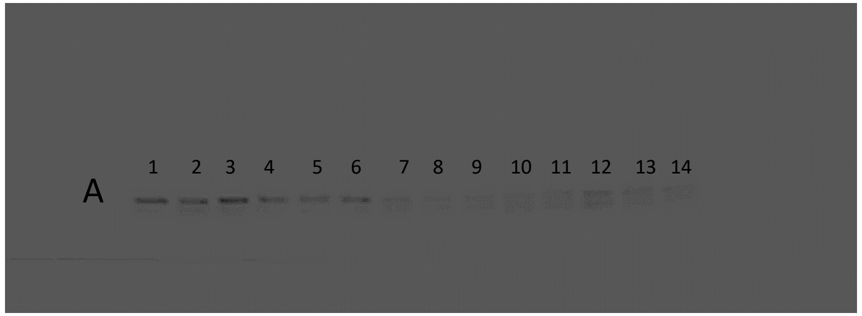

Digital photo

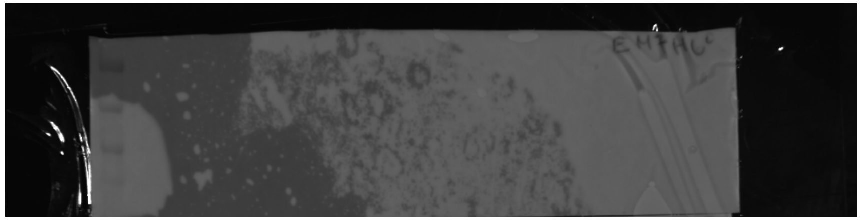

LANE A

1: Control
2: IL1B
3: 0.1 µM P4
4: 1 µM P4
5: 10 µM P4
6: Forskolin
7: IL1B + forskolin
8: 0.1 µM P4 + IL1B
9: 1 µM P4 + IL1B
10: 10 µM P4 + IL1B
11: 0.1 µM P4 + IL1B + forskolin
12: 1 µM P4 + IL1B + forskolin
13: 10 µM P4 + IL1B + forksolin
14: Positive control

6 hours treatment

Multiple experimental conditions not included in the manuscript.

Figure 2A Total P65 (65 kDa) – Sample F & G

LANE A

1: Control
2: IL1B
3: 0.1 µM P4
4: 1 µM P4
5: 10 µM P4
6: Forskolin
7: IL1B + forskolin
8: 0.1 µM P4 + IL1B
9: 1 µM P4 + IL1B
10: 10 µM P4 + IL1B
11: 0.1 µM P4 + IL1B + forskolin
12: 1 µM P4 + IL1B + forskolin
13: 10 µM P4 + IL1B + forksolin
14: Positive control

6 hours treatment

Multiple experimental conditions not included in the manuscript.

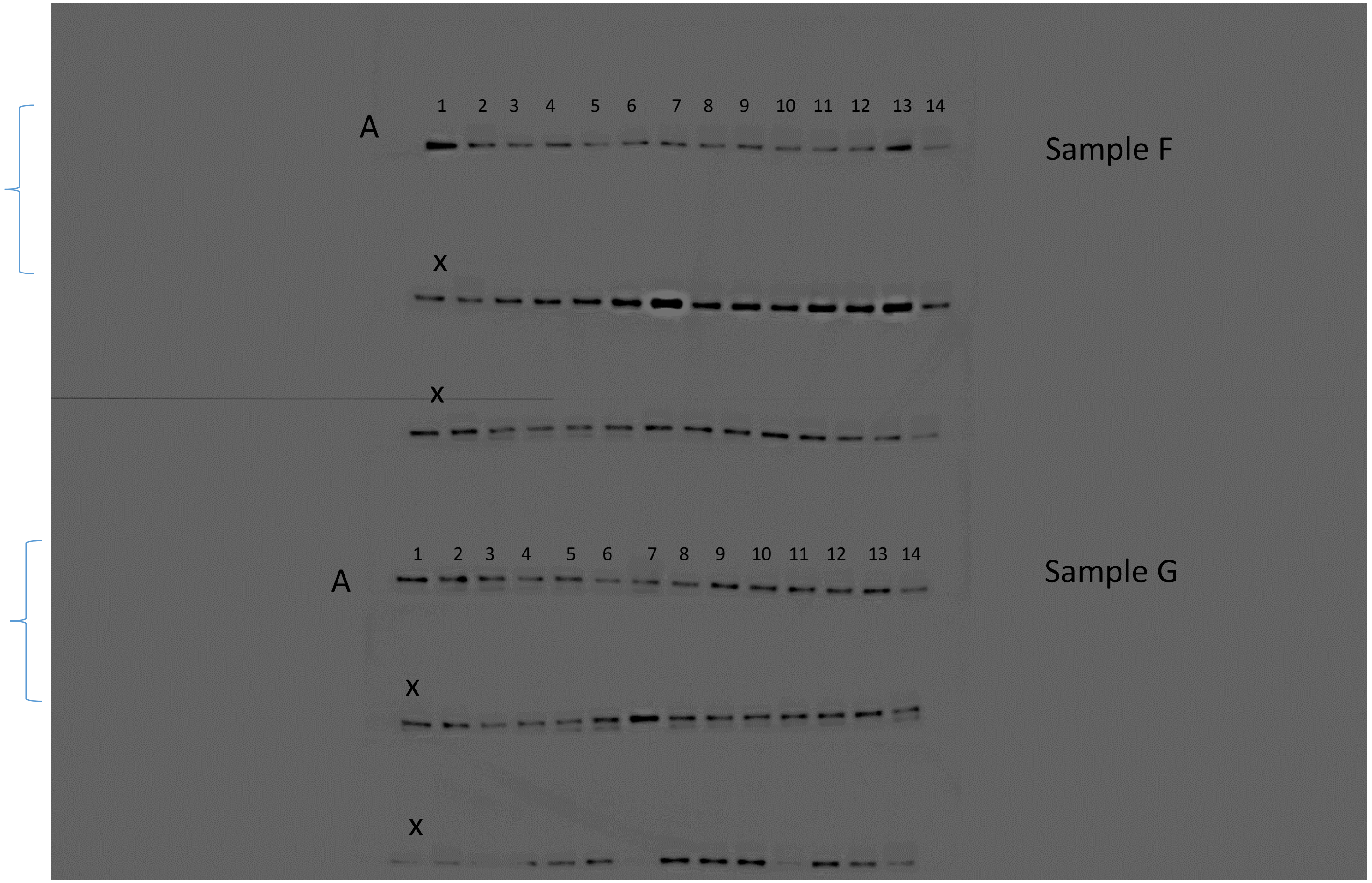

Figure 2A Sample F & G – digital photo

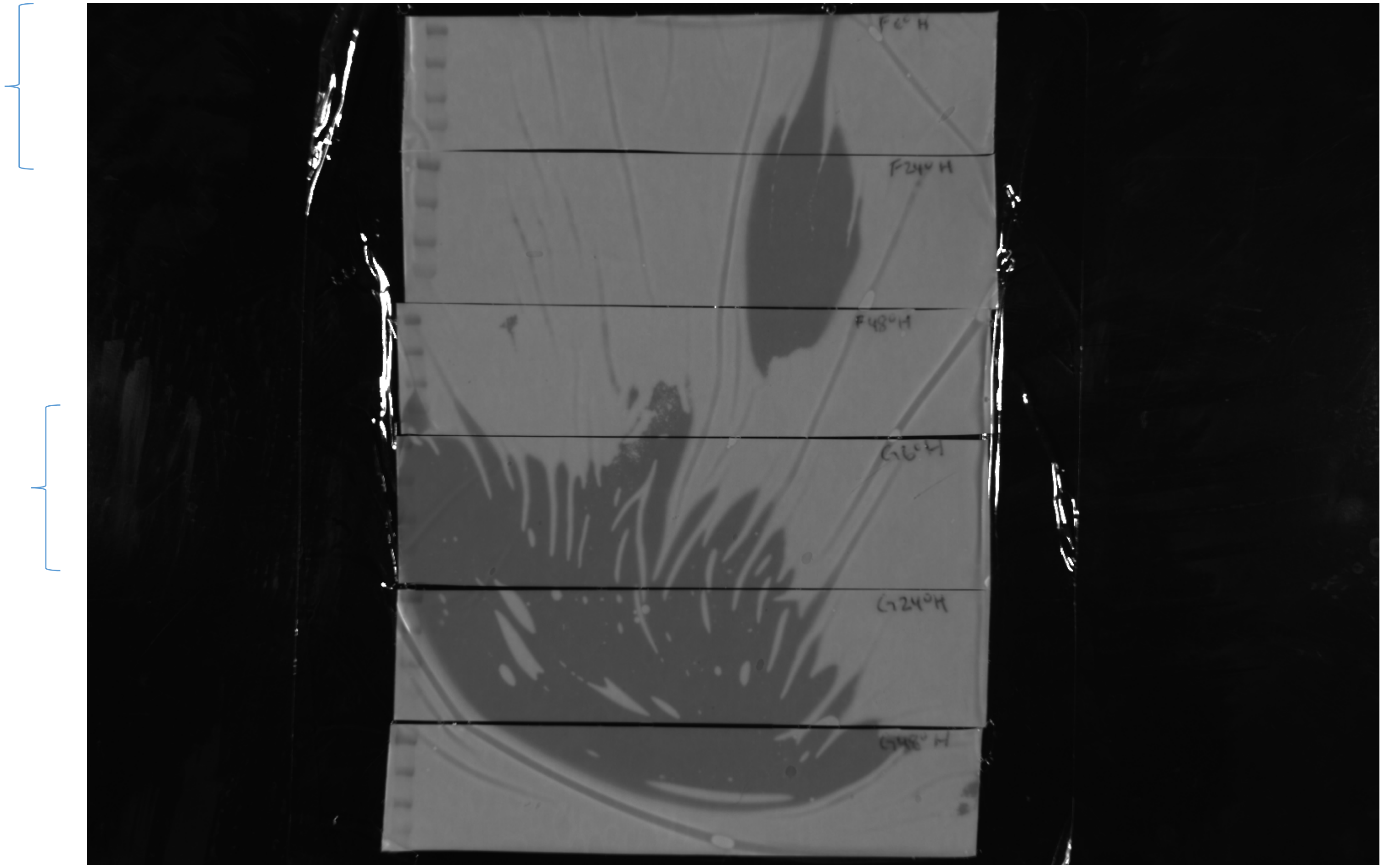

Figure 2B Total c-jun (43-48 kDa) – Sample A

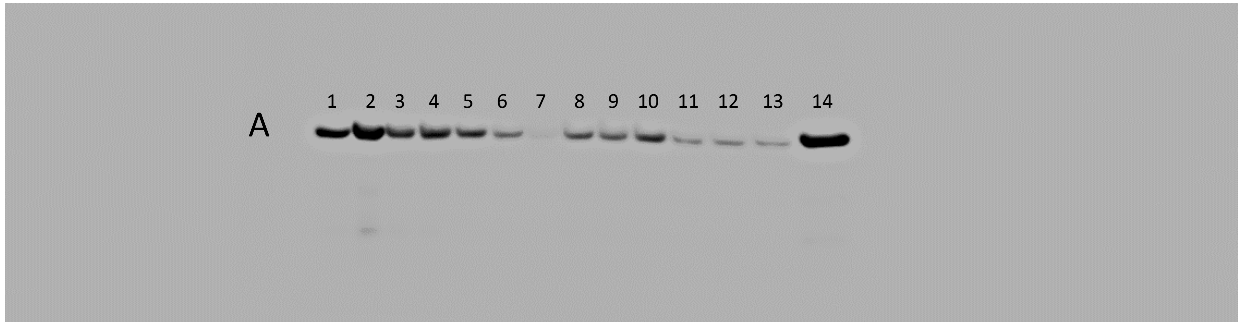

Digital photo

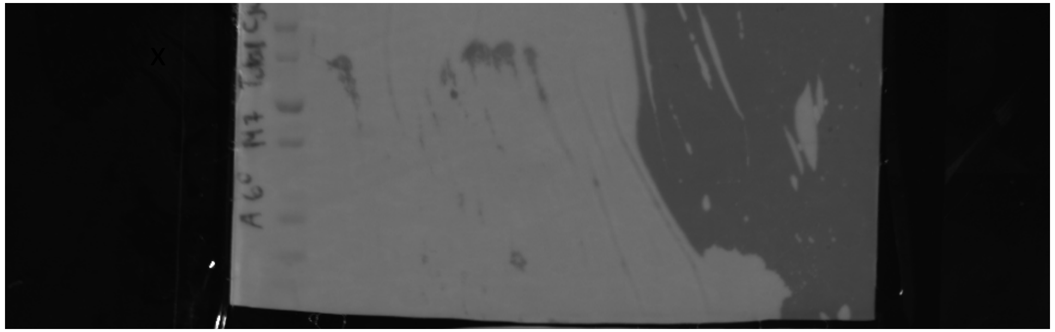

LANE A

1: Control
2: IL1B
3: 0.1 µM P4
4: 1 µM P4
5: 10 µM P4
6: Forskolin
7: IL1B + forskolin
8: 0.1 µM P4 + IL1B
9: 1 µM P4 + IL1B
10: 10 µM P4 + IL1B
11: 0.1 µM P4 + IL1B + forskolin
12: 1 µM P4 + IL1B + forskolin
13: 10 µM P4 + IL1B + forksolin
14: Positive control

6 hours treatment

Multiple experimental conditions not included in the manuscript.

Figure 2B Total c-jun (43-48 kDa) – Sample B

LANE A

1: Control
2: IL1B
3: 0.1 µM P4
4: 1 µM P4
5: 10 µM P4
6: Forskolin
7: IL1B + forskolin
8: 0.1 µM P4 + IL1B
9: 1 µM P4 + IL1B
10: 10 µM P4 + IL1B
11: 0.1 µM P4 + IL1B + forskolin
12: 1 µM P4 + IL1B + forskolin
13: 10 µM P4 + IL1B + forksolin
14: Positive control

6 hours treatment

Multiple experimental conditions not included in the manuscript.

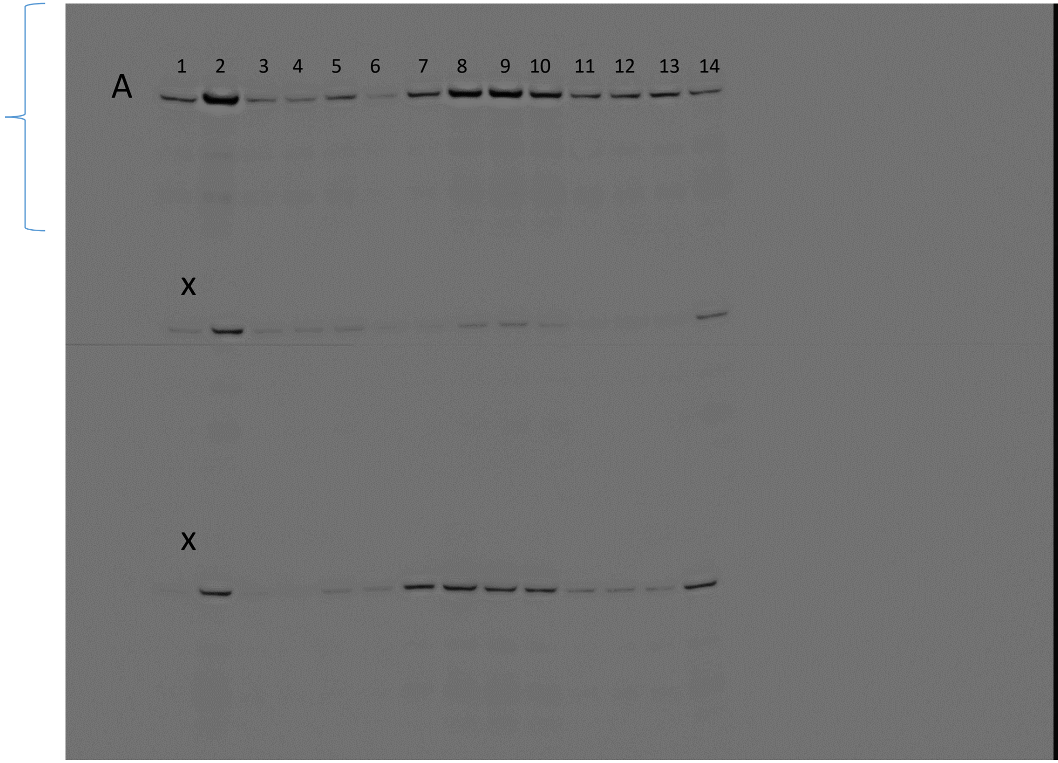

Digital photo

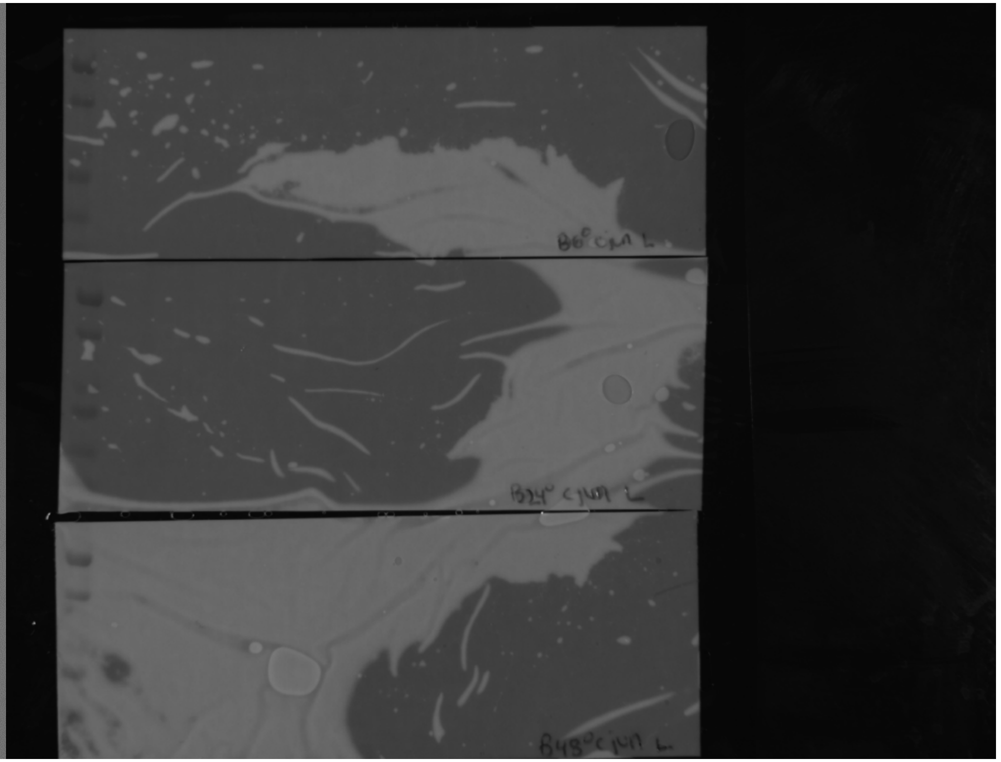

Figure 2B Total c-jun (43-48 kDa) – Sample D

LANE A

1: Control
2: IL1B
3: 0.1 µM P4
4: 1 µM P4
5: 10 µM P4
6: Forskolin
7: IL1B + forskolin
8: 0.1 µM P4 + IL1B
9: 1 µM P4 + IL1B
10: 10 µM P4 + IL1B
11: 0.1 µM P4 + IL1B + forskolin
12: 1 µM P4 + IL1B + forskolin
13: 10 µM P4 + IL1B + forksolin
14: Positive control

6 hours treatment

Multiple experimental conditions not included in the manuscript.

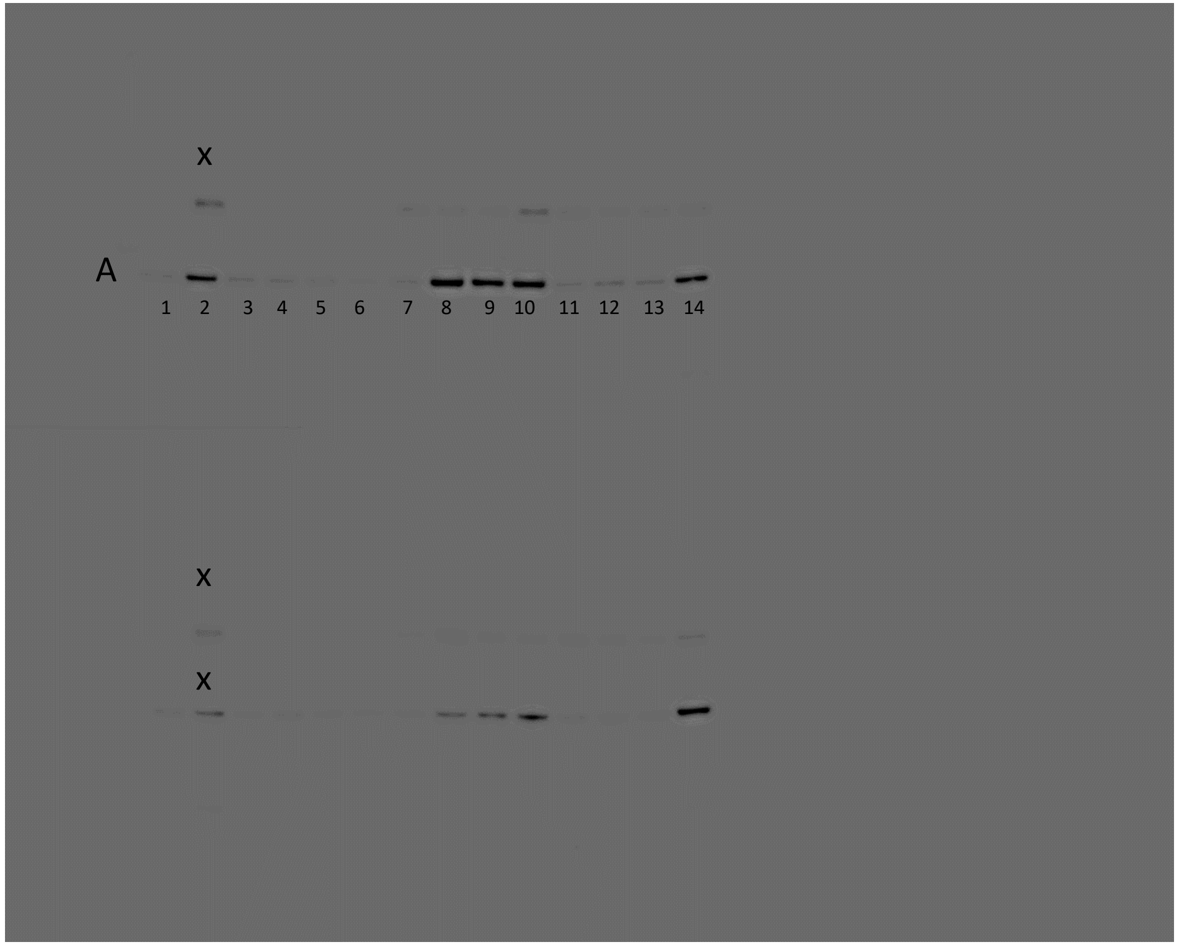

Digital photo

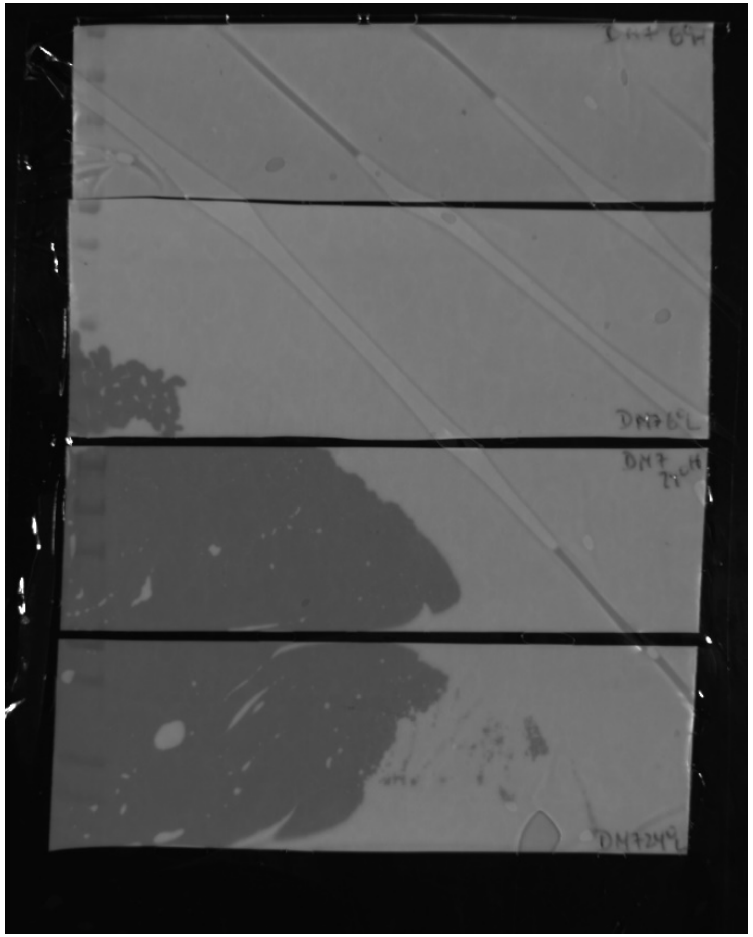

Figure 2B Total c-jun (43-48 kDa) – Sample E

LANE A

1: Control
2: IL1B
3: 0.1 µM P4
4: 1 µM P4
5: 10 µM P4
6: Forskolin
7: IL1B + forskolin
8: 0.1 µM P4 + IL1B
9: 1 µM P4 + IL1B
10: 10 µM P4 + IL1B
11: 0.1 µM P4 + IL1B + forskolin
12: 1 µM P4 + IL1B + forskolin
13: 10 µM P4 + IL1B + forksolin
14: Positive control

6 hours treatment

Multiple experimental conditions not included in the manuscript.

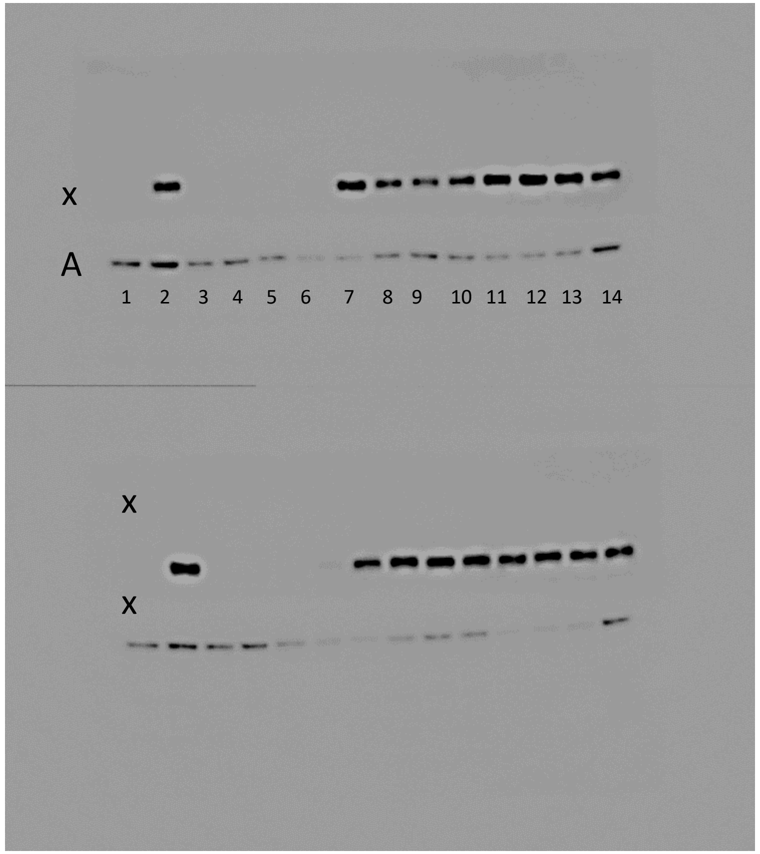

Digital photo

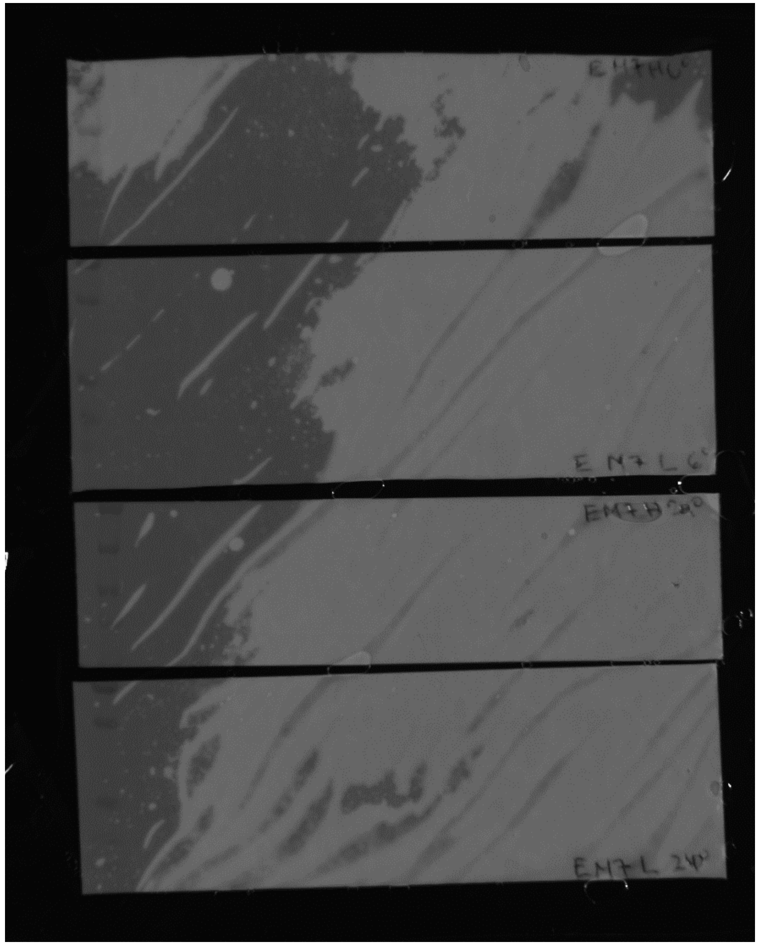

Figure 2B Total c-jun (43-48 kDa) – Sample F

LANE A

1: Control
2: IL1B
3: 0.1 µM P4
4: 1 µM P4
5: 10 µM P4
6: Forskolin
7: IL1B + forskolin
8: 0.1 µM P4 + IL1B
9: 1 µM P4 + IL1B
10: 10 µM P4 + IL1B
11: 0.1 µM P4 + IL1B + forskolin
12: 1 µM P4 + IL1B + forskolin
13: 10 µM P4 + IL1B + forksolin
14: Positive control

6 hours treatment

Multiple experimental conditions not included in the manuscript.

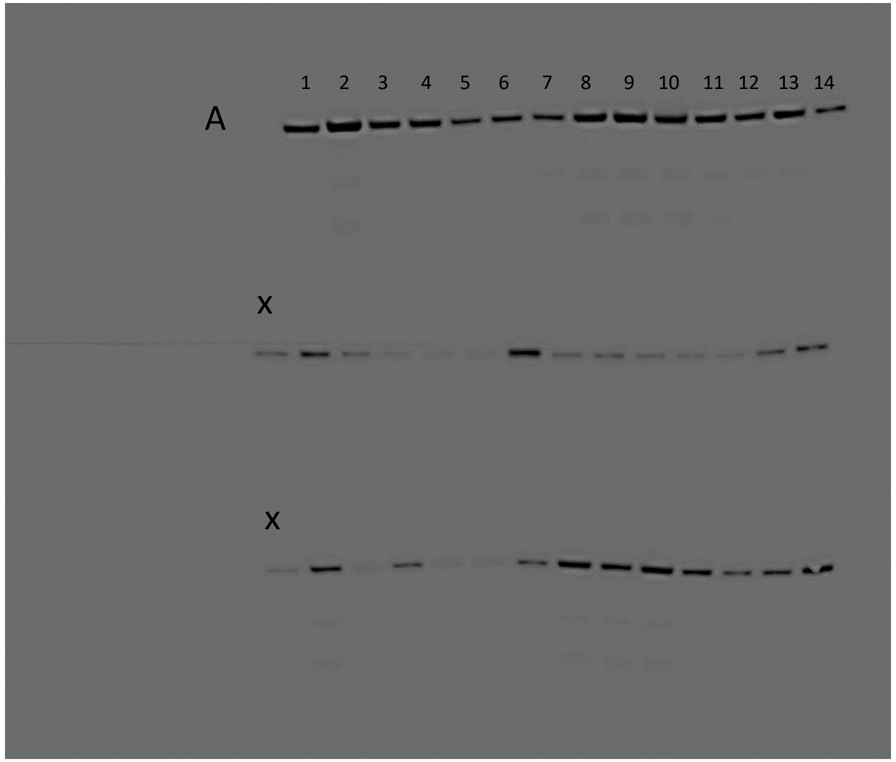

Digital photo

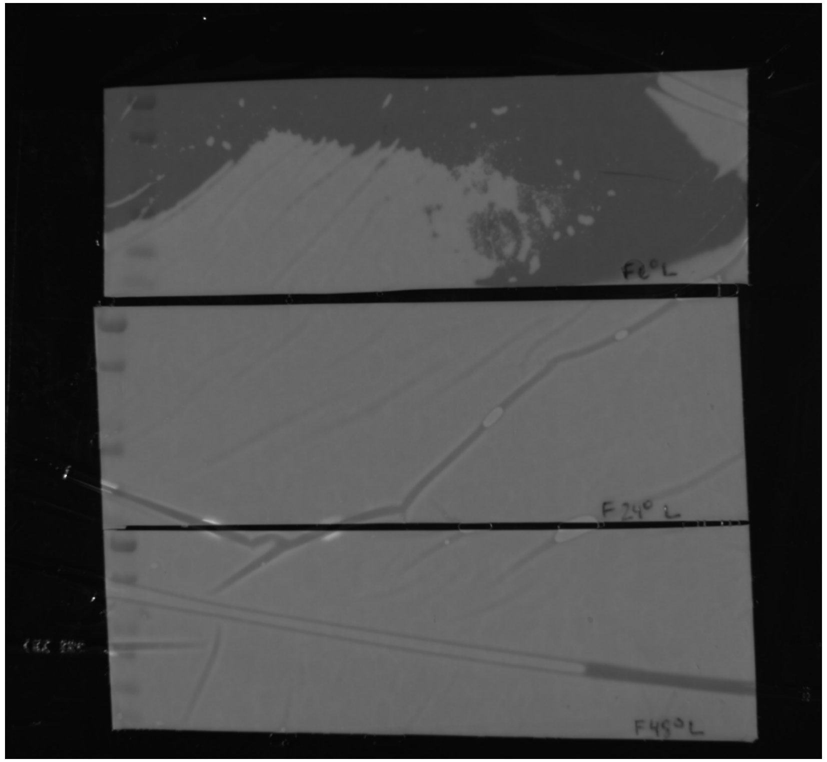

Figure 2B Total c-jun (43-48 kDa) – Sample G

LANE A

1: Control
2: IL1B
3: 0.1 µM P4
4: 1 µM P4
5: 10 µM P4
6: Forskolin
7: IL1B + forskolin
8: 0.1 µM P4 + IL1B
9: 1 µM P4 + IL1B
10: 10 µM P4 + IL1B
11: 0.1 µM P4 + IL1B + forskolin
12: 1 µM P4 + IL1B + forskolin
13: 10 µM P4 + IL1B + forksolin
14: Positive control

6 hours treatment

Multiple experimental conditions not included in the manuscript.

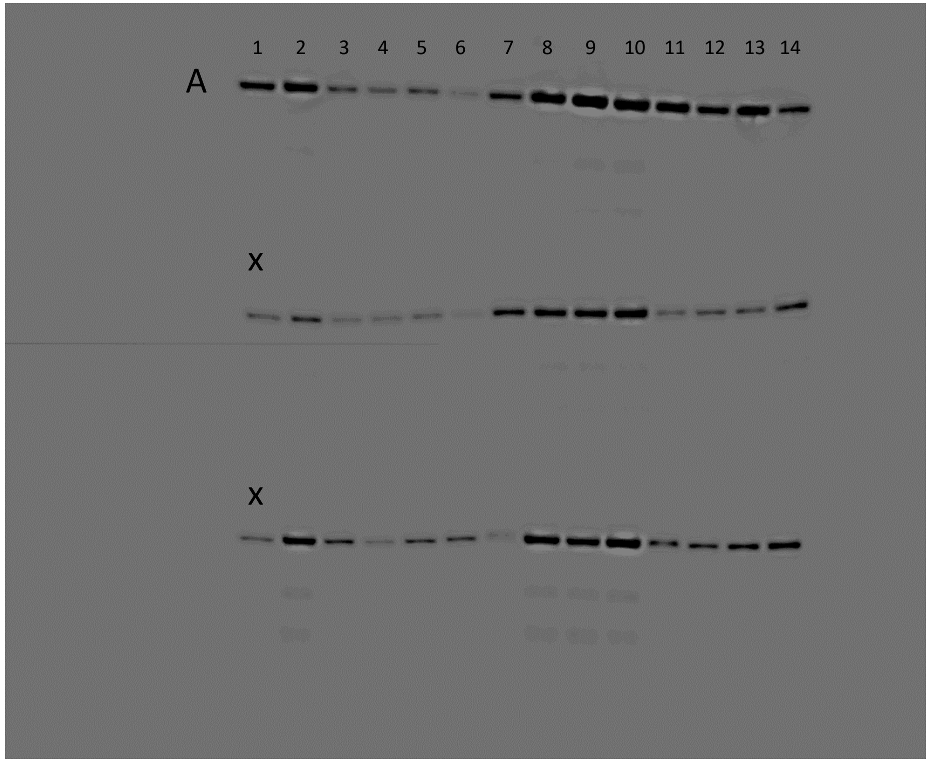

Digital photo

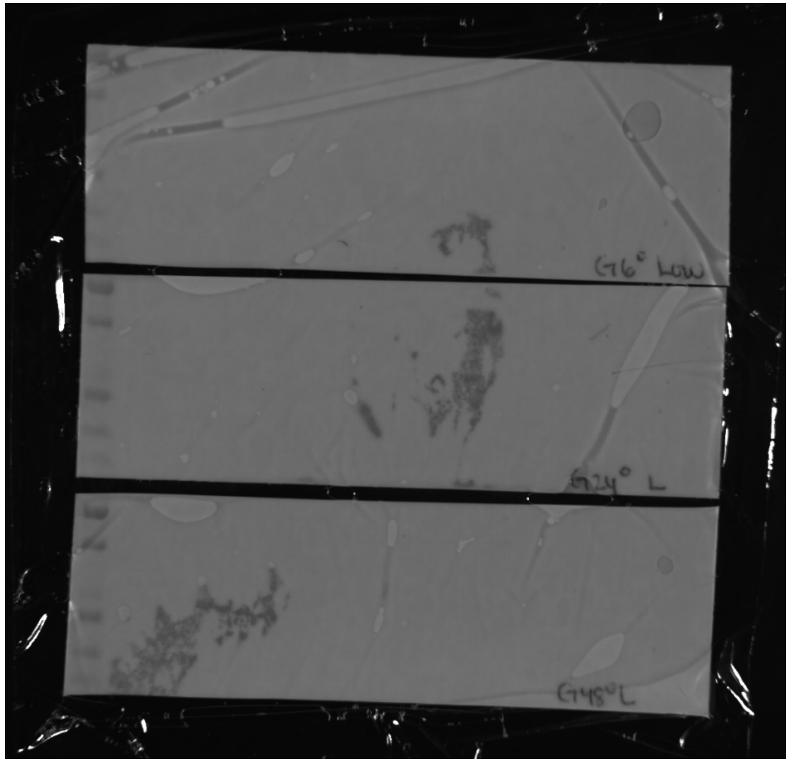

Figure 2A&B GAPDH (38 kDa) (for total p65 & total c-jun - equivalent labelling as per slide 5 to 11 & 12 to 17)

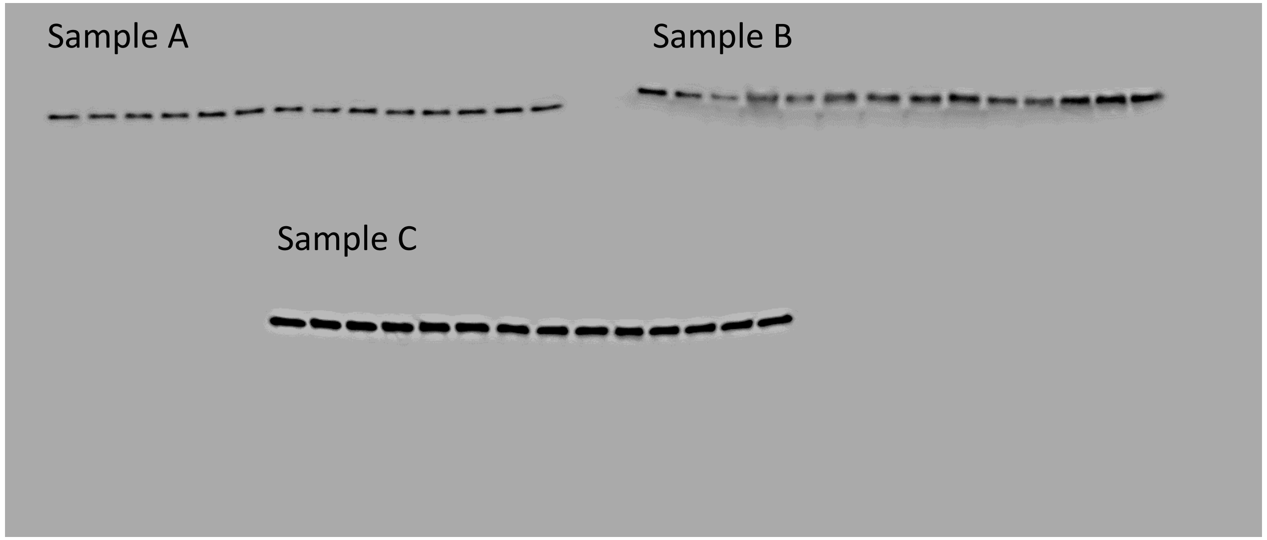

Figure 2A&B GAPDH (38 kDa) (for total p65 & total c-jun - equivalent labelling as per slide 5 to 11 & 12 to 17)

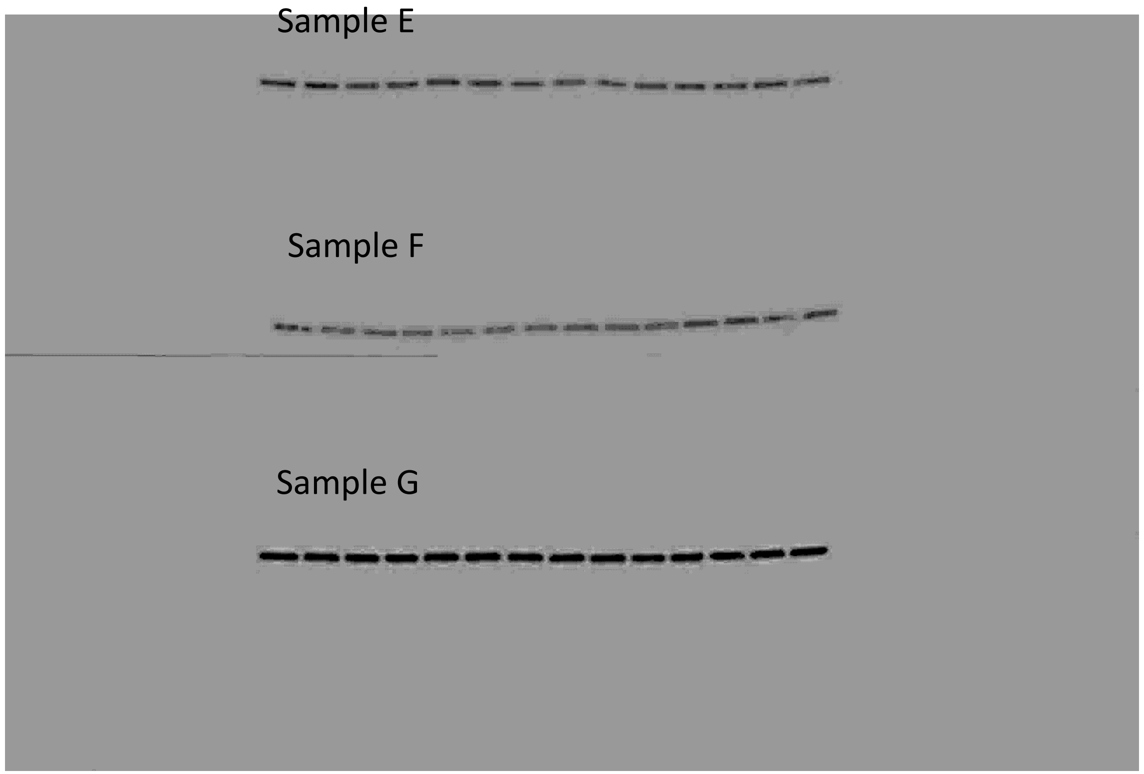

Figure 2C Phospho-P65 Nuclear (65 kDa)

1: Control
2: IL1B
3: P4
4: Forskolin
5: Forskolin + IL1B
6: P4 + IL1B
7: P4 + forskolin
8: P4 + forskolin + IL1B

60-minute time point used

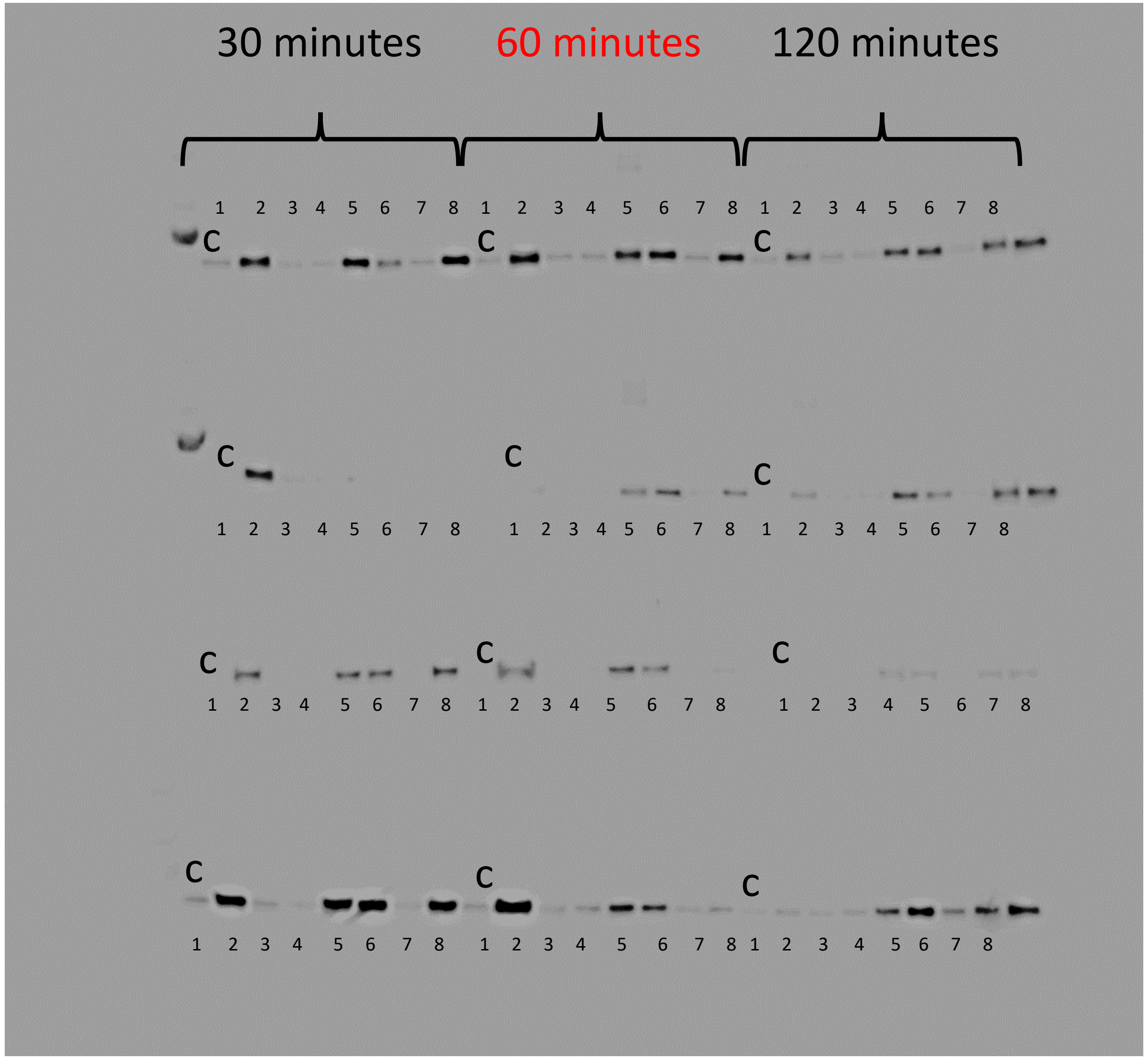

Figure 2C Phospho-P65 Nuclear (65 kDa) – additional samples

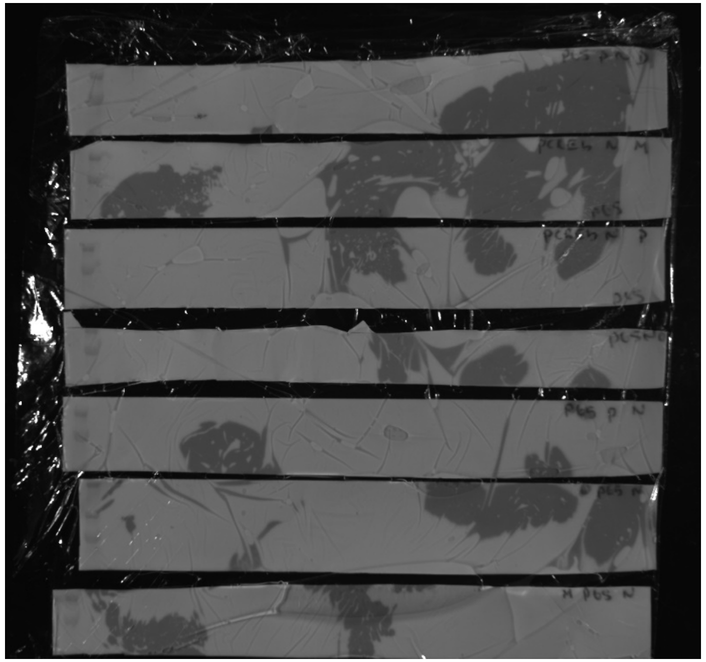

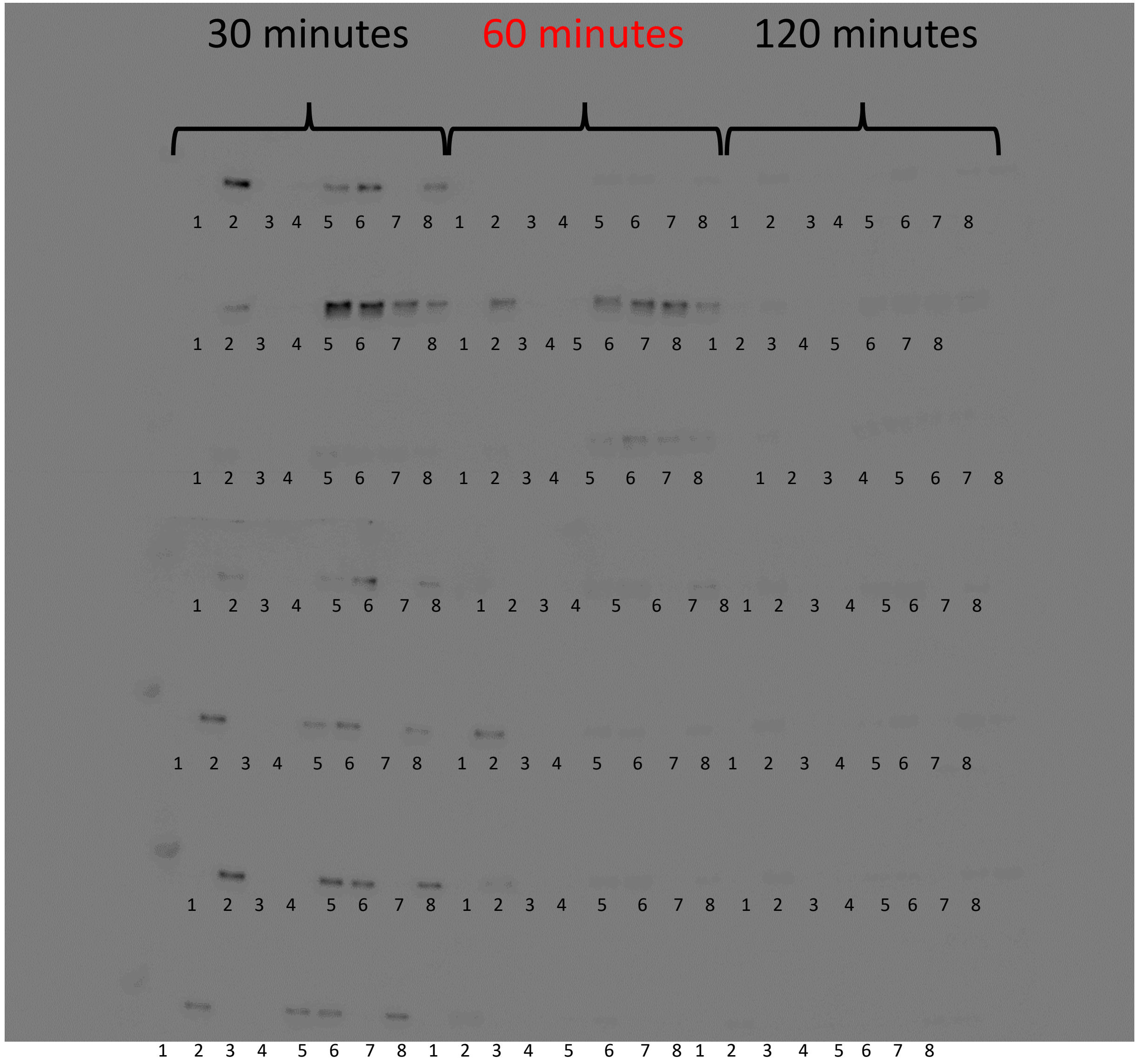

1: Control
2: IL1B
3: P4
4: Forskolin
5: Forskolin + IL1B
6: P4 + IL1B
7: P4 + forskolin
8: P4 + forskolin + IL1B

60-minute time point used

Figure 2C Phospho-P65 Nuclear (65 kDa) – additional samples

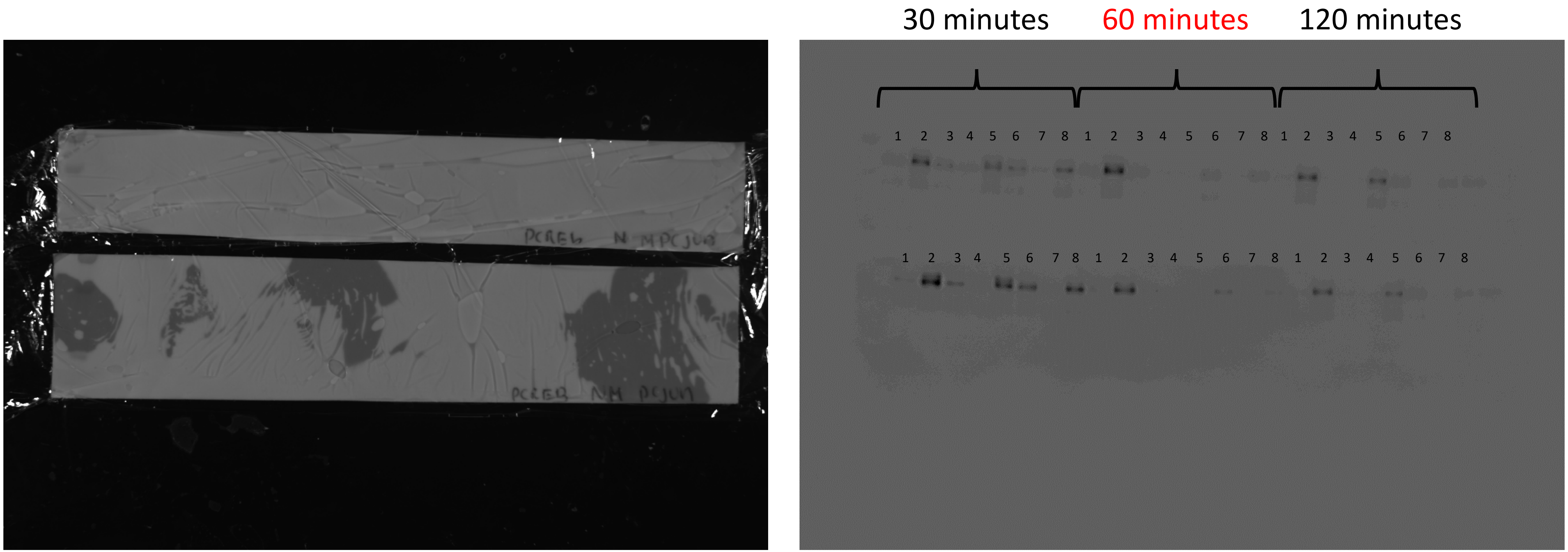

1: Control
2: IL1B
3: P4
4: Forskolin
5: Forskolin + IL1B
6: P4 + IL1B
7: P4 + forskolin
8: P4 + forskolin + IL1B

60-minute time point used

Figure 2D Phospho-P65 Cytoplasmic (65 kDa)

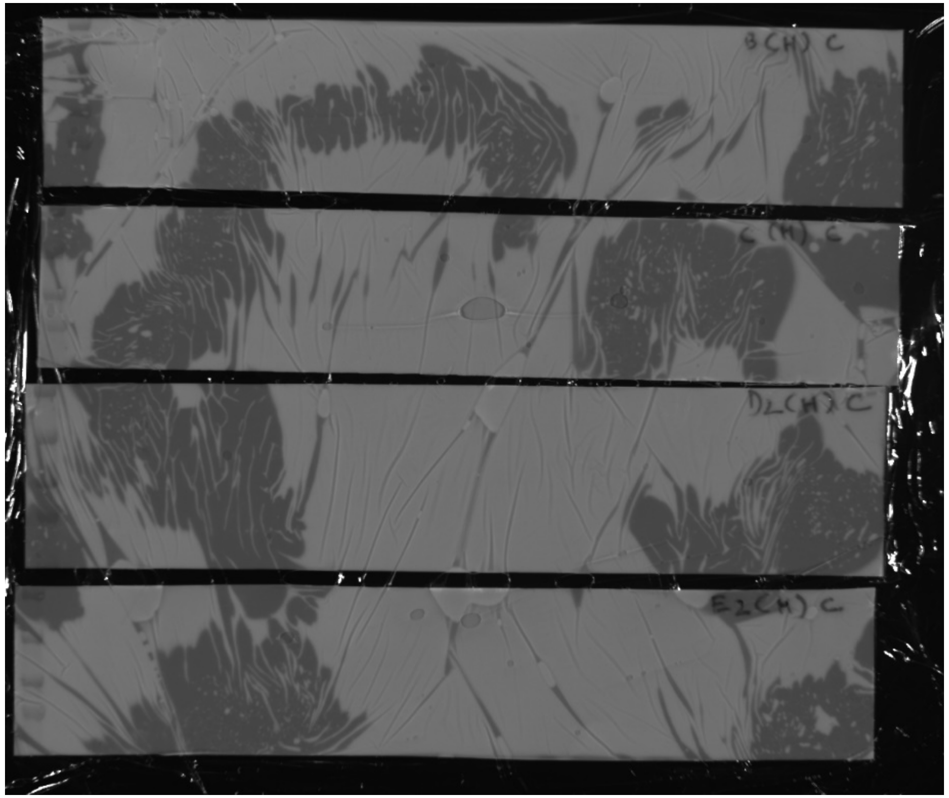

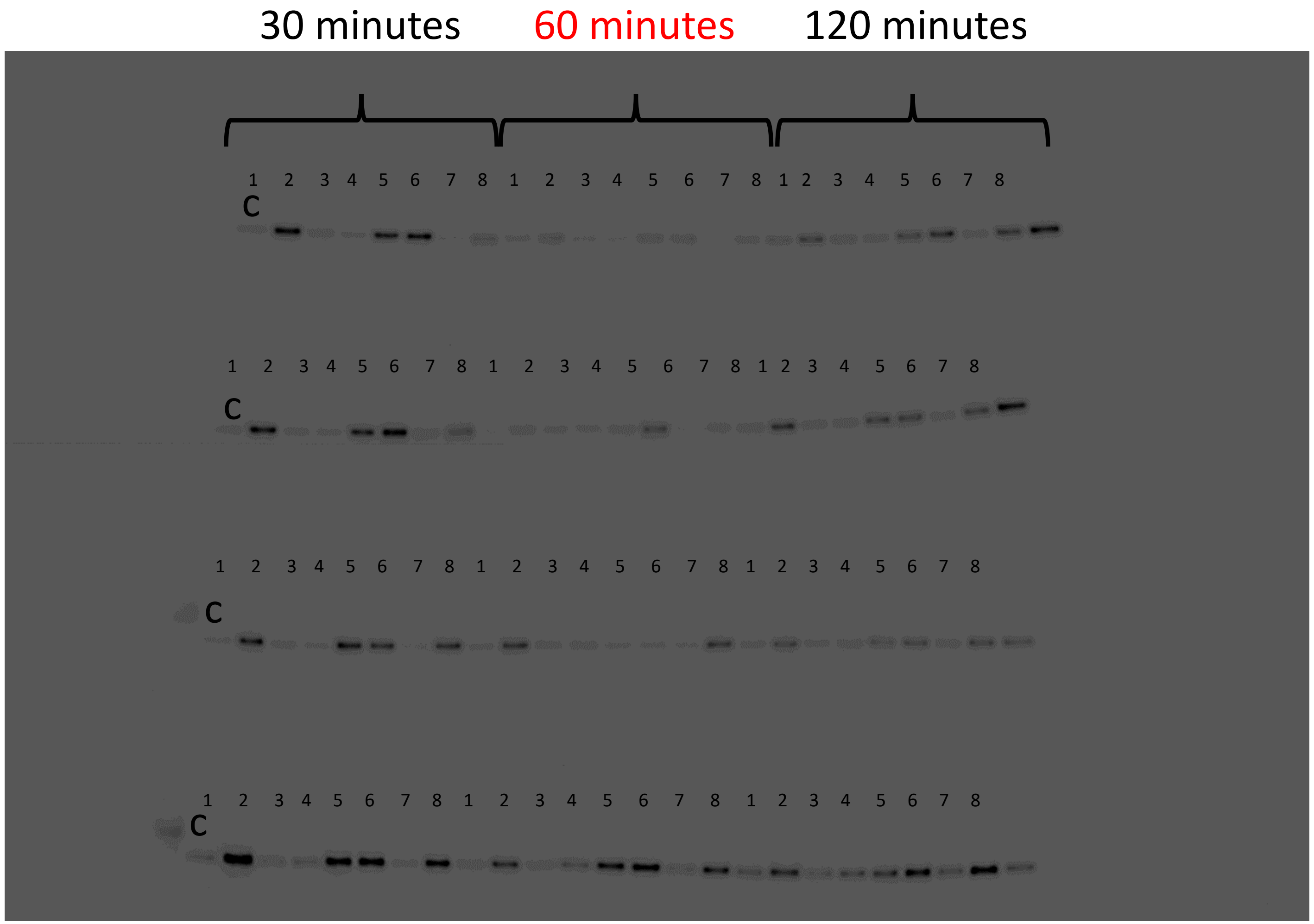

1: Control
2: IL1B
3: P4
4: Forskolin
5: Forskolin + IL1B
6: P4 + IL1B
7: P4 + forskolin
8: P4 + forskolin + IL1B

60-minute time point used

Figure 2E Phospho-c-jun Nuclear (43-48 kDa)

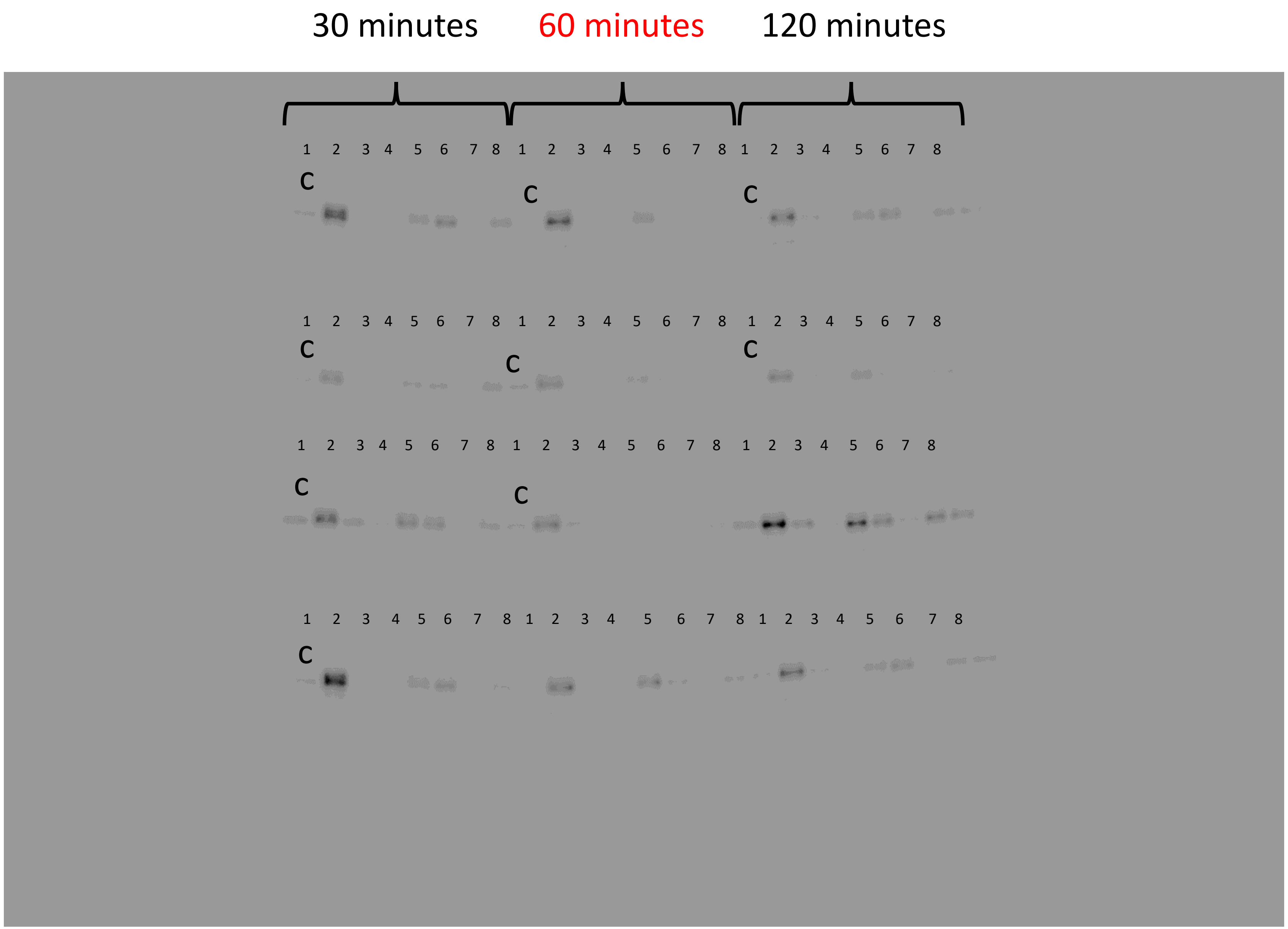

1: Control
2: IL1B
3: P4
4: Forskolin
5: Forskolin + IL1B
6: P4 + IL1B
7: P4 + forskolin
8: P4 + forskolin + IL1B

60-minute time point used

Figure 2E Phospho-c-jun Nuclear (43-48 kDa) – additional samples

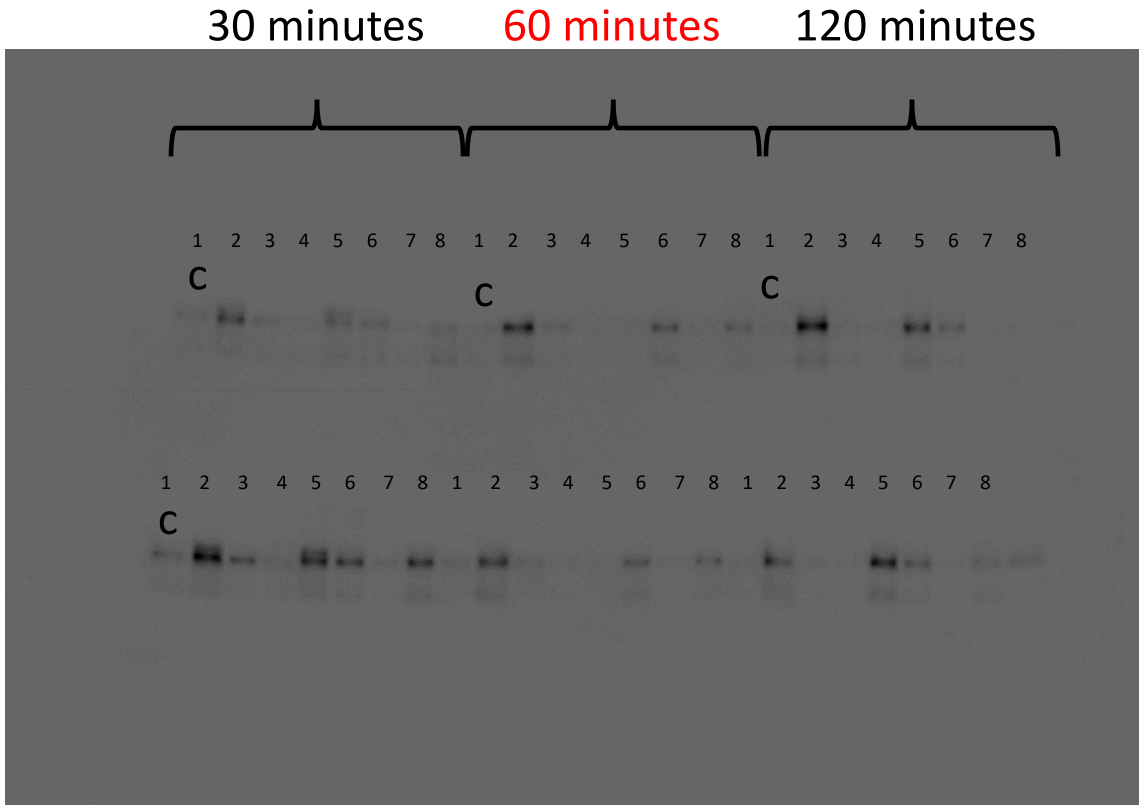

1: Control
2: IL1B
3: P4
4: Forskolin
5: Forskolin + IL1B
6: P4 + IL1B
7: P4 + forskolin
8: P4 + forskolin + IL1B

60-minute time point used

Figure 2F Phospho-c-jun Cytoplasmic (43-48 kDa)

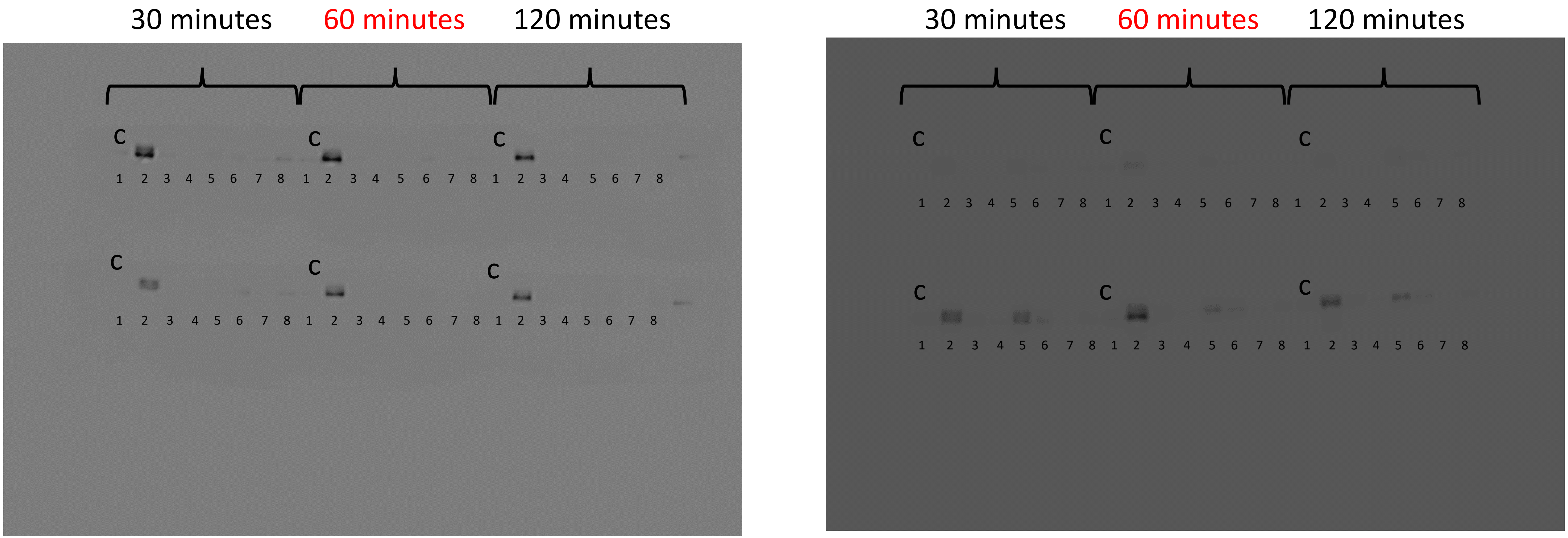

1: Control
2: IL1B
3: P4
4: Forskolin
5: Forskolin + IL1B
6: P4 + IL1B
7: P4 + forskolin
8: P4 + forskolin + IL1B

60-minute time point used

Figure 2F Phospho-c-jun Cytoplasmic (43-48 kDa) – additional samples

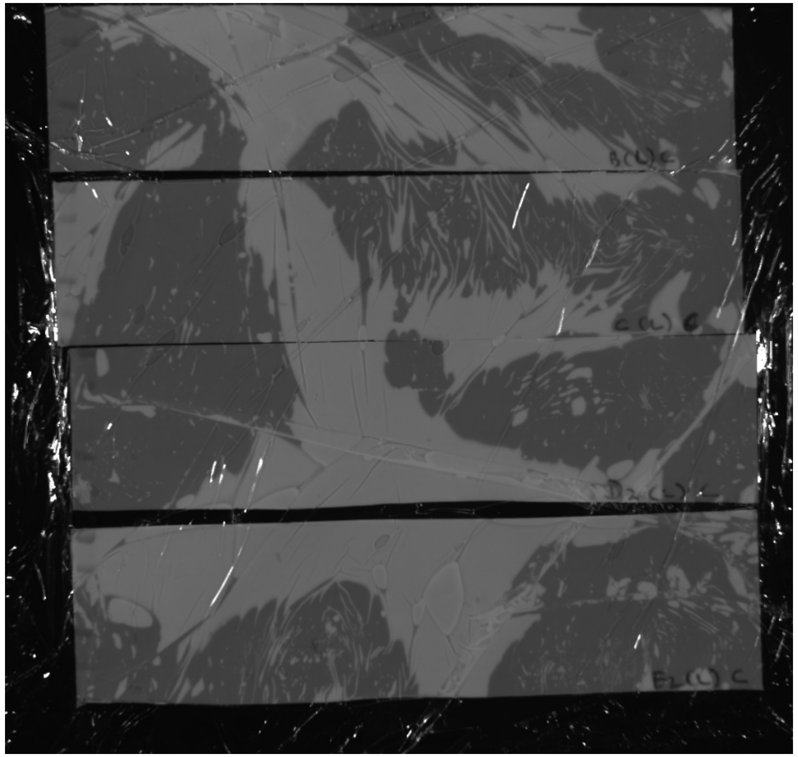

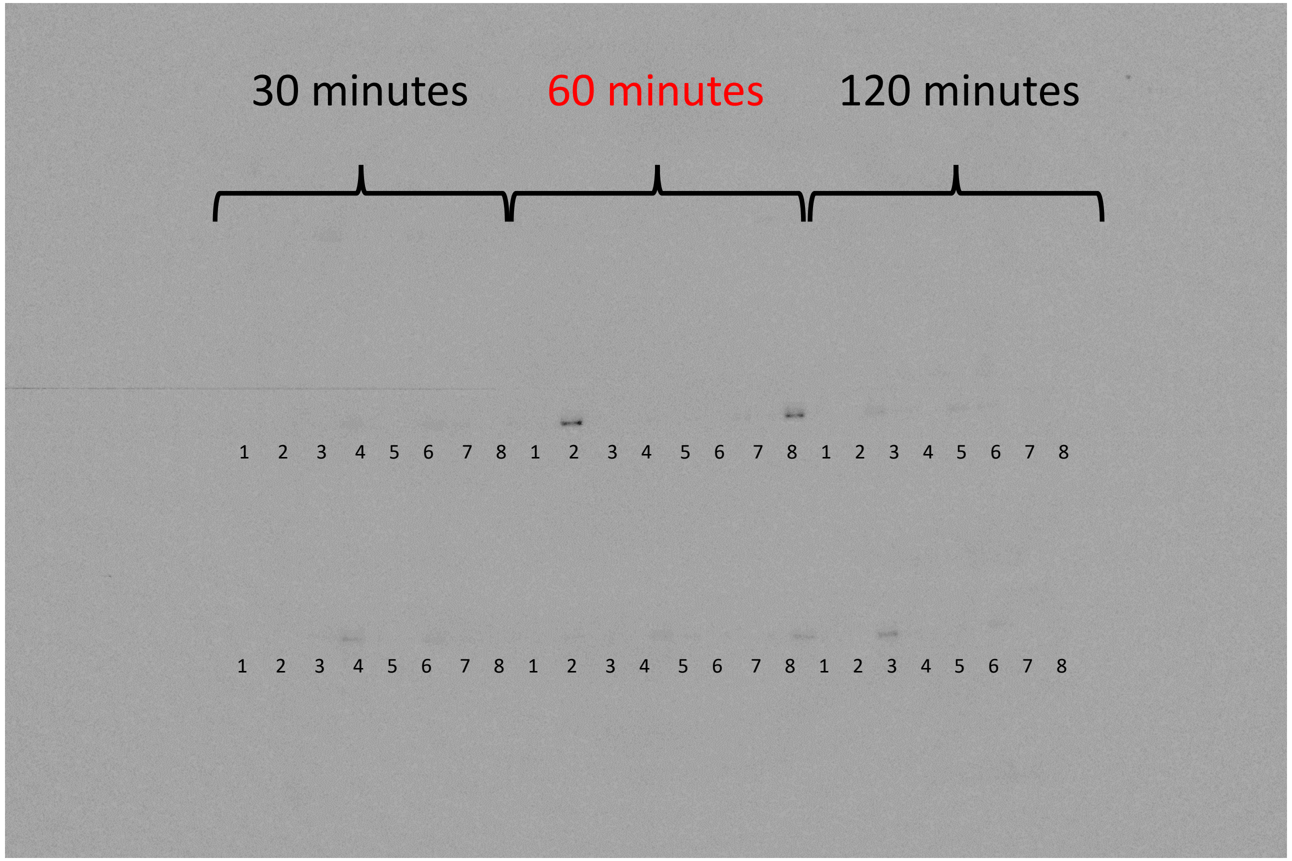

1: Control
2: IL1B
3: P4
4: Forskolin
5: Forskolin + IL1B
6: P4 + IL1B
7: P4 + forskolin
8: P4 + forskolin + IL1B

60-minute time point used

Figure 2C&E TBP (38 kDa) (for Phospho-P65 & phospho-c-jun nuclear - equivalent labelling as per slide 20 to 22 and 24 to 25)

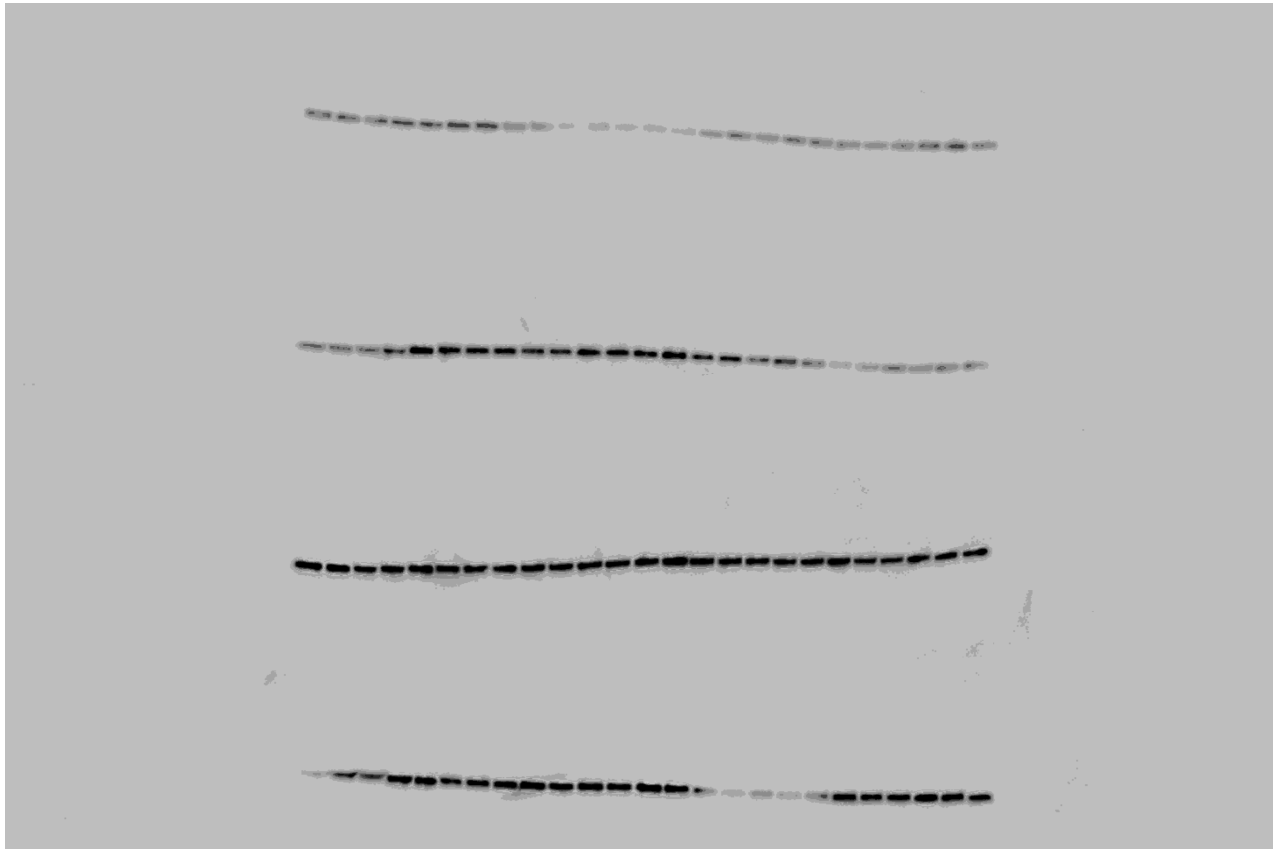

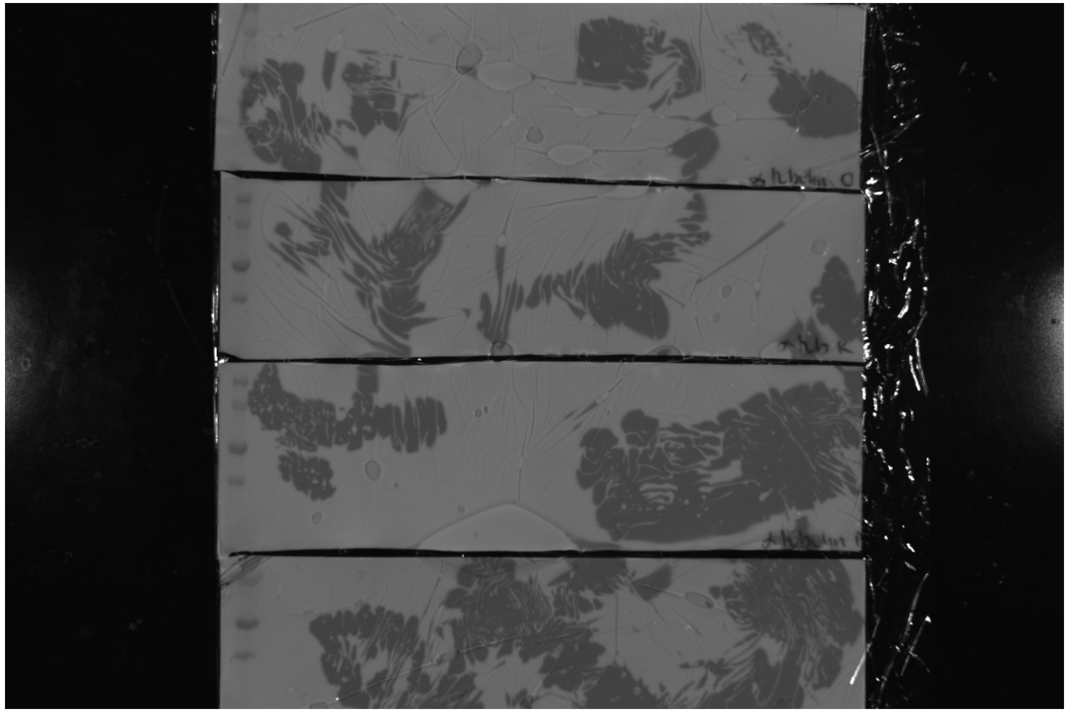

Figure 2C&E TBP (38 kDa) (for Phospho-P65 & phospho-c-jun - nuclear - equivalent labelling as per slide 20 to 22 and 24 to 25)

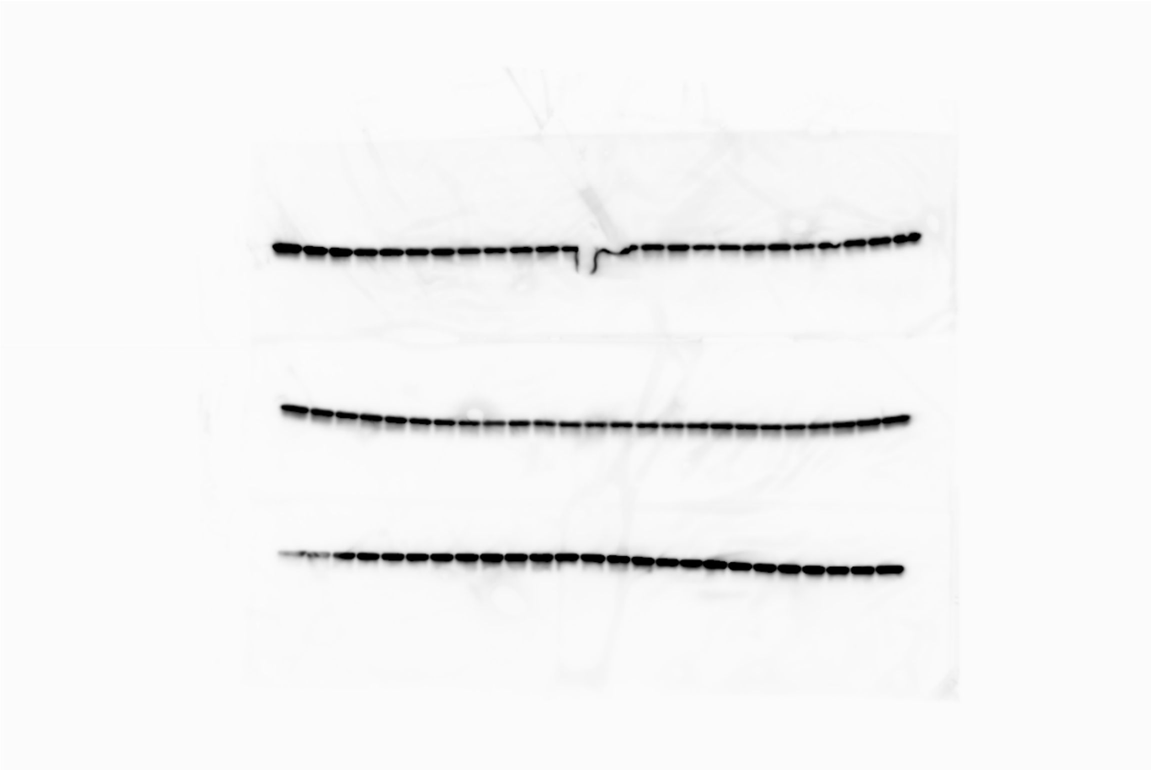

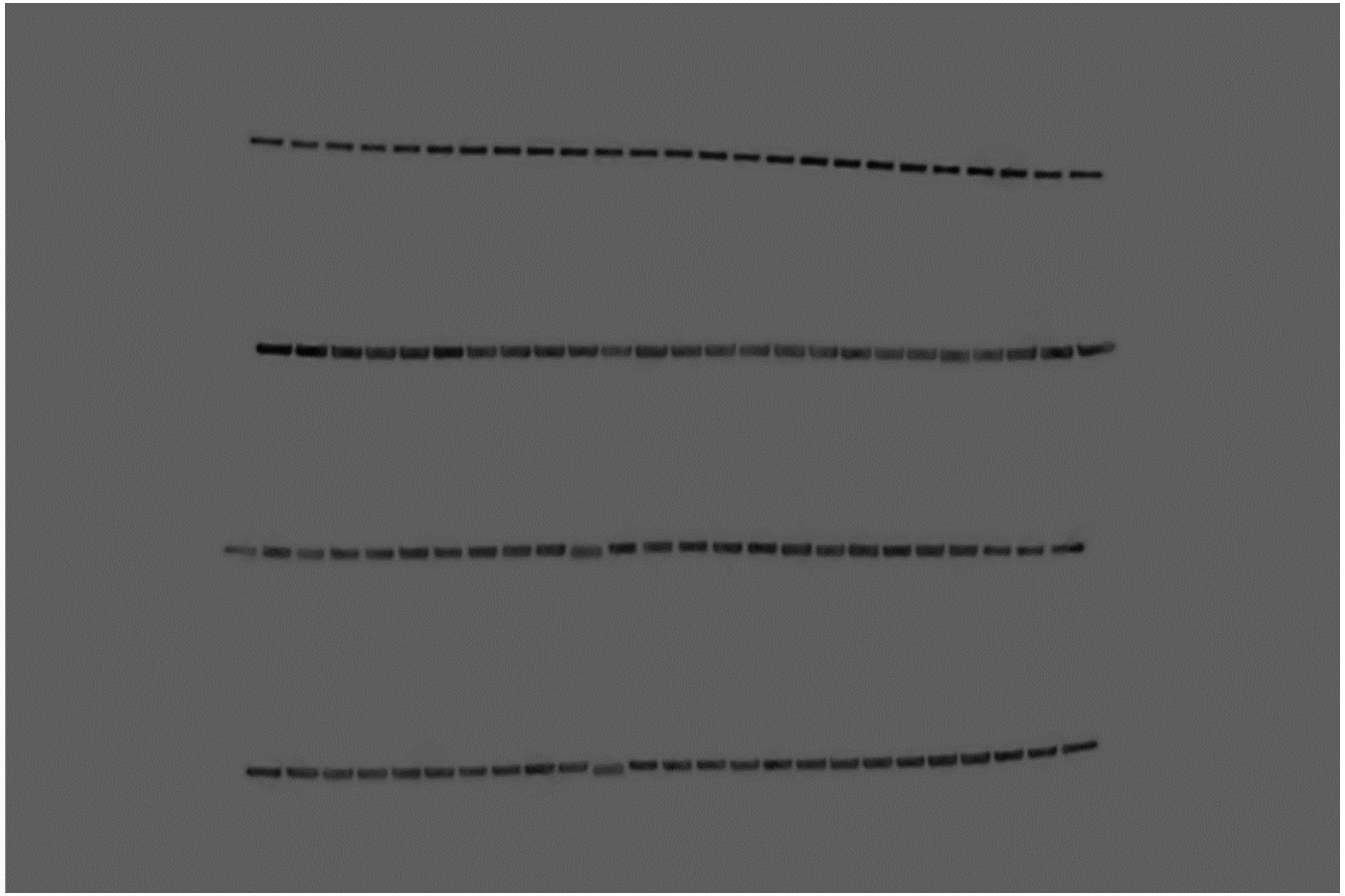

Figure 2D&F α-tubulin (55 kDa) (for Phospho-P65 & phospho-c-jun cytoplasmic - equivalent labelling as per slide 23 and 26 to 27)

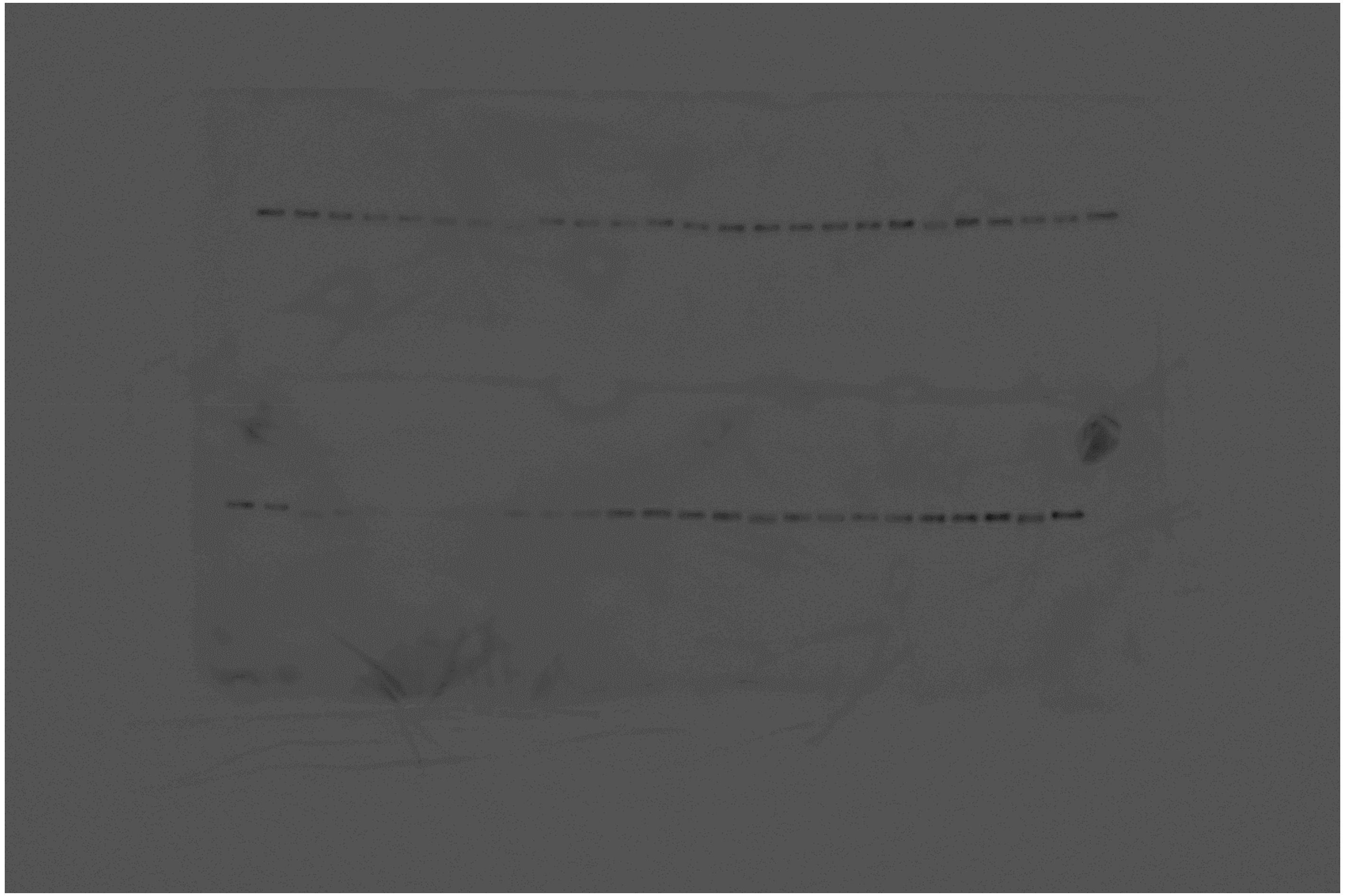

Figure 2D&F α-tubulin (55 kDa) (for Phospho-P65 & phospho-c-jun cytoplasmic - equivalent labelling as per slide 23 and 26 to 27)

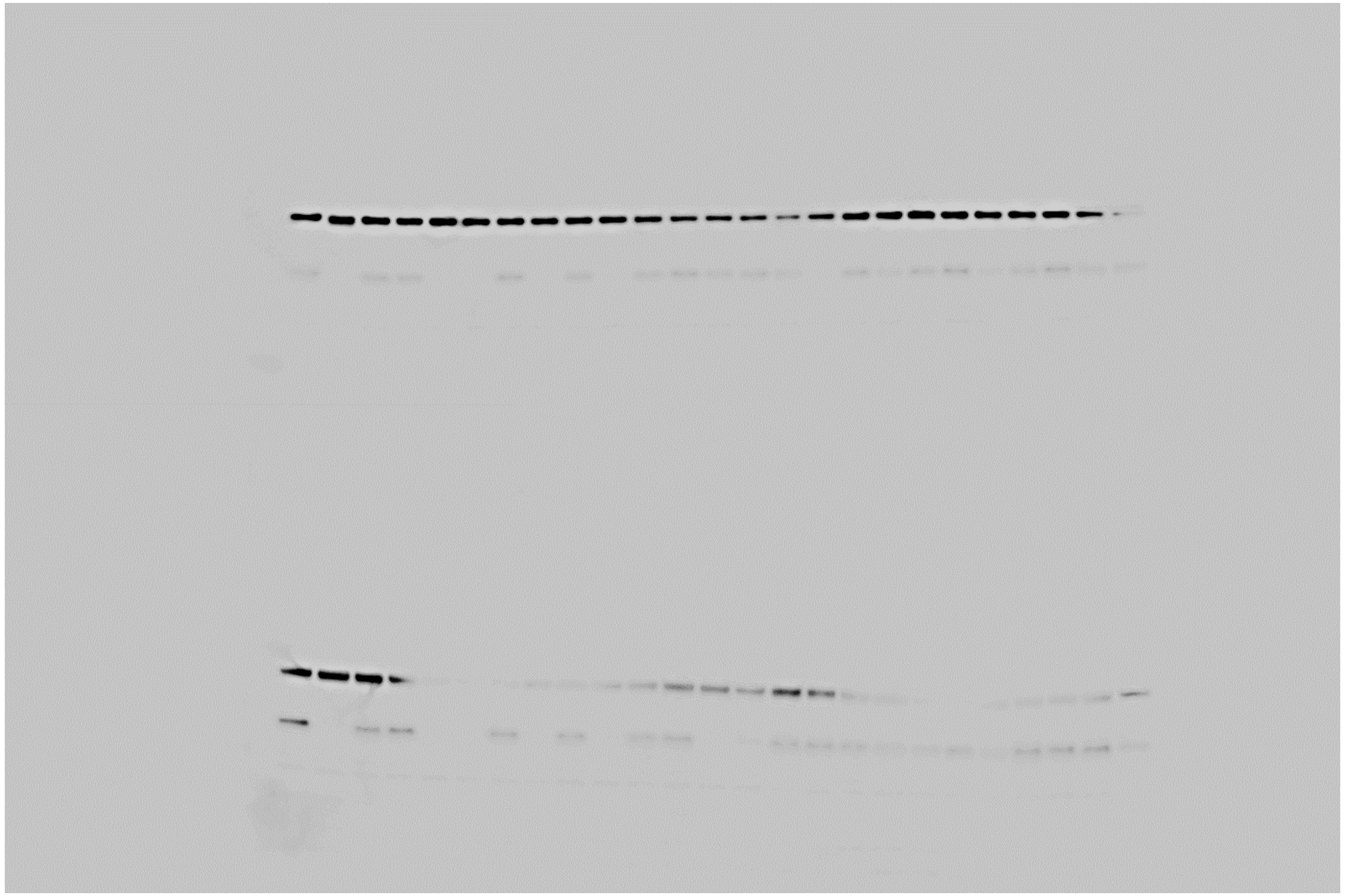

Figure 2H MKP-1 (40 kDa)

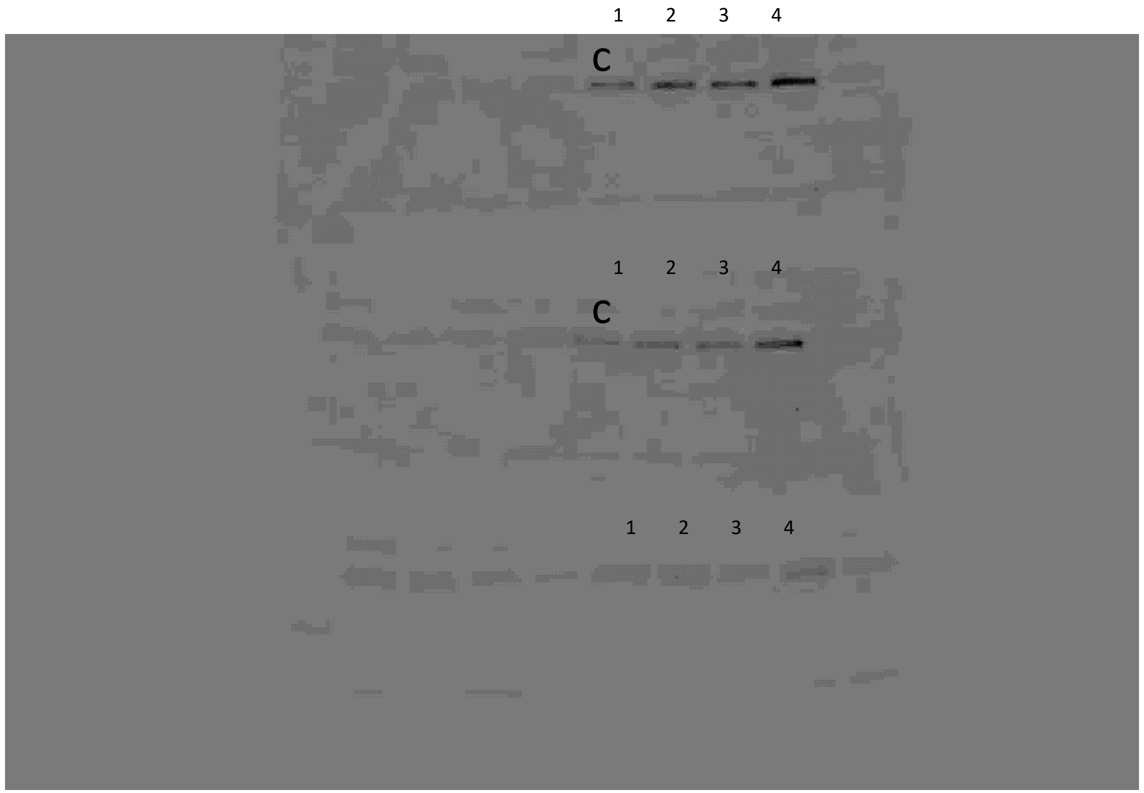

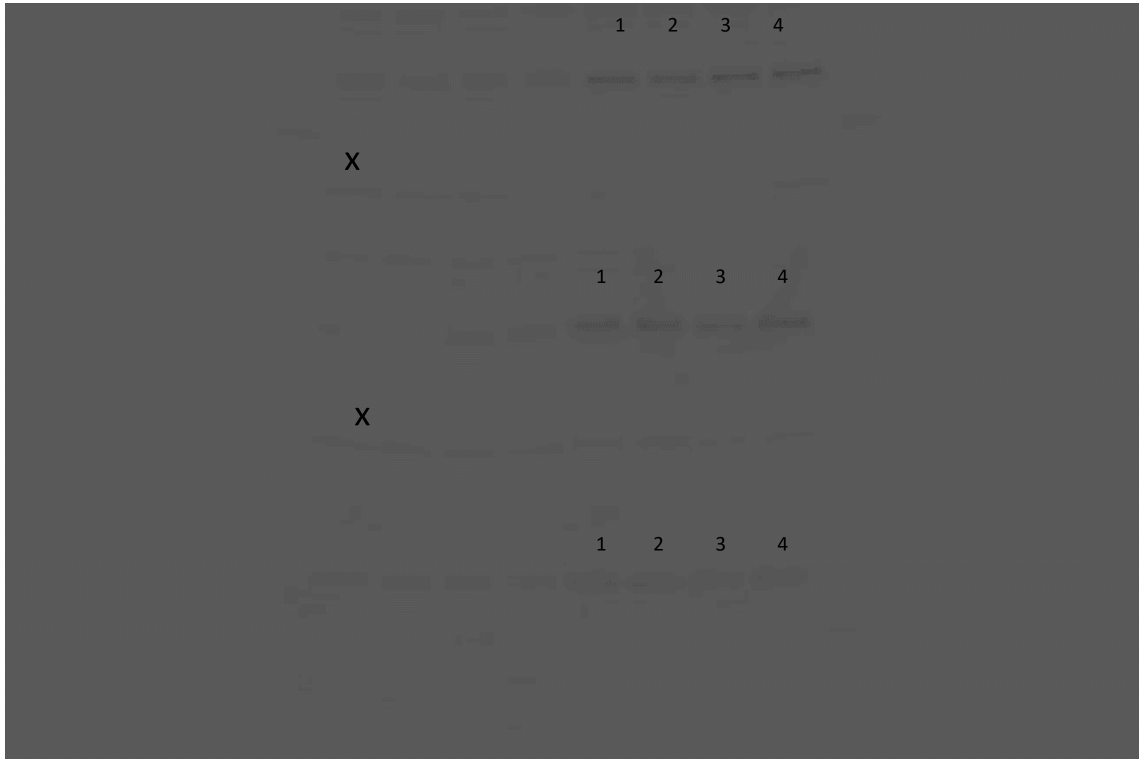

1: Control
2: IL1B
3: Forskolin
5: Forskolin + IL1B

Figure 2H MKP-1 (40 kDa) – additional samples

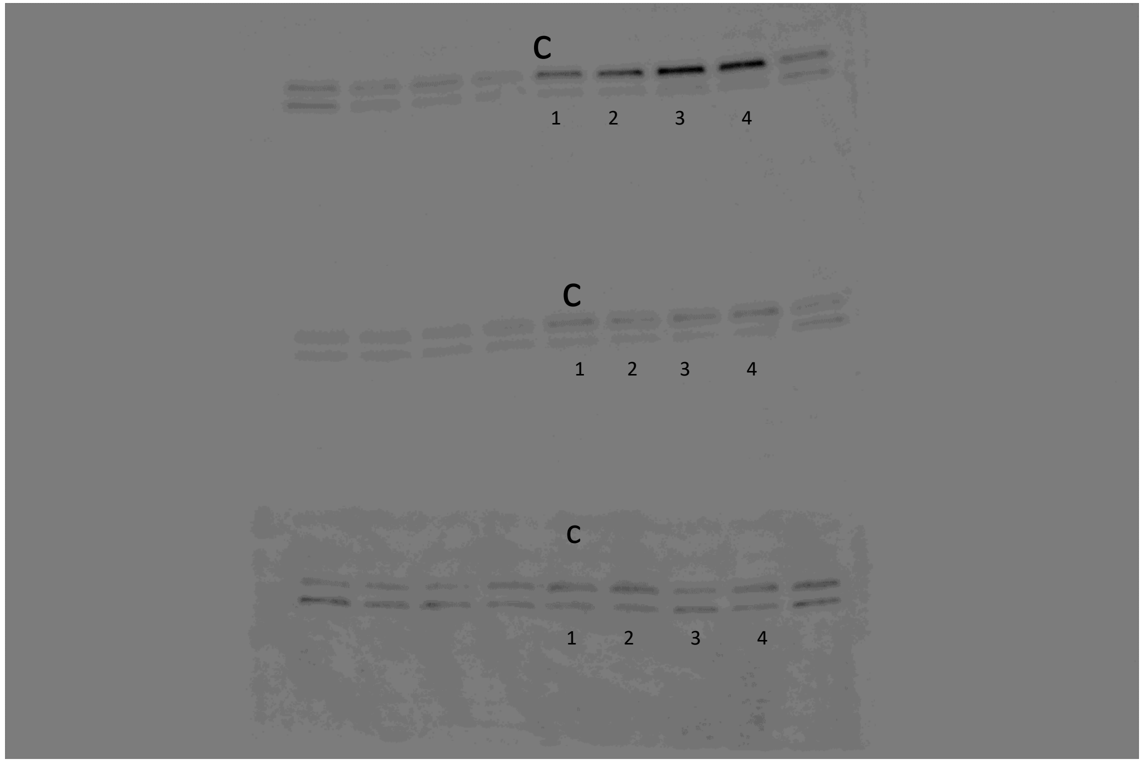

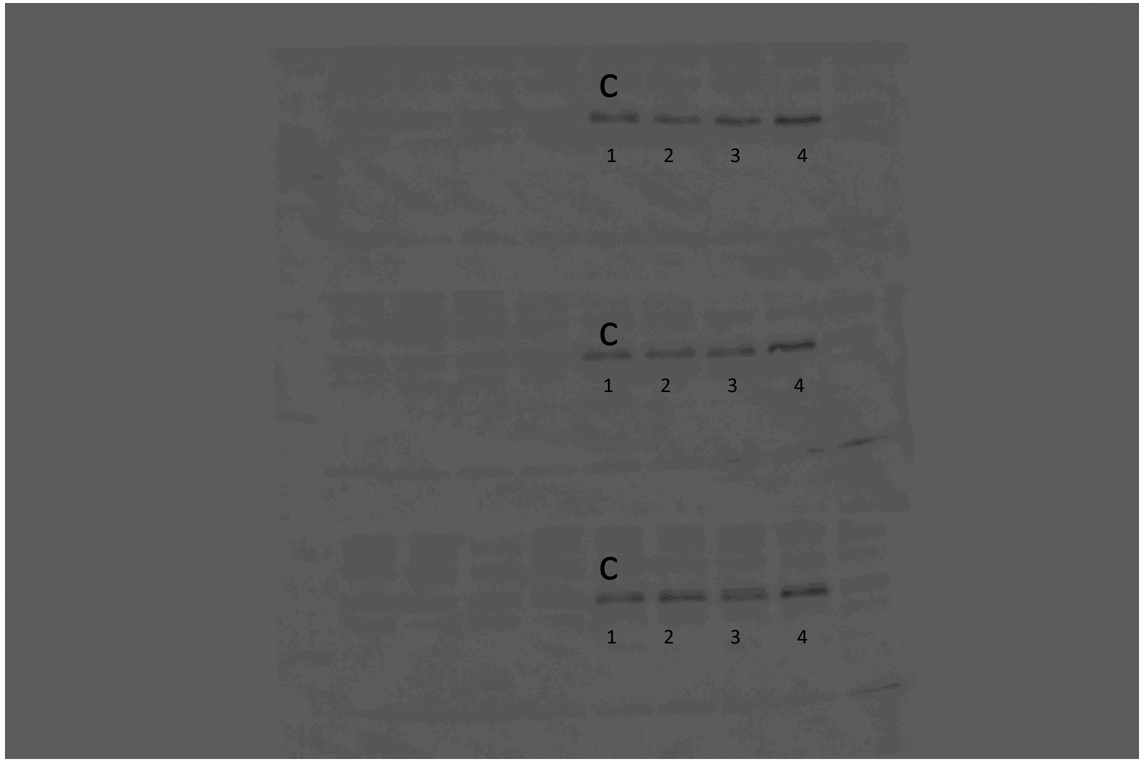

1: Control
2: IL1B
3: Forskolin
5: Forskolin + IL1B

Figure 2H MKP-1 (40 kDa) – additional samples

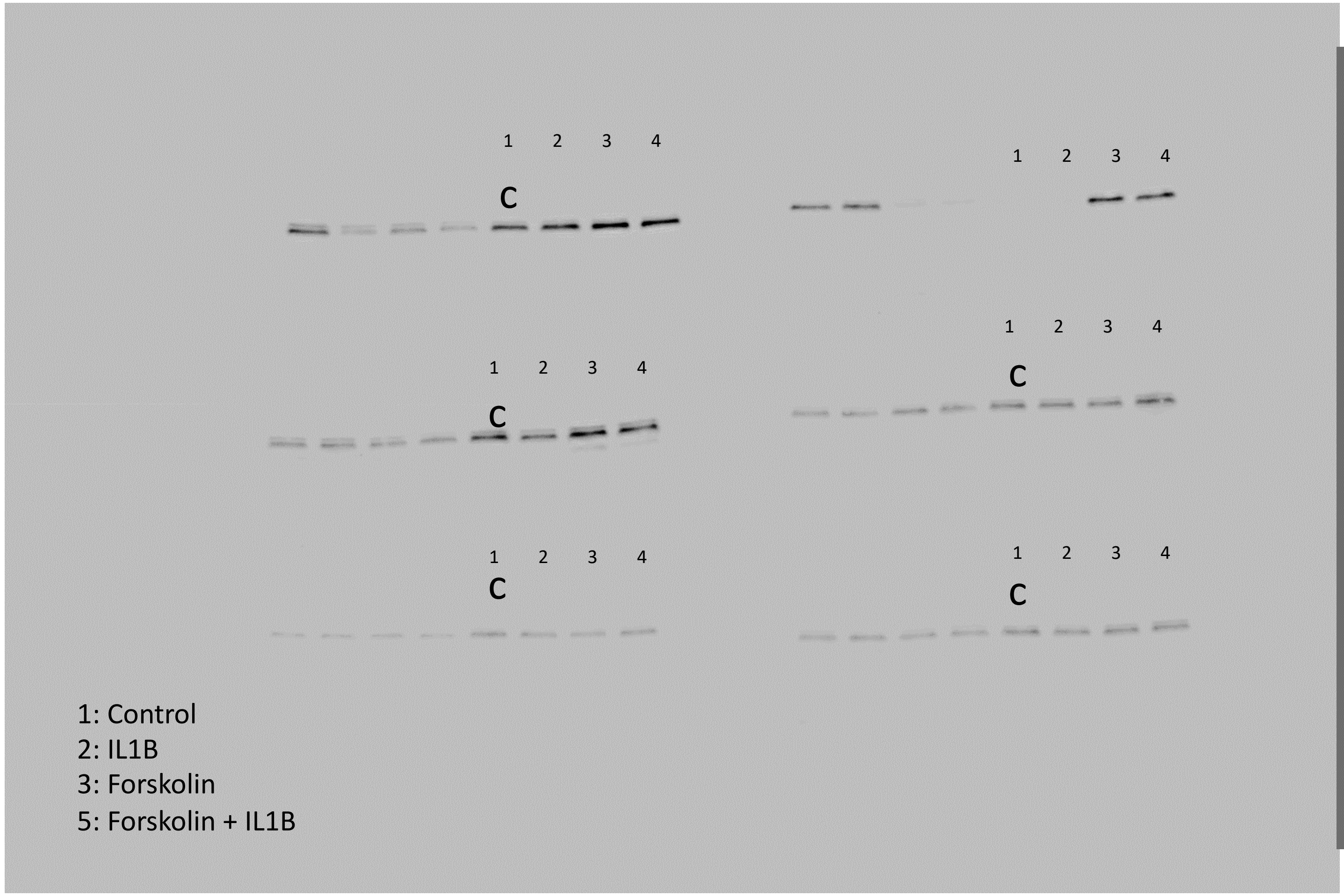

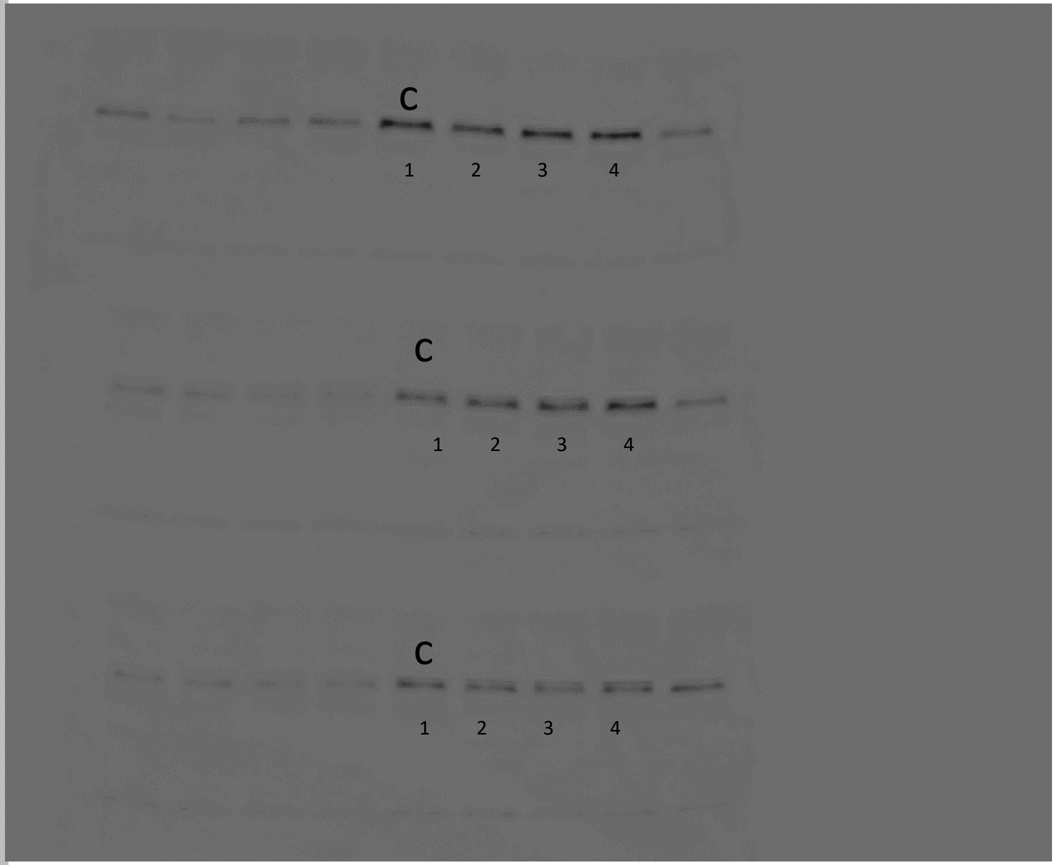

Figure 2J IκBα (39 kDa)

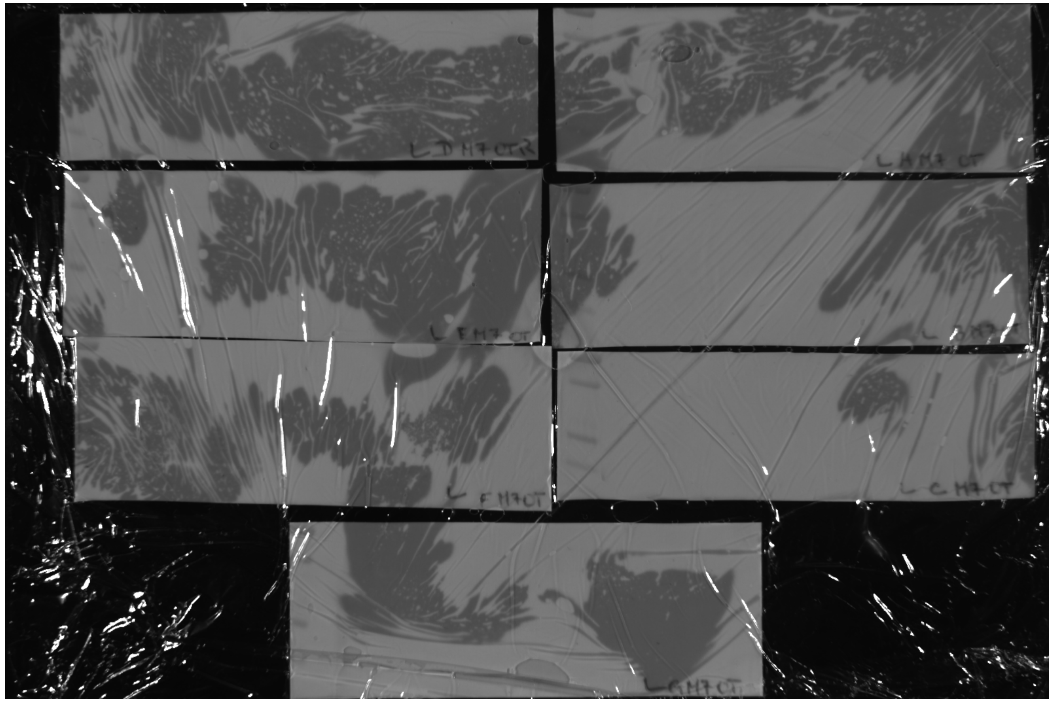

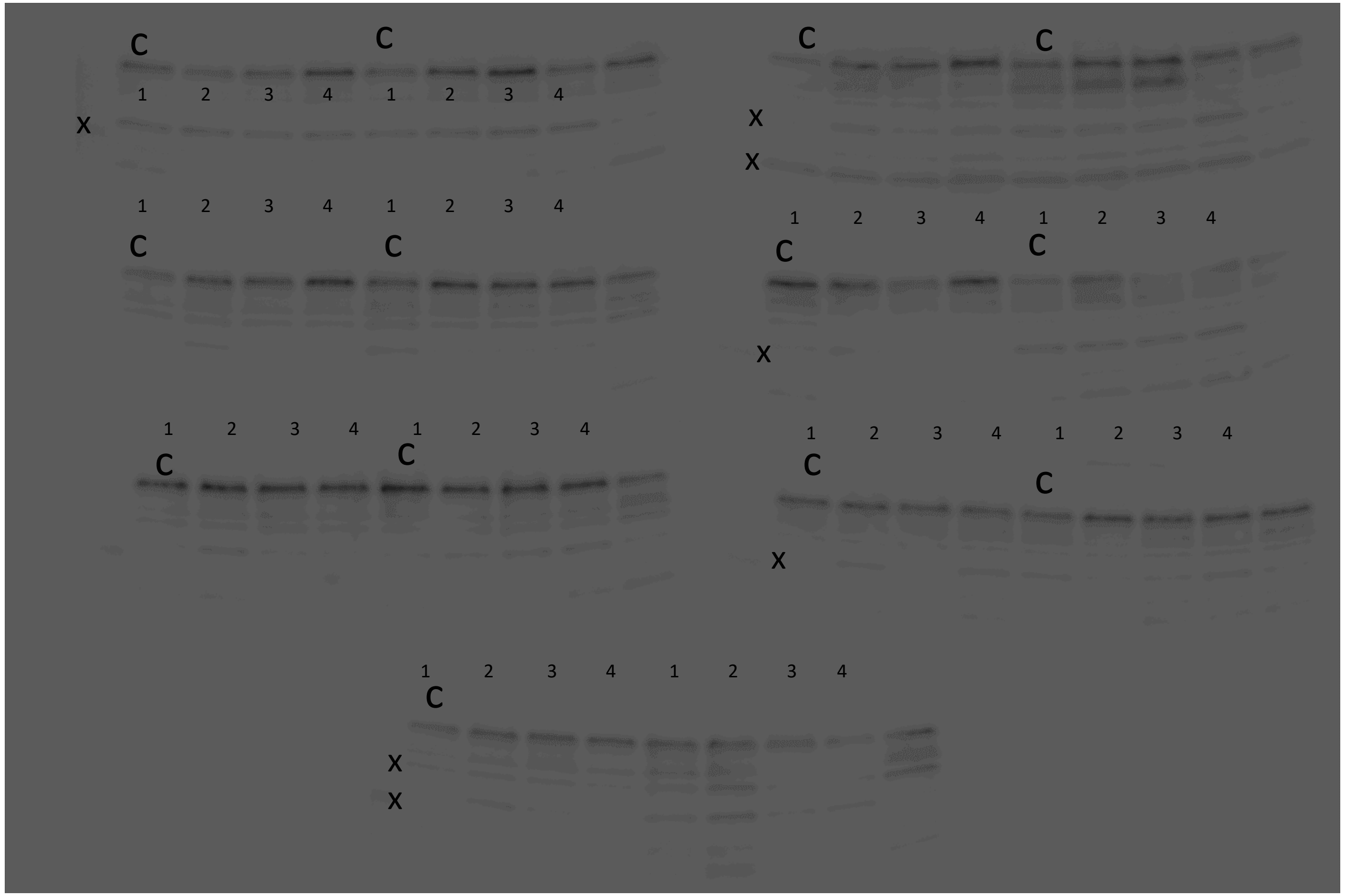

1: Control
2: IL1B
3: Forskolin
4: F + IL1B

Figure 2H&J GAPDH (38 kDa) (for MKP1 & IκBα - equivalent labelling as per slide 32-35)

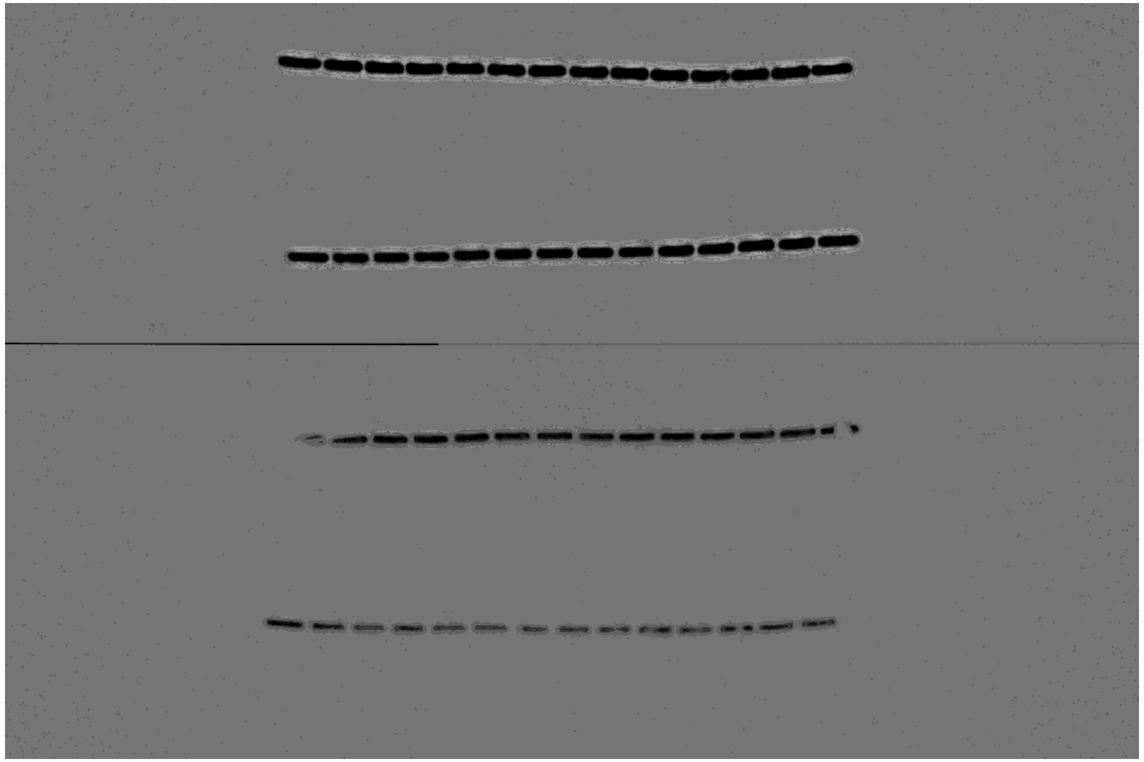

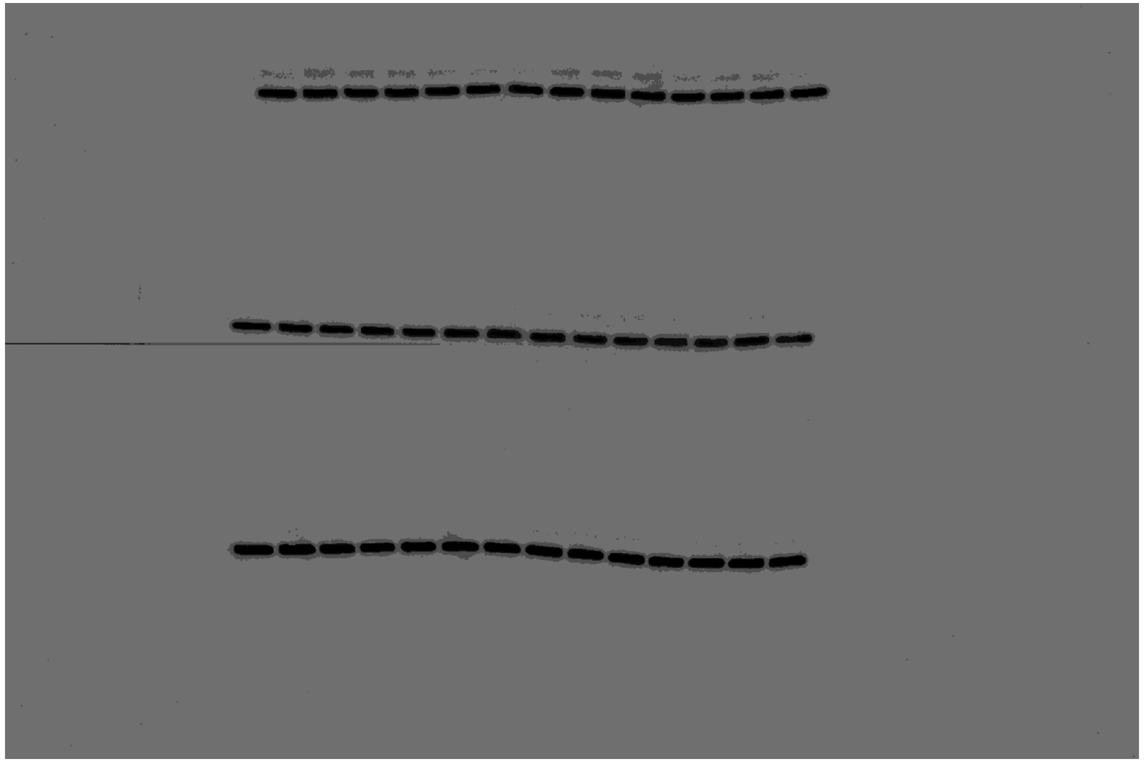

Figure 2H&J GAPDH (38 kDa) (for MKP1 & IκBα - equivalent labelling as per slide 32-35)

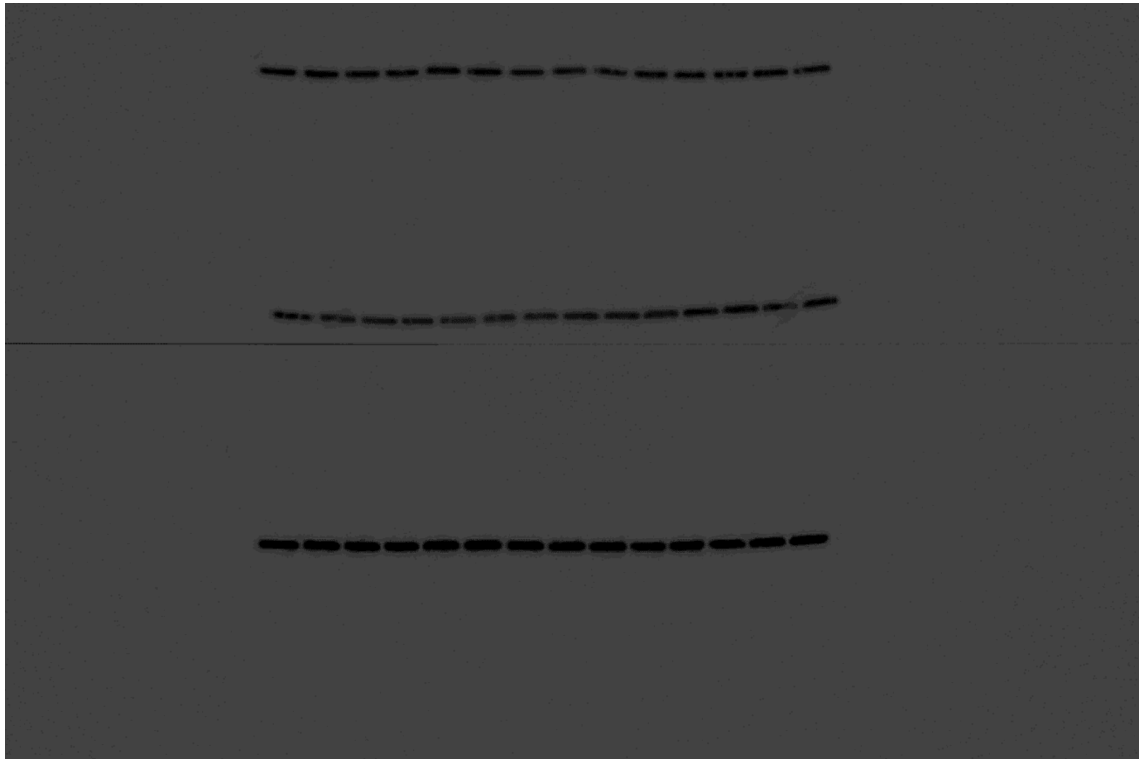

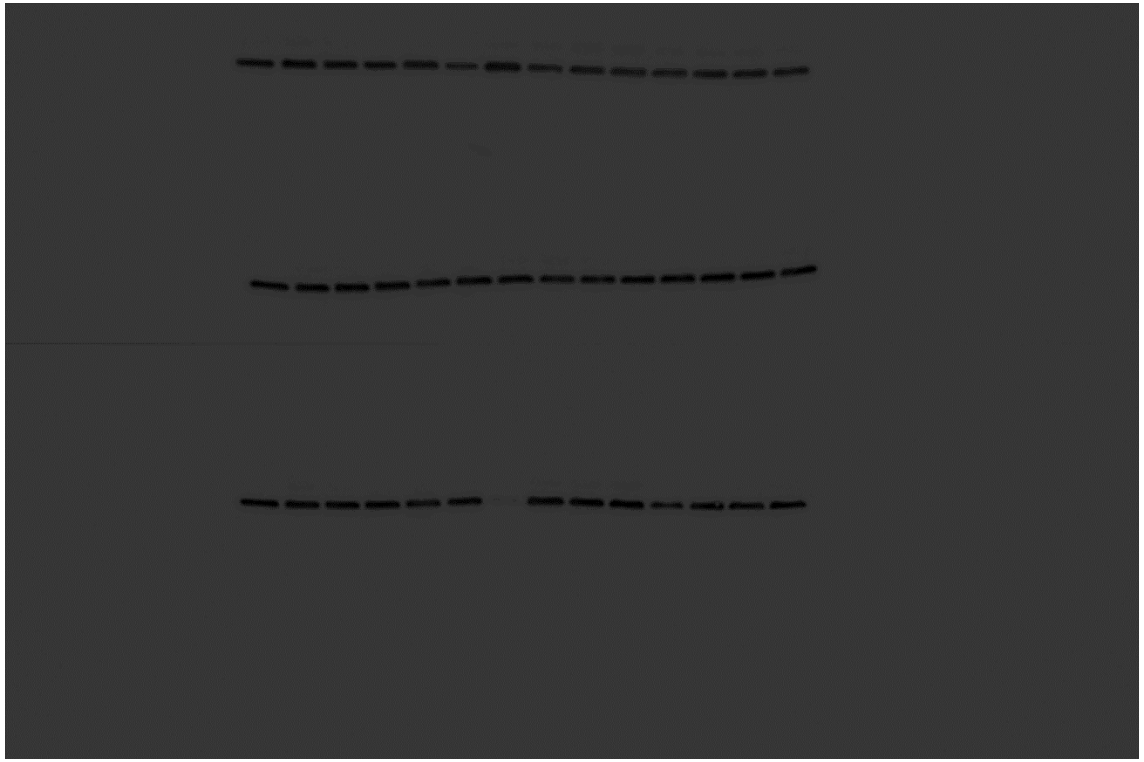

Figure 2H&J GAPDH (38 kDa) (for MKP1 & IκBα - equivalent labelling as per slide 32-35)

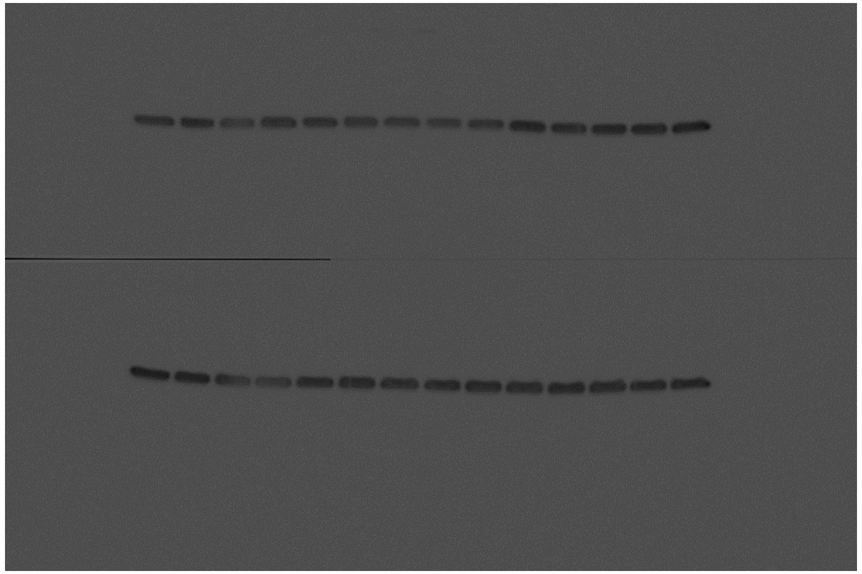

Figure 3B OTR (66 kDa)

1: Control
2: IL1B
3: P4
4: Forskolin
5: P4 + IL1B
6: F + IL1B
7: P4 + forskolin + IL1B

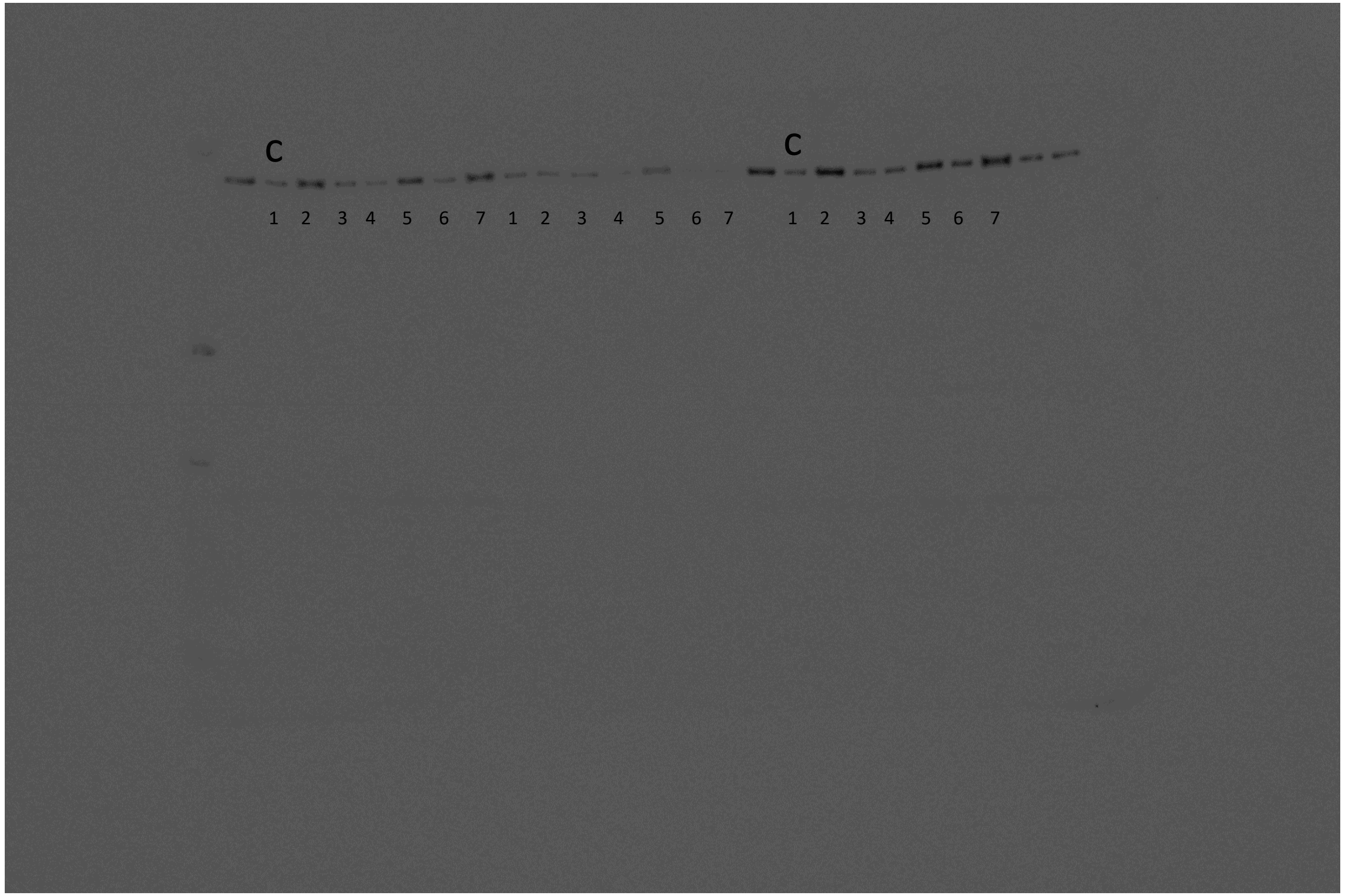

Figure 3B OTR (66 kDa) – additional samples

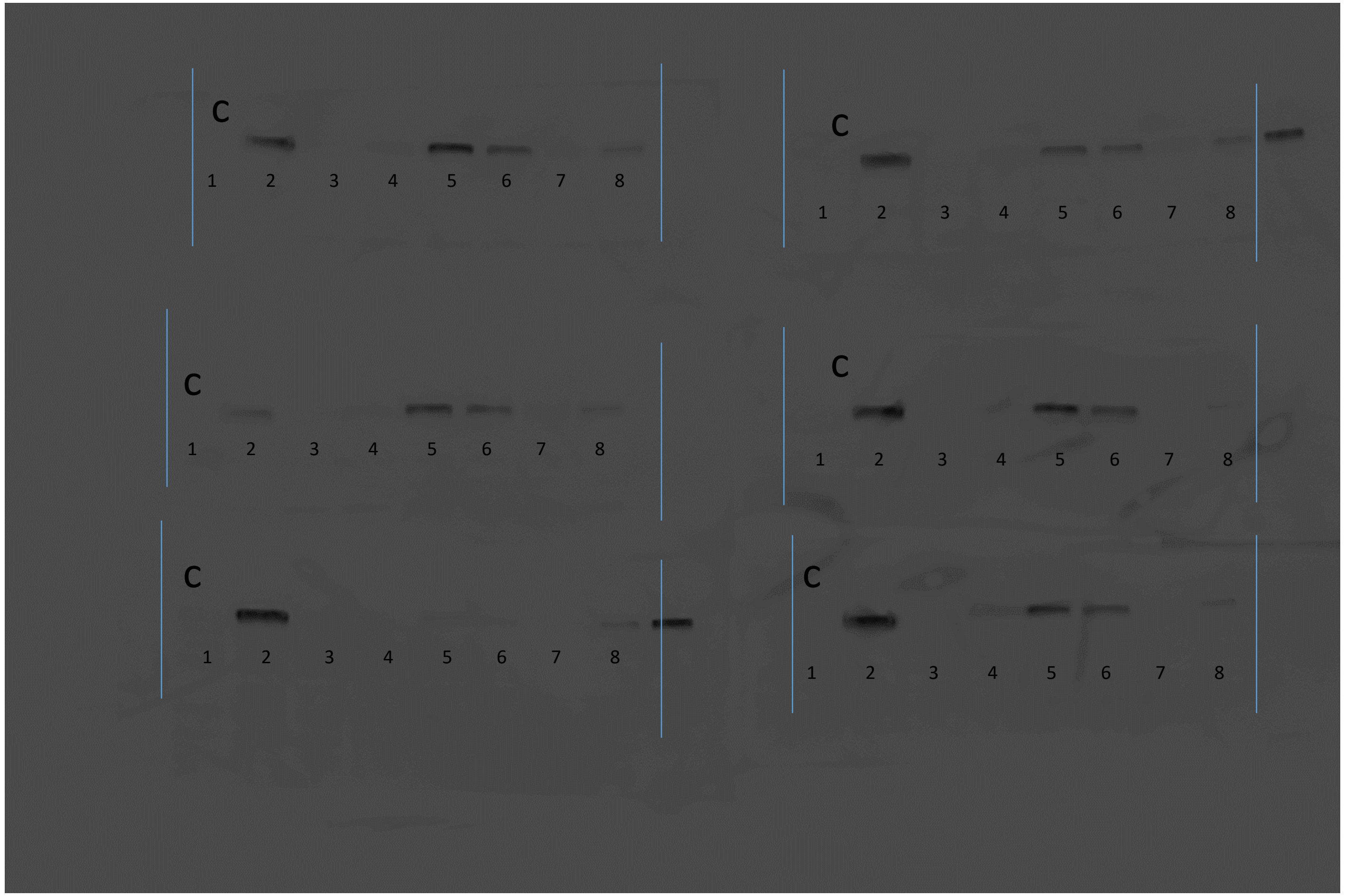

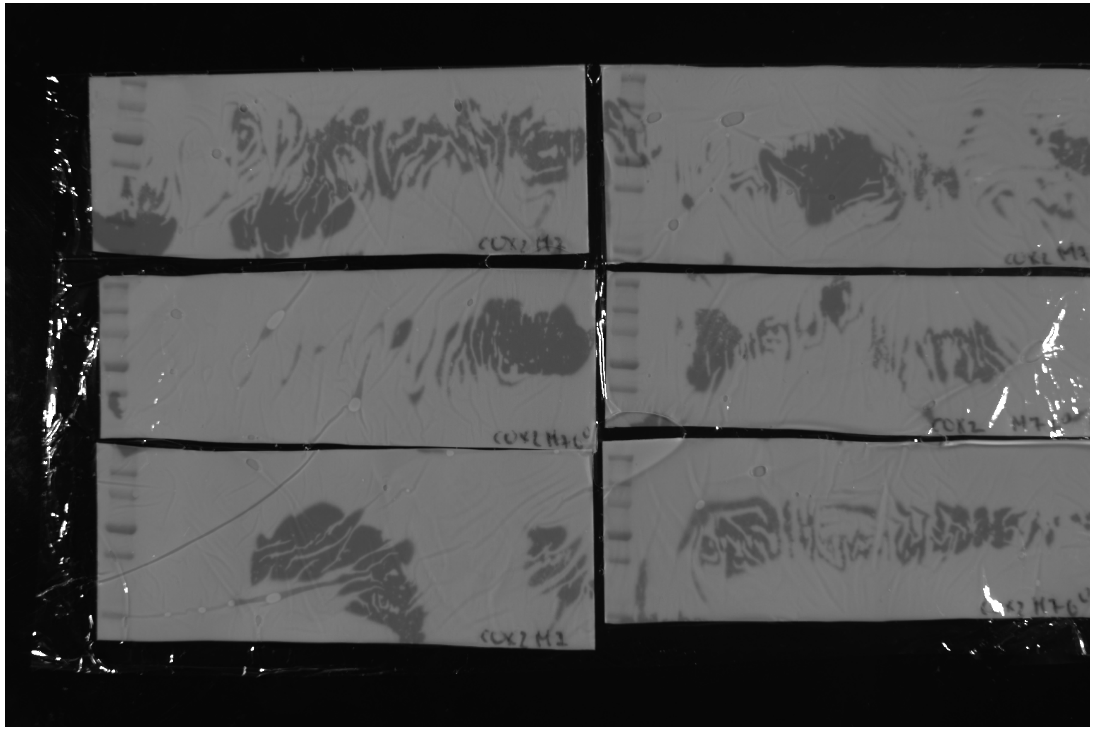

1: Control
2: IL1B
3: P4
4: Forskolin
5: P4 + IL1B
6: F + IL1B
7: P4 + forskolin
8: P4 + forskolin + IL1B

Figure 3B GAPDH (38 kDa) (for OTR - equivalent labelling as per slide 39 to 40)

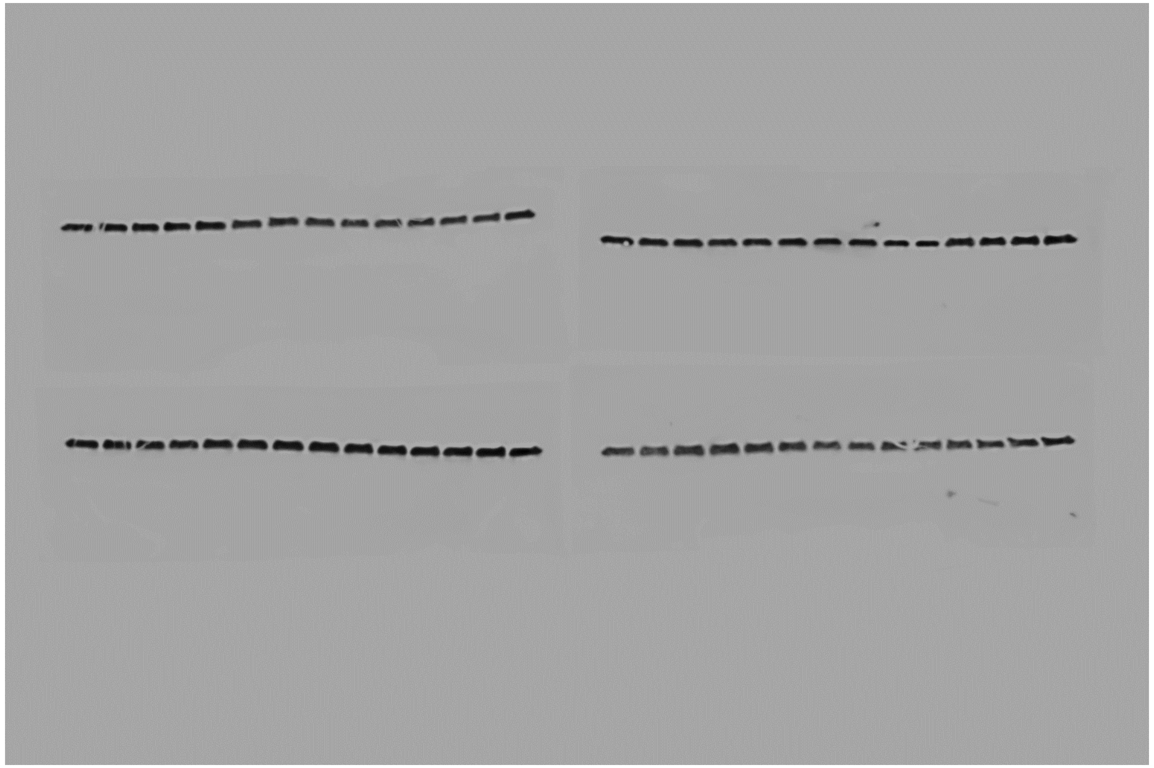

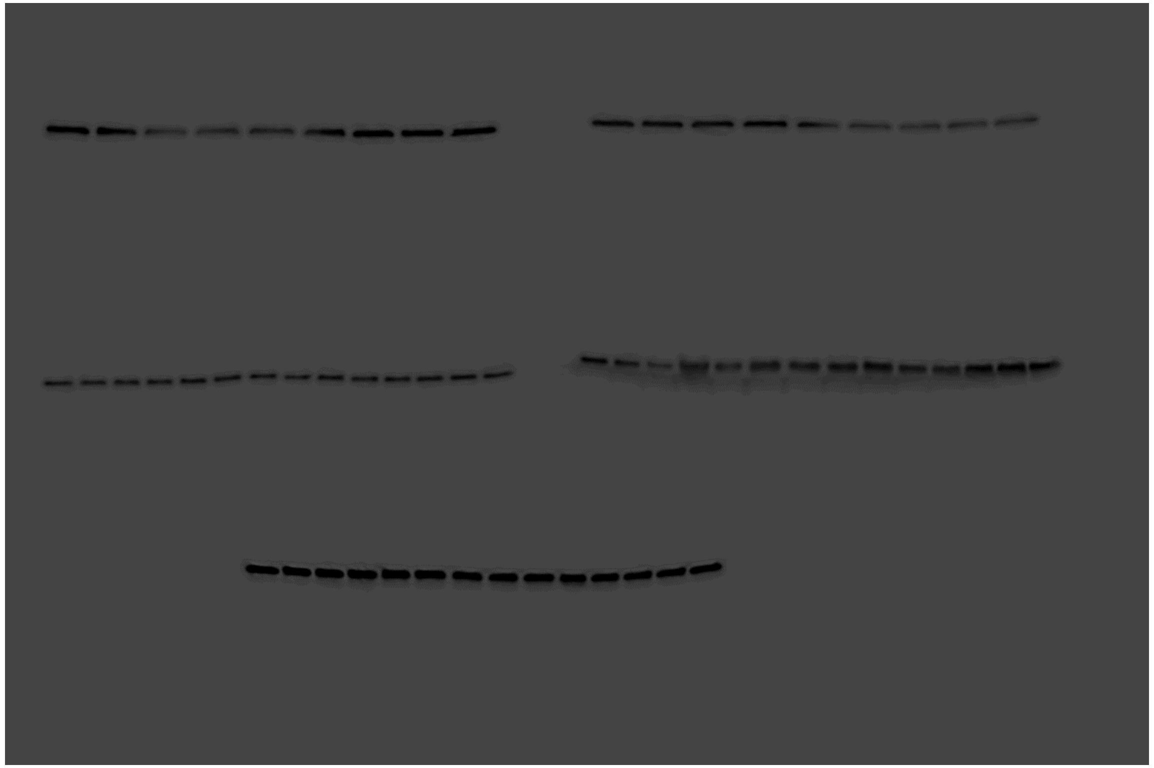

Figure 3B GAPDH (38 kDa) (for OTR - equivalent labelling as per slide 39 to 40)

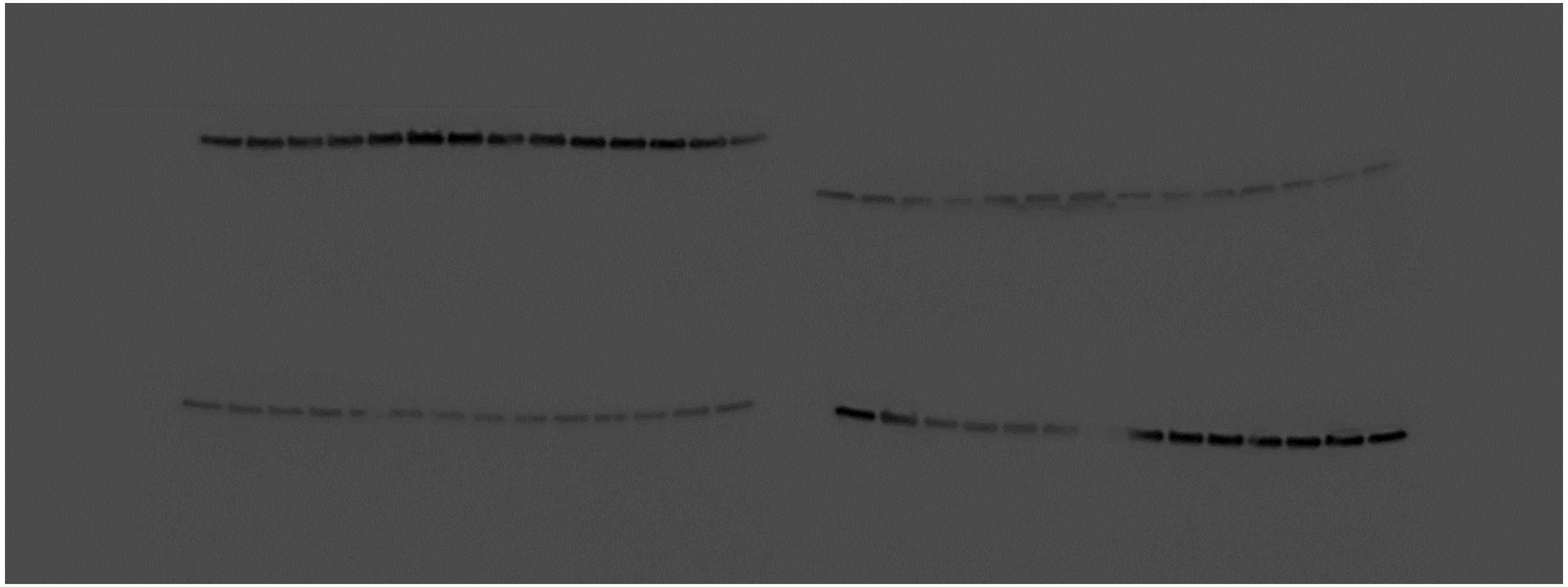

Supplementary Figure 6A Phospho-JNK (46-54 kDa)

1: Control
2: IL1B
3: P4
4: Forskolin
5: P4 + IL1B
6: F + IL1B
7: Forskolin + P4
8: P4 + forskolin + IL1B

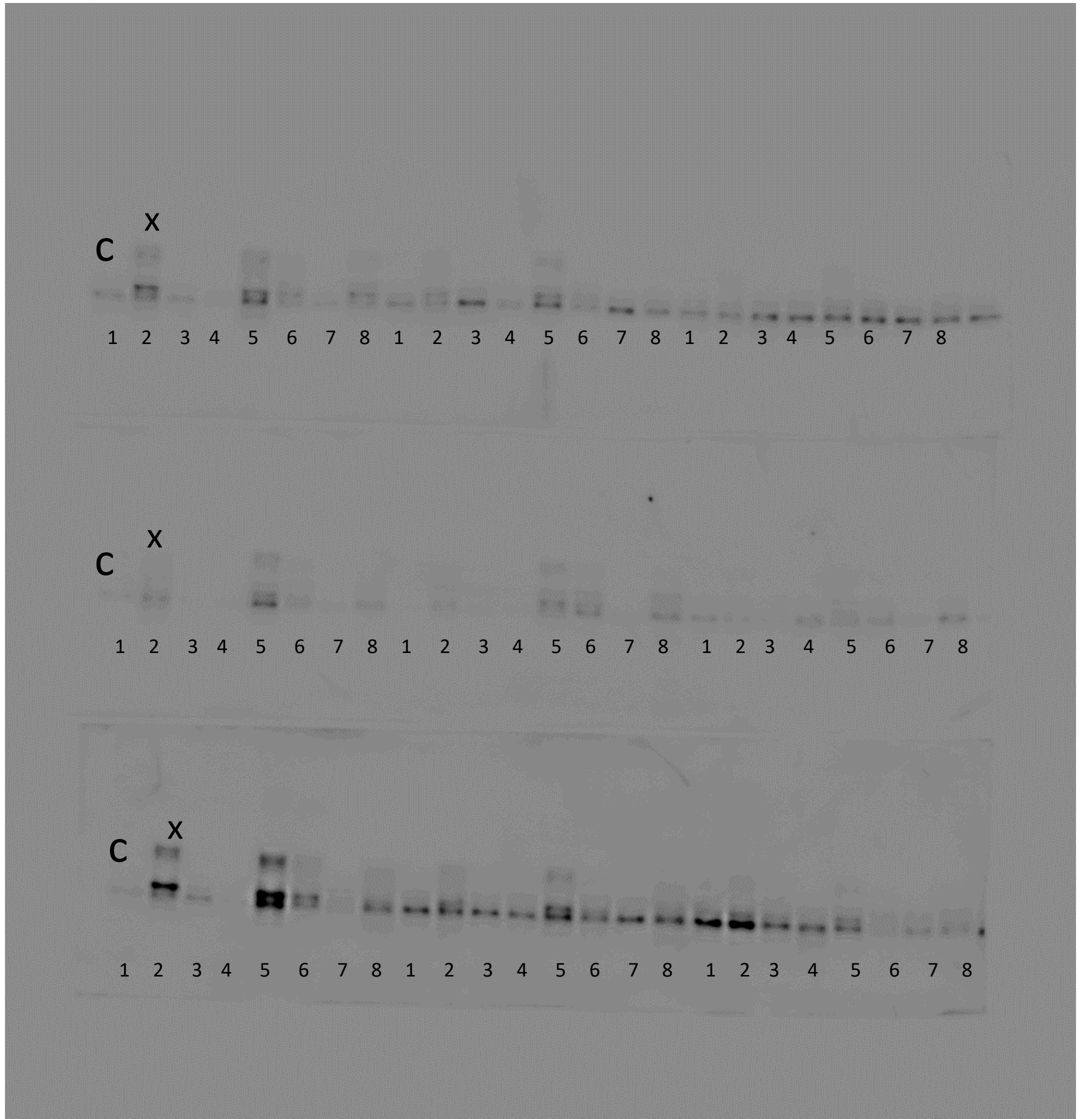

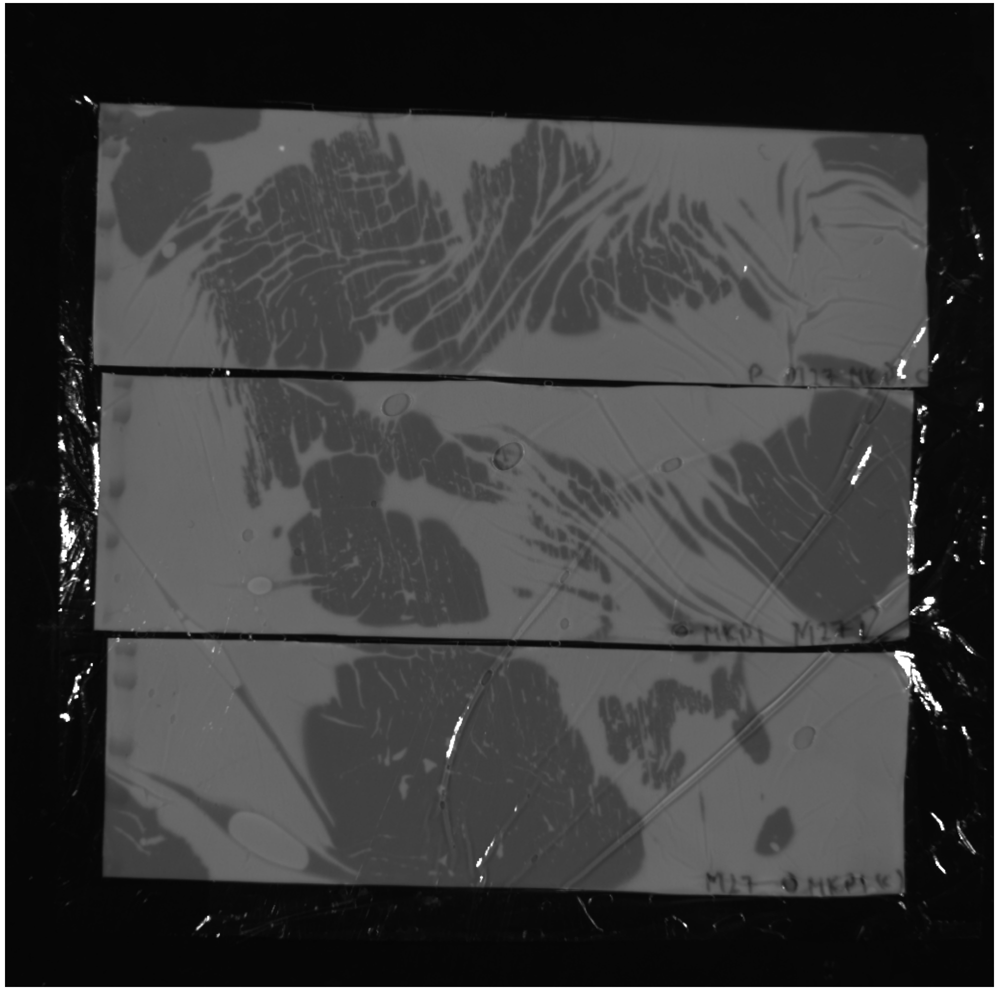

Supplementary Figure 6A Phospho-JNK (46-54 kDa) – additional samples

1: Control
2: IL1B
3: P4
4: Forskolin
5: P4 + IL1B
6: F + IL1B
7: Forskolin + P4
8: P4 + forskolin + IL1B

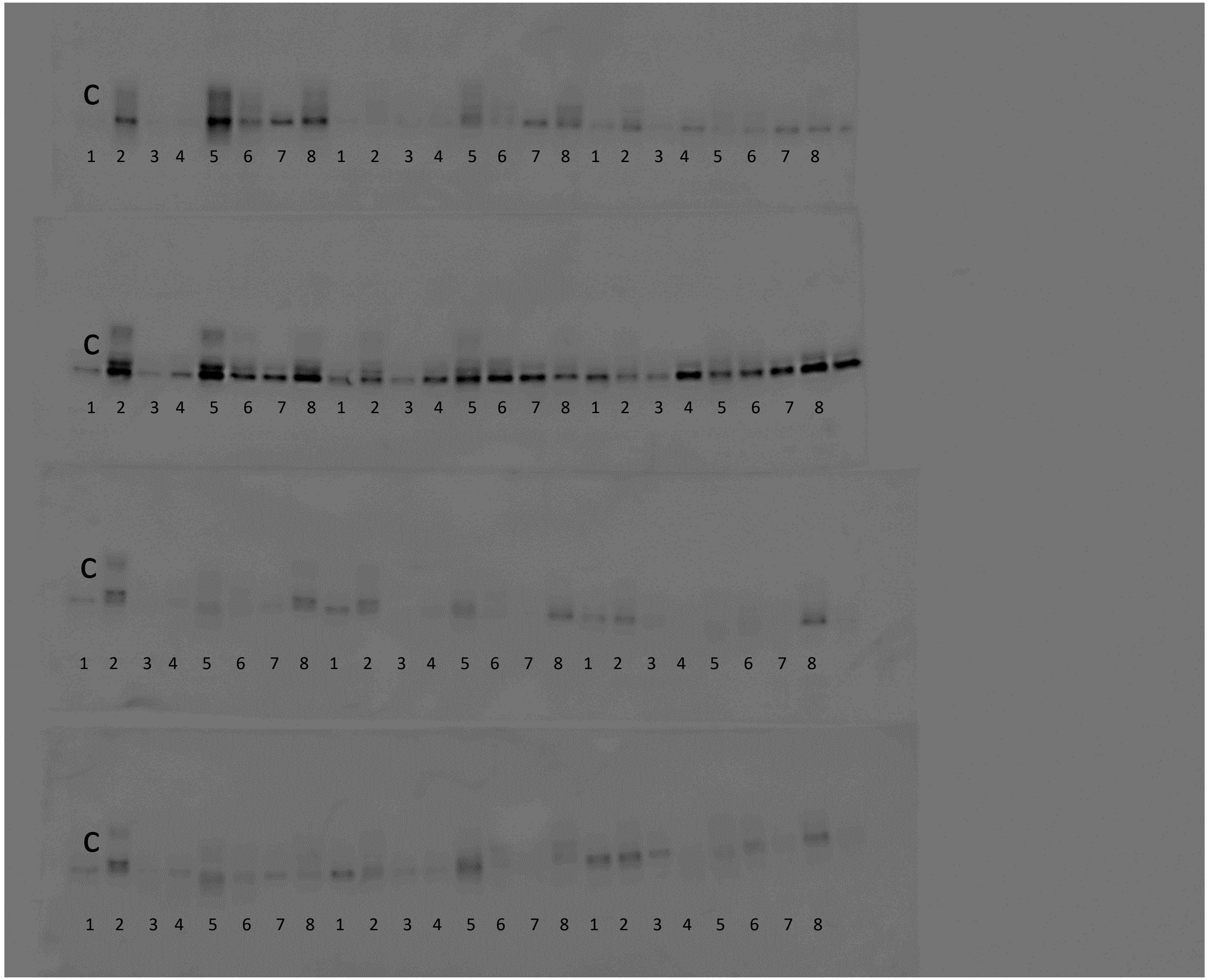

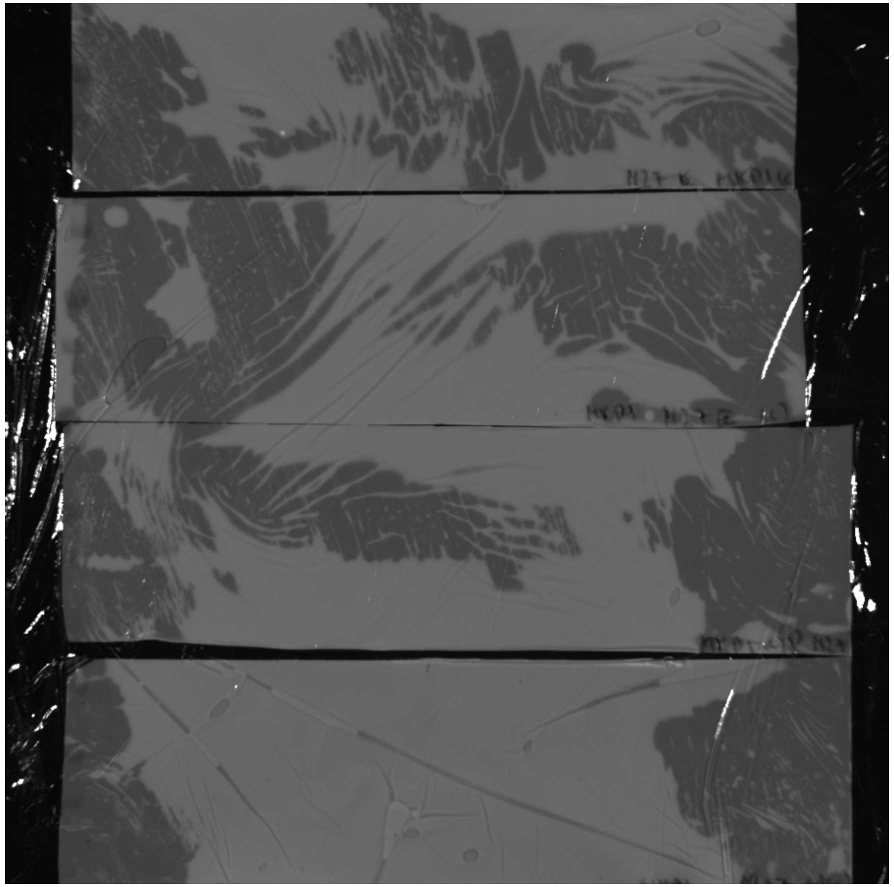

Supplementary Figure 6B Phospho-p38 (43 kDa)

1: Control
2: IL1B
3: P4
4: Forskolin
5: P4 + IL1B
6: F + IL1B
7: Forskolin + P4
8: P4 + forskolin + IL1B

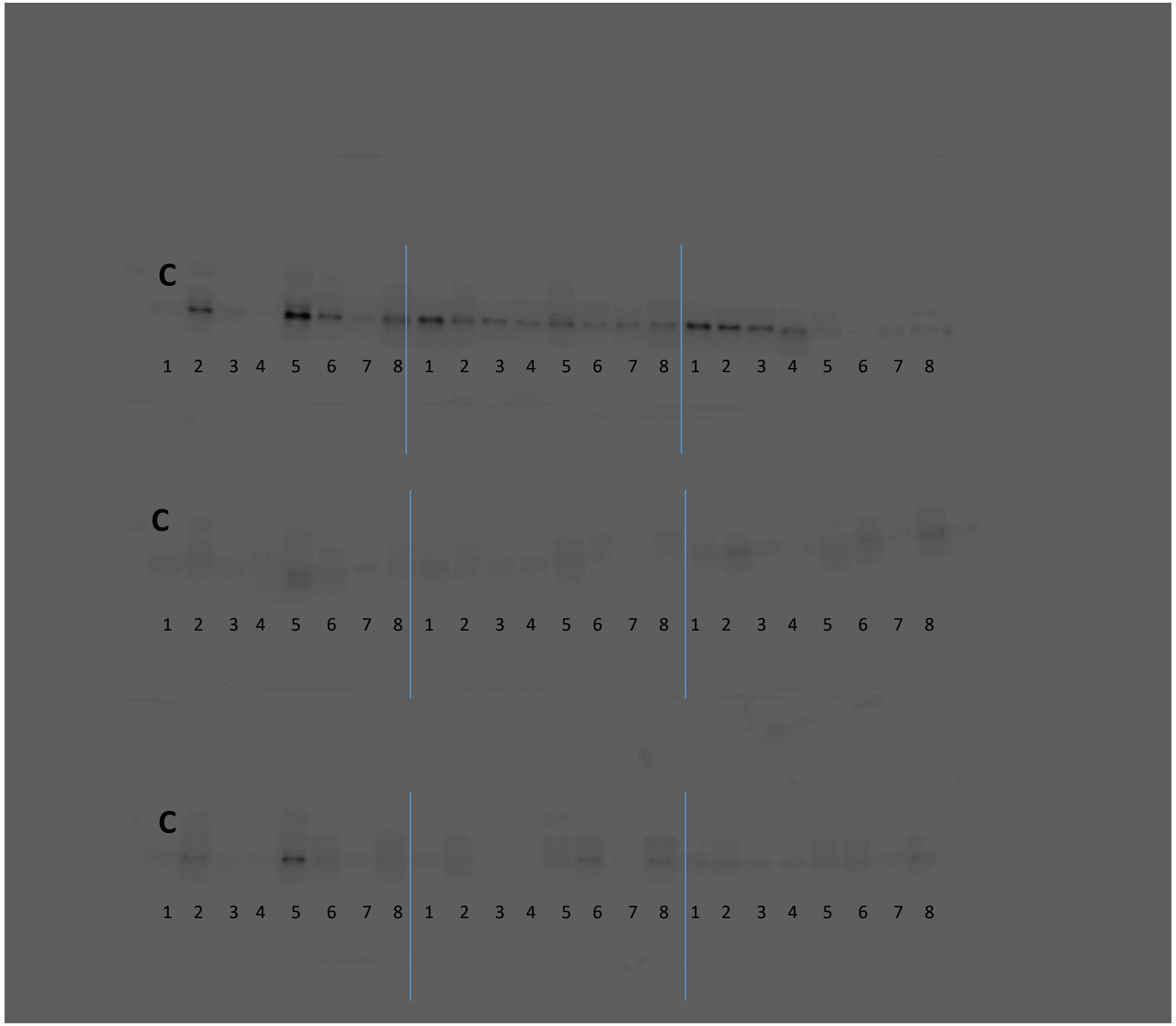

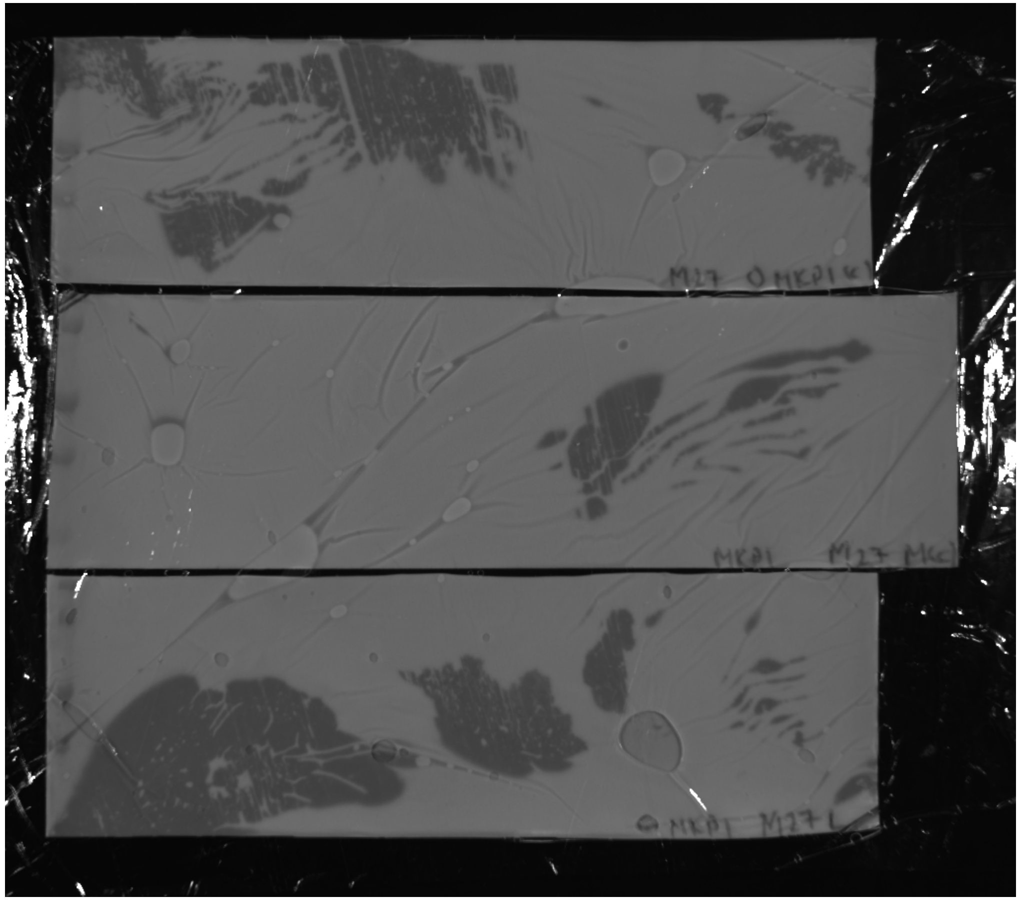

Supplementary Figure 6B Phospho-p38 (43 kDa) – additional samples

1: Control
2: IL1B
3: P4
4: Forskolin
5: P4 + IL1B
6: F + IL1B
7: Forskolin + P4
8: P4 + forskolin + IL1B

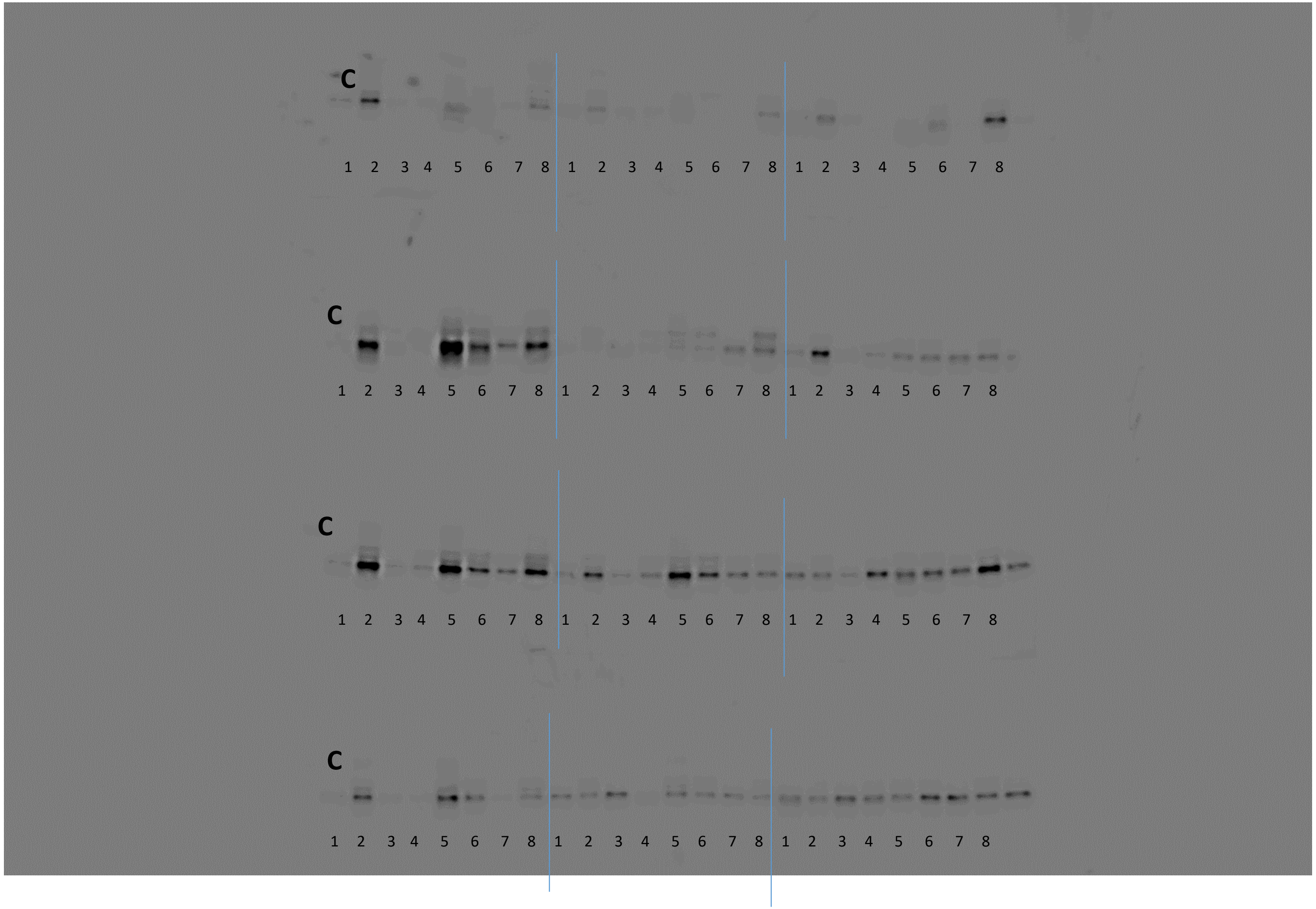

Supplementary Figure 7A MKP1 cytosolic (40 kDa)

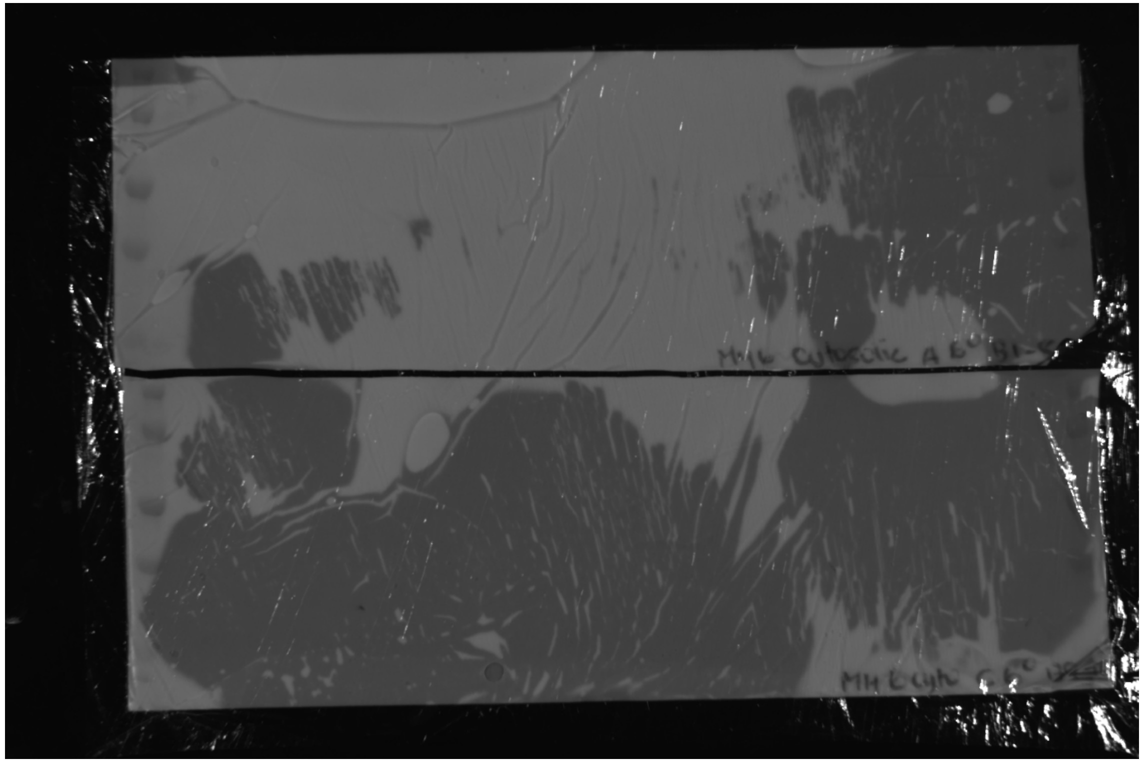

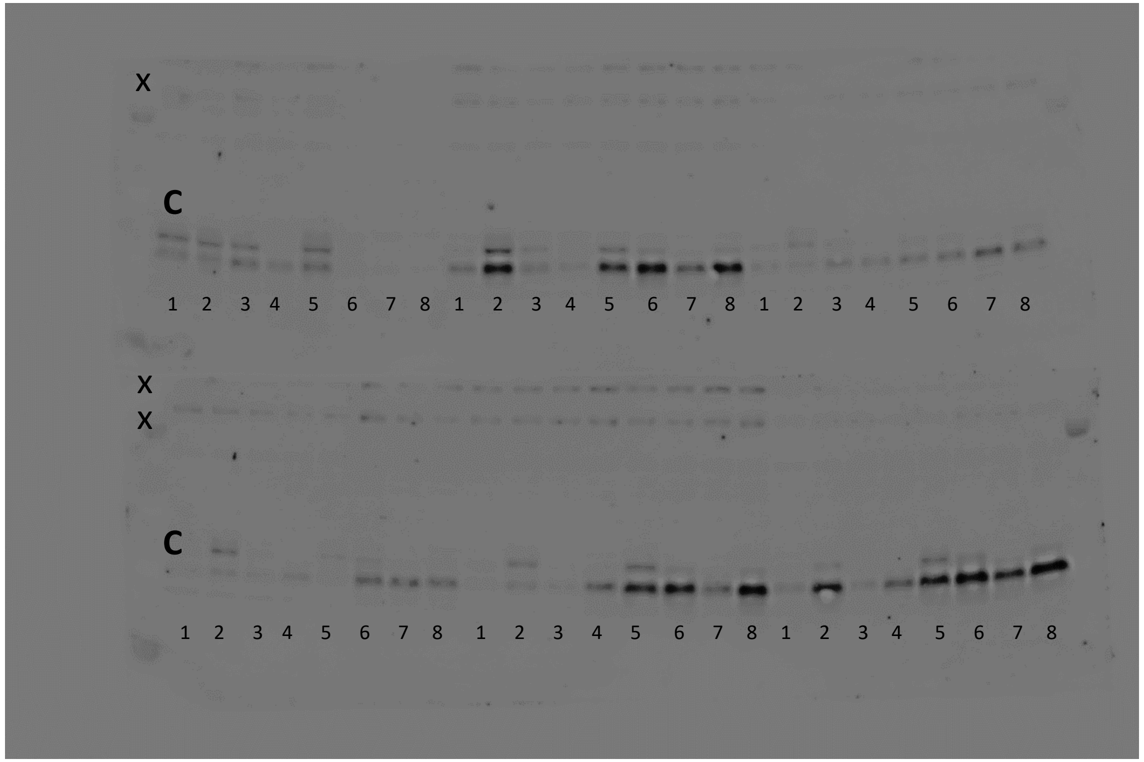

1: Control
2: IL1B
3: P4
4: Forskolin
5: F + IL1B
6: P4 + IL1B
7: P4 + F
8: P4 + forskolin + IL1B

Supplementary Figure 7A MKP1 cytosolic (40 kDa) – additional samples

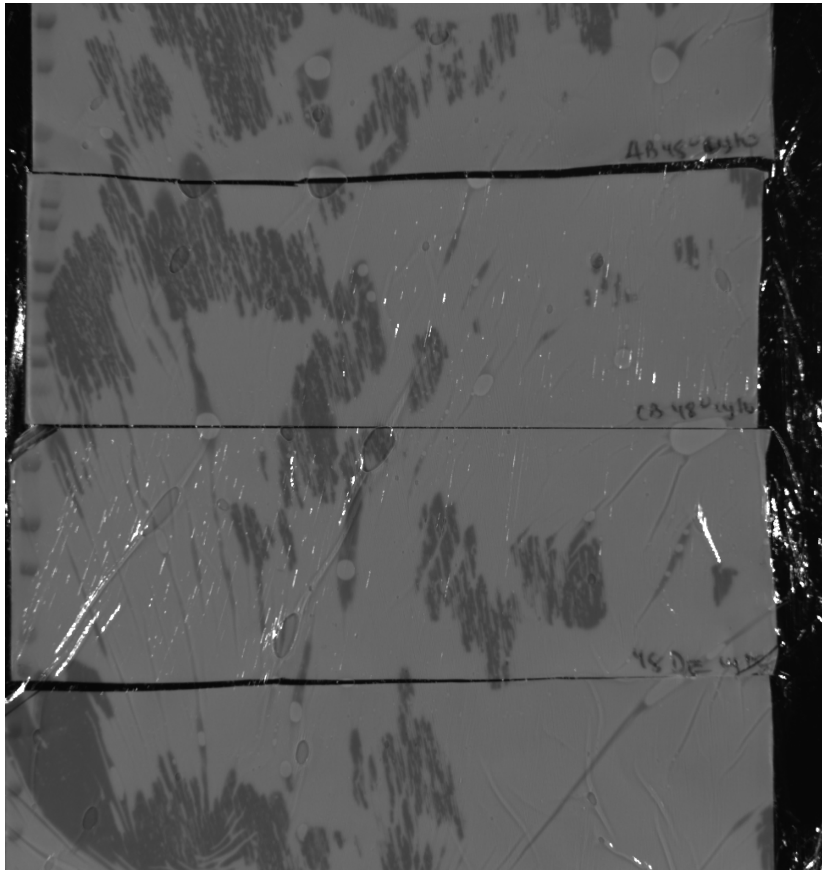

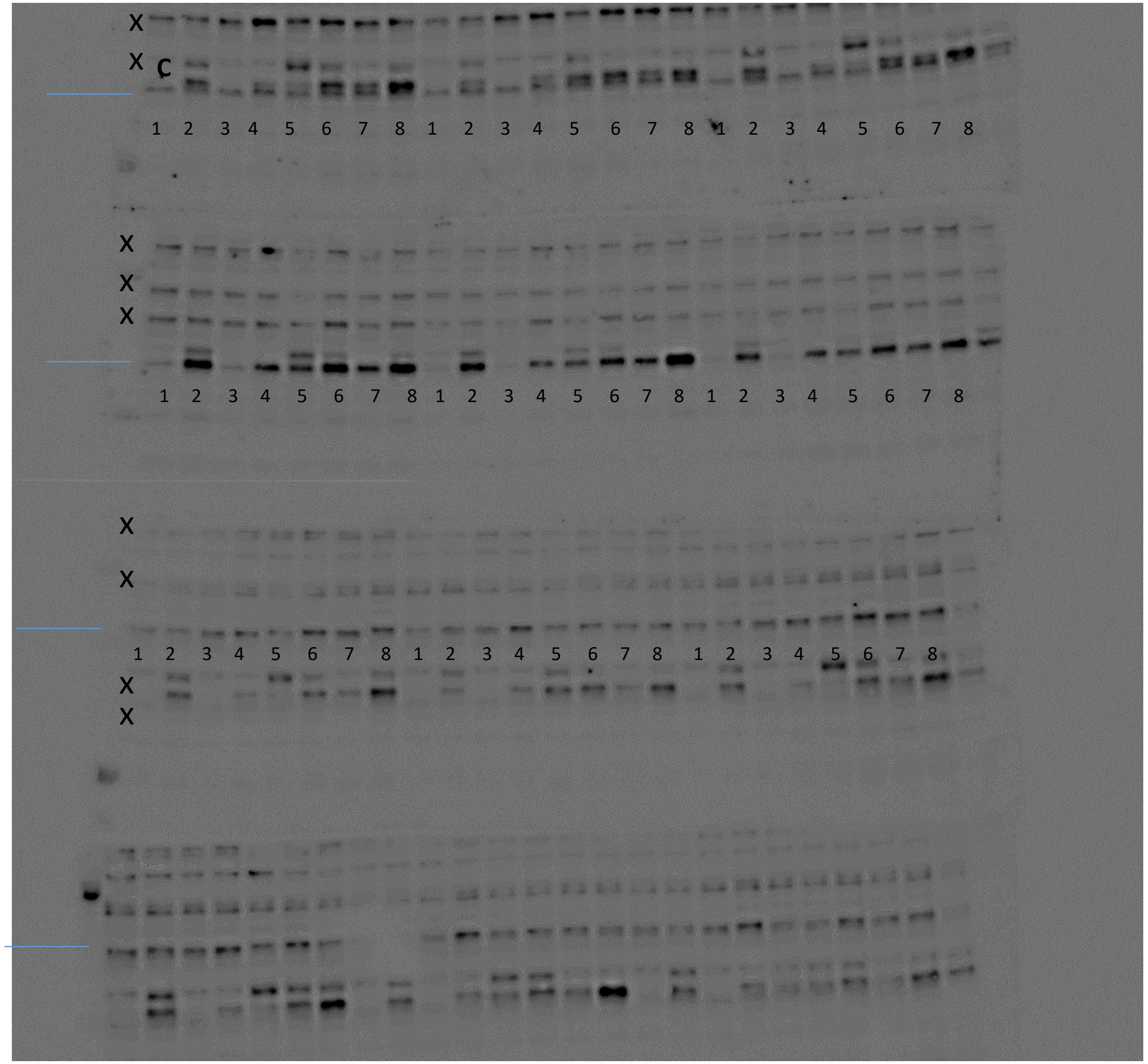

1: Control
2: IL1B
3: P4
4: Forskolin
5: F + IL1B
6: P4 + IL1B
7: P4 + F
8: P4 + forskolin + IL1B

Supplementary Figure 7A MKP1 cytosolic (40 kDa) – additional samples

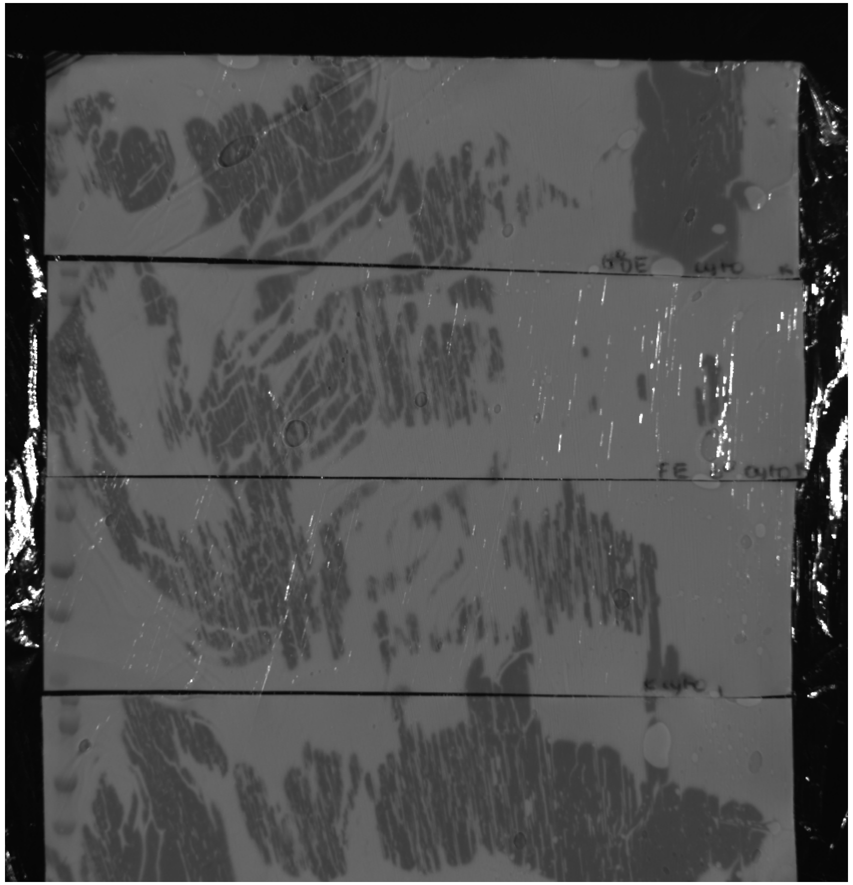

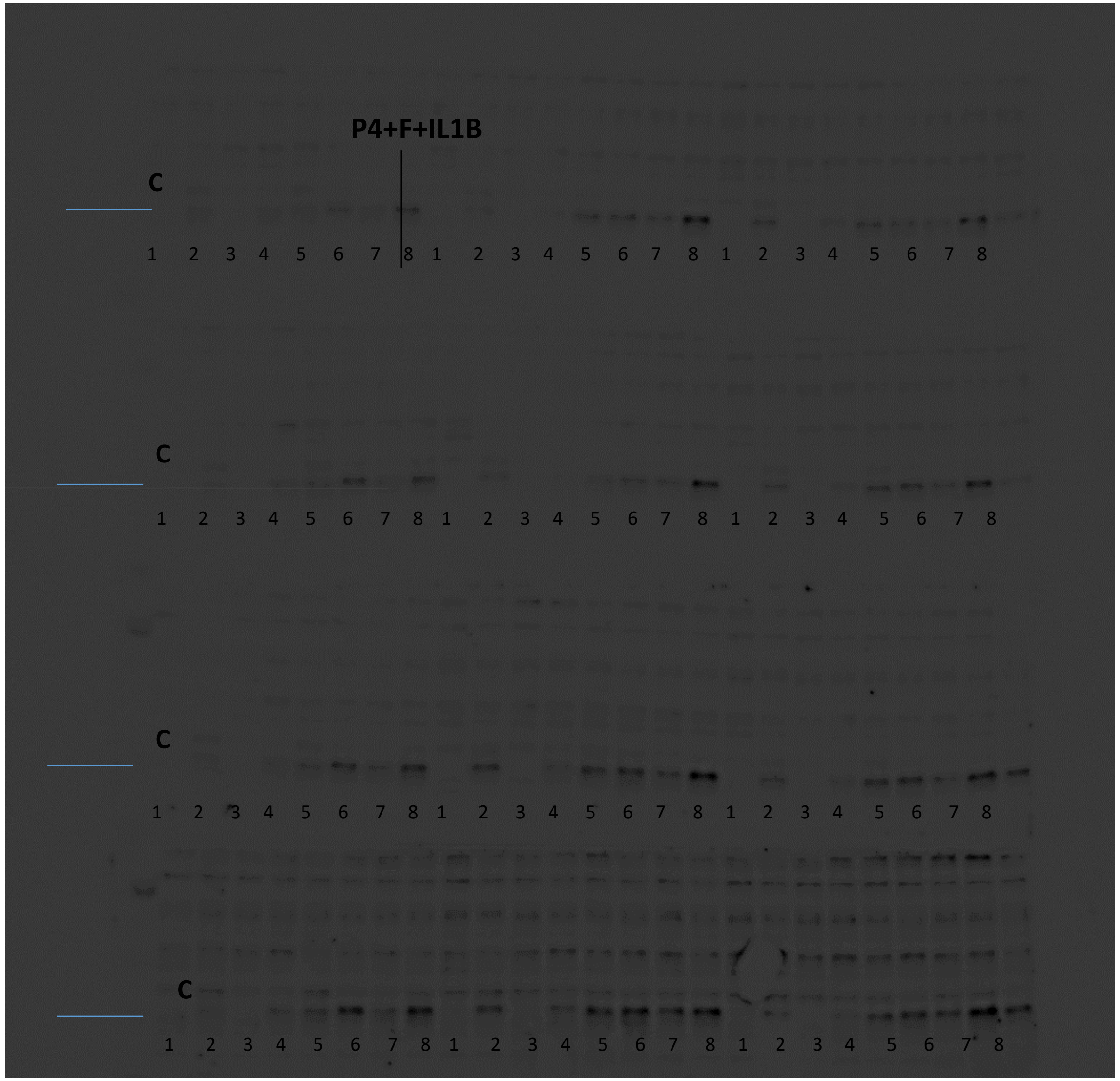

1: Control
2: IL1B
3: P4
4: Forskolin
5: F + IL1B
6: P4 + IL1B
7: P4 + F
8: P4 + forskolin + IL1B

Supplementary Figure 7B IKBα cytosolic (39 kDa)

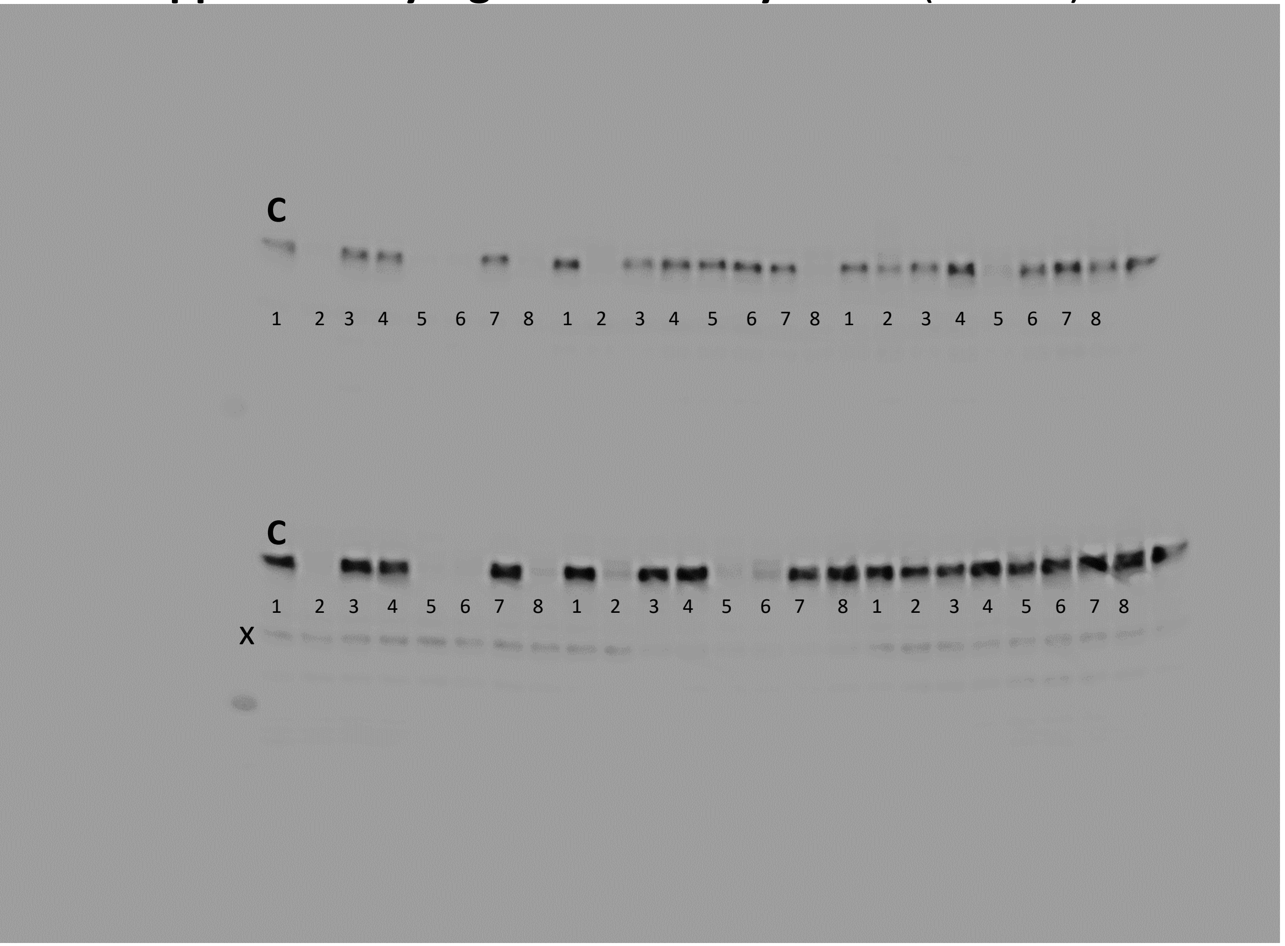

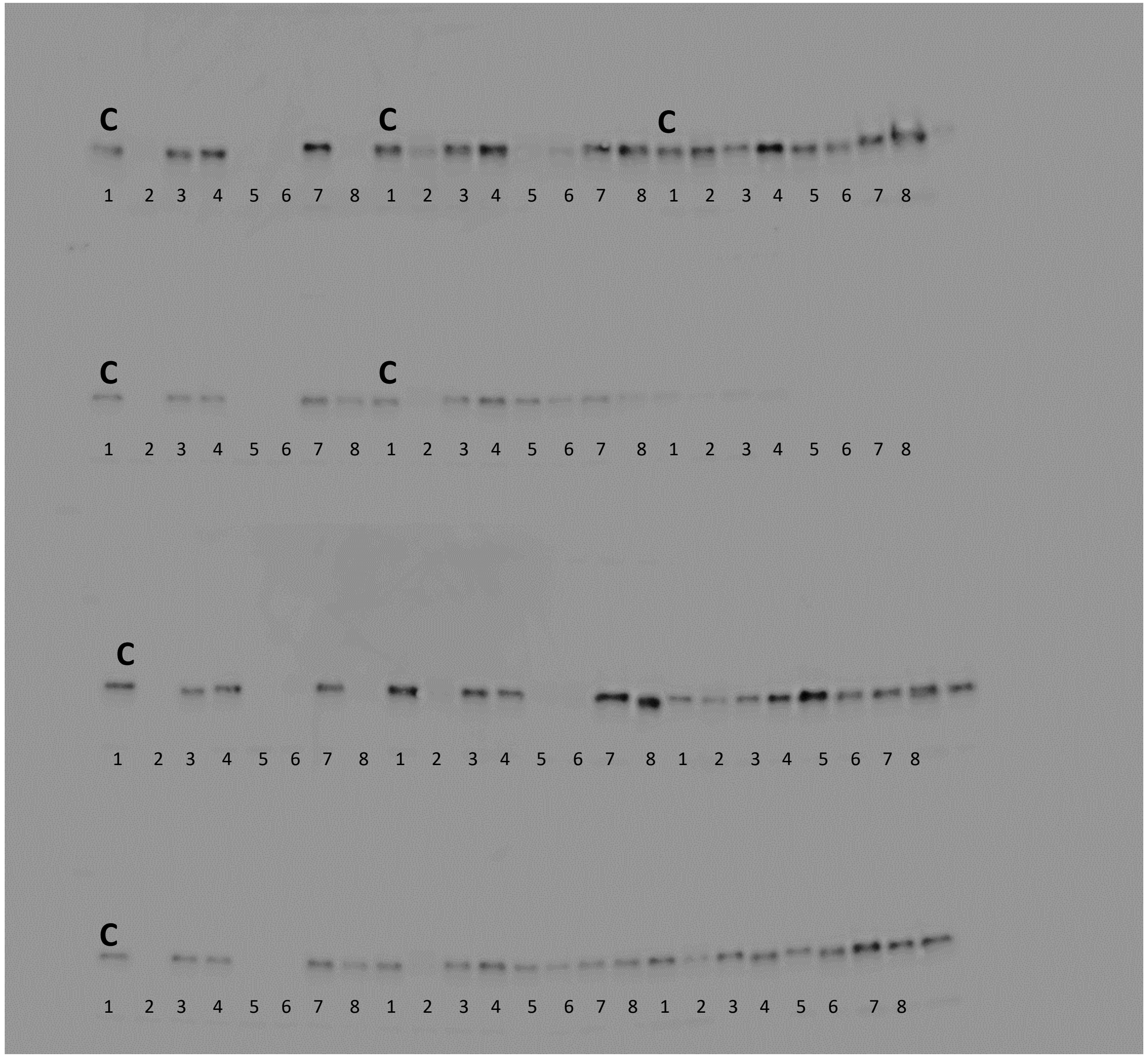

1: Control
2: IL1B
3: P4
4: Forskolin
5: F + IL1B
6: P4 + IL1B
7: P4 + F
8: P4 + forskolin + IL1B

Figure 7A&B α-tubulin (55 kDa) (for MKP1 & IKBα cytoplasmic - equivalent labelling as per slide 47 to 50)

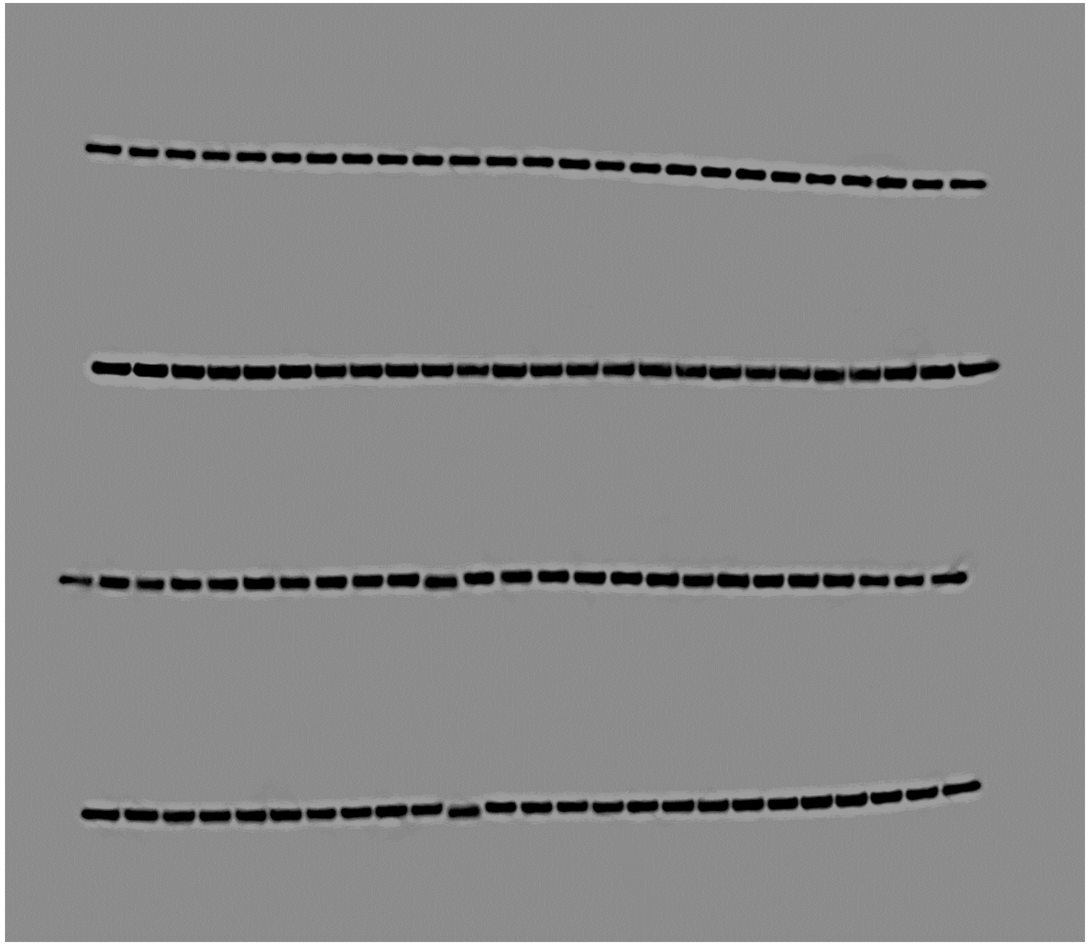

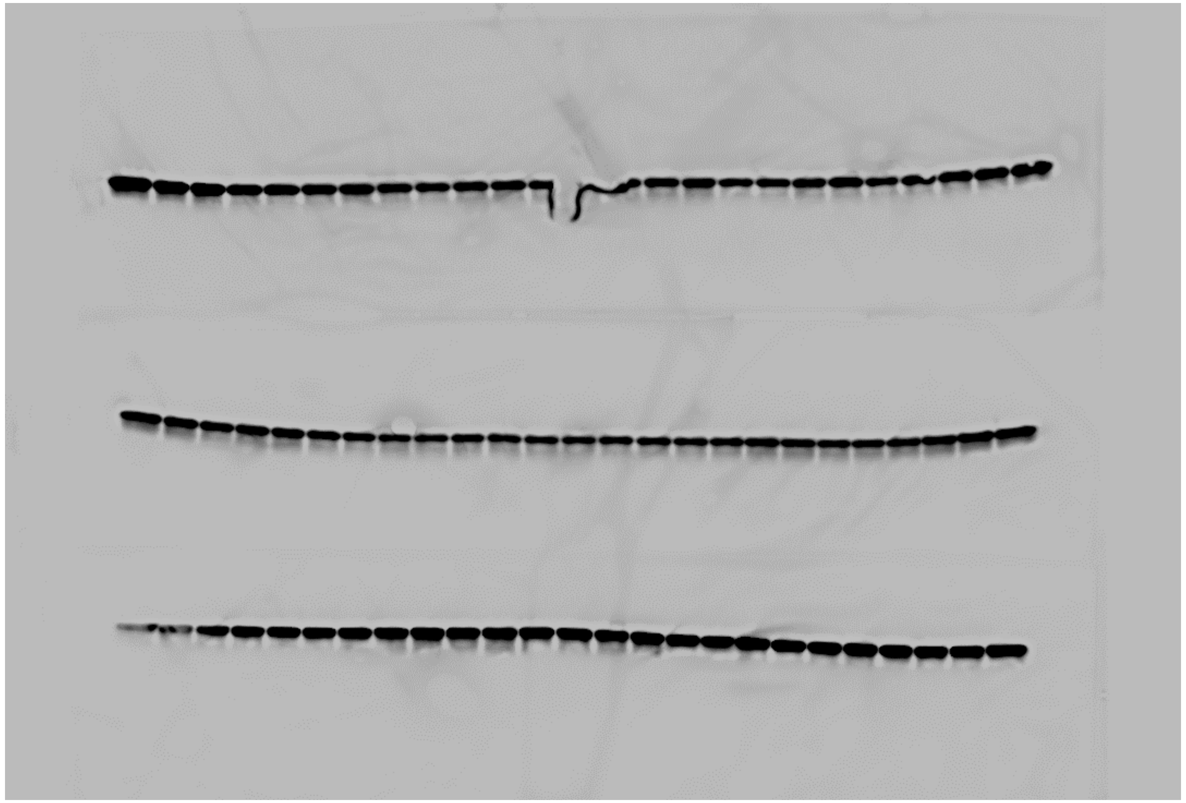

Supplement: S1 Raw images — (PDF) [file pone.0239937.s011.pdf]
